# Modelling dependent censoring in time-to-event data using boosting copula regression

**DOI:** 10.1007/s10985-025-09674-x

**Published:** 2025-10-21

**Authors:** Annika Strömer, Nadja Klein, Ingrid Van Keilegom, Andreas Mayr

**Affiliations:** 1 Department of Medical Biometry and Statistics, University of Marburg, Marburg, Germany; 2 Scientific Computing Center, Karlsruhe Institute of Technology, Karlsruhe, Germany; 3 ORSTAT, KU Leuven, Leuven, Belgium

**Keywords:** Copula, Distributional regression, Gradient boosting, Survival analysis, Variable selection

## Abstract

Survival analysis plays a pivotal role across disciplines, including engineering, economics, and social sciences—not just in biomedical research. In many of these applications, incomplete observations due to censoring are common, arising from limited follow-up periods, study dropouts, or administrative constraints. A standard assumption in such settings is that the censoring mechanism is independent of the survival process. This assumption primarily holds when censoring occurs at the end of the observation period. However, there may be dependence between event and censoring times. For example, if a patient’s health deteriorates and they withdraw due to poor prognosis, the time of censoring depends on their health status, leading to dependent censoring as sicker patients are censored earlier. To address such situations adequately in statistical analyses, we propose a model-based boosting approach using distributional copula regression. Our approach models the joint distribution of survival and censoring times by linking unknown marginal distributions through an unknown parametric copula. All distribution parameters of the resulting joint distribution are estimated simultaneously as functions of potentially different covariates. A key merit of the boosting approach is its data-driven variable selection, which is particularly important for such flexible models. Estimation remains feasible even for high-dimensional data with more covariates than observations, where classical estimation frameworks meet their limits. To investigate the performance of our method, we conduct a comprehensive simulation study, and demonstrate its practical application using a recent observational study analyzing the overall survival of patients with colon cancer. The data has a high proportion of right-censored observations without information on the cause of censoring.

**Supplementary Information:**

The online version contains supplementary material available at 10.1007/s10985-025-09674-x.

## Introduction

**Fig. 1:**
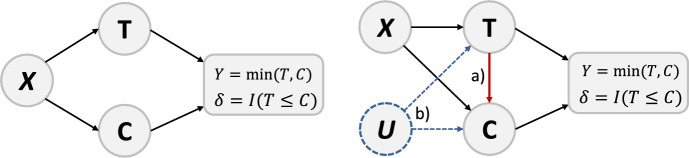
Graphical illustration of two survival scenarios with right-censoring. In both scenarios, the event and censoring times *T* and *C* are conditional on the covariates $\varvec{X}$. Furthermore, *Y* is the observed survival time, $\delta$ is the event indicator and *U* is an unobserved confounder. The left-hand graph displays independent censoring and the right-hand graph dependent censoring: a) direct dependence from *T* to *C* and b) indirect dependence through the unmeasured covariate *U*

Time-to-event data analyses play a crucial role in various disciplines, including biostatistics (Collett 2003; Klein and Moeschberger 2005; Samoladas et al. 2010; Hassan et al. 2018; De Bin and Stikbakke 2023; Srujana et al. 2024; Stijven et al. 2024). Their focus is on studying the time to the occurrence of an event of interest, such as death, disease progression, treatment response or another specific endpoint. A common challenge is that patients in a study may not experience the event of interest within the study period or may be lost to follow-up before the event occurs due to study dropout. Consequently, censored observations are an inherent and natural characteristic of survival data. Most widely used approaches, such as the Kaplan-Meier estimator (Kaplan and Meier 1958) or the Cox proportional hazards model (Cox 1972), can handle time-to-event data with censored observations, including the most common form of right-censoring. In these approaches, a key assumption is that the survival time *T* and censoring time *C* are statistically independent conditional on the covariates. This is illustrated in the left part of Fig. 1, where *C* provides no information about *T* given the covariates ${\varvec{X}}$ and vice versa.

However, this assumption is questionable in many situations. For example, if patients withdraw from a medical trial because their health condition is deteriorating, approaches that assume independent censoring could lead to biased results (Huang and Zhang 2008). This is due to a direct link between the survival and censoring times as illustrated in case a) in the right of Fig. 1. Thus, if we assume that sicker patients drop out of the study due to poor health, censored patients are more likely to be sicker than the non-censored patients and the survival time of the patients may be overestimated. A further dependence can exist if it is caused by unobserved confounding variables, as indicated by case b) in the right of Fig. 1. Here, the unobserved variable *U* influences survival and censoring times. This could occur if a patient in a trial experiences side effects of a treatment that requires an alternative treatment, causing the patient to drop out of the study.

Several distinct approaches have been developed to account for dependent censoring. These include frailty models, which introduce random effects to capture unobserved heterogeneity, such as that caused by unmeasured covariates, and to induce dependence between survival and censoring times only indirectly through a shared frailty term (e.g., Huang and Wolfe 2002; Schneider et al. 2020). In contrast, copula-based approaches have become increasingly popular for directly modeling the dependence between survival and censoring times  (e.g., Emura and Chen 2016; Deresa and Van Keilegom 2020a, 2020b; Deresa et al. 2022; Midtfjord et al. 2022; Czado and Van Keilegom 2023; Deresa and Van Keilegom 2024b). By specifying a joint distribution through a copula function and marginal models, these methods allow for more flexible and explicit dependence structures, going beyond the implicit modeling of frailty-based approaches. The identifiability of copula models under dependent censoring, however, is challenging because the right-censoring mechanism obviously prevents the simultaneous observation of (*T*, *C*) and thus obscures the direct relationship between *T* and *C* in the observed data (Tsiatis 1975; Crowder 1991). To ensure model identifiability, some authors assume that the copula is known. The first contribution in this direction was made by Zheng and Klein (1995), who proposed a generalization of the Kaplan-Meier estimator, the so-called copula-graphic estimator. This method was further explored by Rivest and Wells (2001) in the context of Archimedean copulas, assuming a known copula. Since then, numerous approaches have been developed in this vein (see among others, Braekers and Veraverbeke 2005; Huang and Zhang 2008; Chen 2010; Sujica and Van Keilegom 2018; Emura and Chen 2018; Deresa and Van Keilegom 2021). For instance, Chen (2010) extended the copula framework to semiparametric transformation models, using a known copula to characterize the association between survival and censoring times. While this method offers considerable flexibility for modeling marginal distributions, the assumption of a fully known copula function is often unrealistic in practical biostatistical applications. Czado and Van Keilegom (2023) recently proved that a known copula is not necessary for identifying the joint distribution of *T* and *C*. They proposed a model based on a parametric copula for the relationship between *T* and *C* along with parametric marginal distributions. However, a limitation is that covariates cannot be included into the model. Deresa et al. (2022) addressed this by exploring a copula-based method for bivariate data with left truncation and dependent right censoring, including multiple covariates but only for the margins.

To develop a framework that does not require the copula to be known, while allowing to incorporate covariates for all model parameters (in the spirit of generalized additive models for location, scale and shape, Rigby and Stasinopoulos 2005), we propose a model-based boosting approach for dependent censoring in survival analysis via distributional copula regression. Building on the work of Czado and Van Keilegom (2023) and Deresa et al. (2022), our method utilizes a parametric copula combined with arbitrary parametric marginal distributions. Estimation is carried out via statistical boosting, which facilitates the simultaneous modelling of all distribution parameters as functions of potentially different sets of covariates. Our boosting algorithm is based on the work of Hans et al. (2023), who proposed a general framework for boosting in distributional copula regression.

Our approach comes with three particular merits. First, the dependence between survival and censoring times can be modelled and explained through covariates. This may provide deeper insights and a better understanding of the underlying relationship between survival and censoring times. Second, our approach can handle high-dimensional cases where the number of covariates exceeds the number of observations ($p > n$). For such settings, most classical approaches are no longer feasible. Third, the boosting approach has the advantage of including a data-driven variable selection mechanism. This feature is particularly beneficial when dealing with many potential predictors, ensuring that only the most relevant variables are included without compromising predictive power.

Using a recent observational study from oncology that investigates the overall survival of patients with colon cancer we demonstrate the merits of our approach in practical research. The routine data were gathered from a specialized cancer center and include potential predictors such as common clinical variables related to the tumor, patient demographics, and treatment (Seipp et al. 2021). This dataset represents a typical scenario encountered in biostatistical applications, characterized by a relatively high proportion of right-censored observations. Since the censoring mechanism is inherently unknown, it is impossible to determine whether the censoring is dependent or independent. However, in observational studies based on routine data, dependent censoring is more likely to occur due to various factors; for example, both patients with advanced disease and better health are more likely to miss follow-up visits or withdraw from the study (Howe et al. 2010). Nevertheless, the Cox proportional hazards model or a parametric accelerated failure time (AFT) model (Wei 1992; Heller 2024) are often still the methods of choice. We examine how the results of the analysis differ when accounting for associations between survival and censoring times, and how these associations vary with the covariates.

The structure of this article is as follows: Sect. 2 presents distributional copula regression for dependent censoring in time-to-event data and how to perform estimation based on model-based boosting. In Sect. 3, we investigate the performance of the new boosting approach in a simulation study with different scenarios—including conditional independent censoring. We apply our approach to colon cancer data and present the results in Sect. 4 before we finally discuss strengths and limitations of our approach in Sect. 5. Supplementary Materials (SM) contain further simulations and additional results for the application.

## Model and Methods

### Model specification

Let *T* and *C* be the survival and censoring times, respectively. Based on the assumption of random right-censoring, we observe $Y = \text {min}(T,C)$ and $\delta = \mathcal {I}(T \le C)$, where $\mathcal {I}(A)=1$ if *A* is true and zero otherwise. Let furthermore $\varvec{X}=\varvec{x}\in \mathbb{R}^p$ denote the covariate vector. In the following, we allow for dependence between *T* and *C* and model this dependence utilizing a one-parameter copula $\mathcal {C}(\cdot ,\cdot \mid \theta )$ with parameter $\theta \in \mathbb{R}$. We assume that the marginal distributions $F_T$ and $F_C$ are non-negative, continuous with parametric densities $f_T(\cdot \mid \varvec{\theta }_T)$ and $f_C(\cdot \mid \varvec{\theta }_C)$, respectively; and $\varvec{\theta }_T\in \mathbb{R}^{K_T}$, $\varvec{\theta }_C\in \mathbb{R}^{K_C}$ are the respective distribution parameters.

To account for the covariate information $\varvec{x}$ in a regression setting, we relate the vector of model parameters $\varvec{\alpha }= (\varvec{\theta }_T^\top , \varvec{\theta }_C^\top , \theta )^T$ to covariates, that is, $\varvec{\alpha }\equiv \varvec{\alpha }(\varvec{x})$. Then, the conditional version of Sklar’s theorem (Patton 2006) allows to write the joint cumulative conditional distribution function (CDF) of *T* and *C* given $\varvec{x}$ as

$$ F_{T,C}(t,c\mid \varvec{\alpha }(\varvec{x})) = \mathcal {C}\{F_T(t\mid \varvec{\theta }_T(\varvec{x})), F_C(c\mid \varvec{\theta }_C(\varvec{x}))\mid \theta (\varvec{x})\}. $$

In practice however, we do observe $(Y,\delta )$ and the following proposition is an extension of Theorem 3.3 in Deresa et al. (2022), where we also allow the copula parameter to depend on the covariates.

#### Proposition 1

Let *Y*, $F_T$, $F_C$, $f_T$, $f_C$ and $\varvec{\alpha }$ be defined as above. Then, $F_Y(y\mid \varvec{\alpha }(\varvec{x}))=F_T(y\mid \varvec{\theta }_T(\varvec{x}))+F_C(y\mid \varvec{\theta }_C(\varvec{x}))-\mathcal {C}\{F_T(y\mid \varvec{\theta }_T(\varvec{x})), F_C(y\mid \varvec{\theta }_C(\varvec{x}))\mid \theta (\varvec{x})\}$ and

1 $$\begin{aligned} \begin{aligned} f_Y(y\mid \varvec{\alpha }(\varvec{x}))&=f_T(y\mid \varvec{\theta }_T(\varvec{x}))\left[ 1-h_{C|T}\{F_{C}(y\mid \varvec{\theta }_C(\varvec{x}))\mid F_{T}(y\mid \varvec{\theta }_T(\varvec{x}));\theta (\varvec{x}) \}\right] \\&\quad +f_C(y\mid \varvec{\theta }_C(\varvec{x}))\left[ 1- h_{T|C}\{F_{T}(y\mid \varvec{\theta }_T(\varvec{x}))\mid F_{C}(y\mid \varvec{\theta }_C(\varvec{x}));\theta (\varvec{x}) \}\right] \end{aligned}\end{aligned}$$

holds for the distribution and density of *Y*, respectively, where $h_{C|T}\{F_{C}(y\mid \varvec{\theta }_C(\varvec{x}))\mid F_{T}(y\mid \varvec{\theta }_T(\varvec{x}));\theta (\varvec{x}) \}$ and $h_{T|C}\{F_{T}(y\mid \varvec{\theta }_T(\varvec{x}))\mid F_{C}(y\mid \varvec{\theta }_C(\varvec{x}));\theta (\varvec{x}) \}$ are the conditional distribution functions of $T\mid C$ and $C\mid T$, respectively. These can be expressed in terms of their associated copula as

$$\begin{aligned} h_{C|T}\{F_{C}(y\mid \varvec{\theta }_C(\varvec{x}))\mid F_{T}(y\mid \varvec{\theta }_T(\varvec{x}));\theta (\varvec{x}) \}&= \frac{\partial }{\partial u}\mathcal {C}\lbrace u,v\mid \theta (\varvec{x})\rbrace |_{u=F_T(t\mid \varvec{\theta }_T(\varvec{x})), v=F_C(c\mid \varvec{\theta }_C(\varvec{x}))}\\ h_{T|C}\{F_{T}(y\mid \varvec{\theta }_T(\varvec{x}))\mid F_{C}(y\mid \varvec{\theta }_C(\varvec{x}));\theta (\varvec{x}) \}&= \frac{\partial }{\partial v}\mathcal {C}\lbrace u,v\mid \theta (\varvec{x})\rbrace |_{u=F_T(t\mid \varvec{\theta }_T(\varvec{x})), v=F_C(c\mid \varvec{\theta }_C(\varvec{x}))}. \end{aligned}$$

To ensure model identifiability, we focus on parametric margins and one-parameter copula functions, more precisely on log-normal and Weibull-distributed margins that are identifiable with the Clayton, Gaussian and Gumbel copulas, as was shown in Czado and Van Keilegom (2023).

#### Predictor specification

To allow for covariate dependent distribution parameters, we associate each element of $\varvec{\alpha }(\varvec{x})=(\theta _{T,1}(\varvec{x}),\ldots ,\theta _{T,K_T}(\varvec{x}),\theta _{C,1}(\varvec{x}),\ldots ,\theta _{C,K_C}(\varvec{x}), \theta (\varvec{x}))^\top \equiv (\alpha _1,\ldots ,\alpha _K)^\top$, $K=K_T+K_C+1$ to structured additive predictors $\eta _k$ via parameter-specific link functions $g_k$, such that $g_k(\alpha _k) = \eta _k, k = 1,\dots ,K$. The general idea of these structured additive predictors is e.g., described in Wood (2017) and assumes that each $\eta _k$ is of the form $\eta _k = \beta _{0k} + \sum _{j= 1}^{p_k} s_{jk}(\varvec{x}_{jk})$, where $\beta _{0k}$ are the intercepts and the $s_{jk}, j = 1,\dots , p_k$ represent the $p_{k}$ functional effects of parameter-specific covariate subvectors $\varvec{x}_{jk}\subset \varvec{x}$ in distribution parameter *k*. In this paper, we focus on linear effects that can be represented by $s_{jk}(\varvec{x}_{jk}) = x_{jk}^T \beta _{jk}$, where $\varvec{x}_{jk}$ is a covariate subset of $\varvec{x}$ for the parameter $\alpha _k$ and $\beta _{jk}$ are the regression coefficients, but other effects, such as nonlinear effects of univariate continuous covariates or spatial effects can be cast into this framework  (see again, e.g., Wood 2017).

### Estimation via model-based boosting

#### Background on boosting

For estimation, we resort to model-based boosting. The concept of boosting originated from machine learning (Freund 1995; Freund and Schapire 1997), and was later adapted to statistical modelling (Friedman et al. 2000, 2001). This statistical view on boosting formed the foundation for component-wise gradient boosting with regression-type base-learners with all its extensions for various objectives (Bühlmann and Hothorn 2007), which was later also referred to as *statistical or model-based boosting* (Mayr et al. 2014, 2017). The basic idea is to iteratively fit regression-type base-learners one-by-one to the negative gradient of a pre-specified loss function, which in our case corresponds to the negative log-likelihood, and the base-learners are the different linear effects. In every iteration only the best-fitting base-learner is updated and added to the respective current regression predictor (see Hofner et al. (2014), for a detailed overview). This procedure is repeated until the final stopping iteration $m_\mathrm{{stop}}$ is reached and the result can be somewhat compared to that of $L_1$ penalized lasso regression (Tibshirani 1996; Hepp et al. 2016).

#### Estimation procedure

Assume that we have an independent and identical distributed sample $\mathcal {D} = \{(y_i,\delta _i, \varvec{x}_i), i = 1,\dots , n \}$. Then, using (1), the joint log-likelihood is given by

$$\begin{aligned} \ell (\varvec{\alpha };\mathcal {D})&= \sum _{\delta _i = 1} \text {log}(f_{T}(y_i\mid \varvec{\theta }_T(\varvec{x}_i))\left[ 1 - h_{C|T}\{F_{C}(y_i\mid \varvec{\theta }_C(\varvec{x}_i))\mid F_{T}(y_i\mid \varvec{\theta }_T(\varvec{x}_i)) ;\theta (\varvec{x}_i)\} \right] ) \\&\quad + \sum _{\delta _i = 0} \text {log}(f_{C}(y_i\mid \varvec{\theta }_C(\varvec{x}_i))\left[ 1- h_{T|C}\{F_{T}(y_i\mid \varvec{\theta }_T(\varvec{x}_i))\mid F_{C}(y_i\mid \varvec{\theta }_C(\varvec{x}_i));\theta (\varvec{x}_i) \} \right] ). \end{aligned}$$

Our model is integrated into the boosting distributional copula regression framework of Hans et al. (2023). Here, all distribution parameters are modelled simultaneously, i.e., in every iteration all partial derivatives $u_k = -\partial \ell /\partial \alpha _k$ of the negative log-likelihood $-\ell$ with respect to the different distribution parameters $\alpha _k$ are calculated and each base-learner $b_{jk}\equiv x_{jk}^\top \beta _{jk}$ is separately fitted to the respective gradients. For each distribution parameter, the best-fitting base-learner $b_{jk}^*$ is identified, i.e., the one with the highest loss reduction, and compared among all distribution parameters. Only a small proportion (step-length $\nu$) of the overall best-performing base-learner $\nu \times b_{jk}^*$ is added to the respective predictor $\eta _k$ in every iteration, using a non-cyclic version of the basic boosting algorithm (see Thomas et al. 2018, for details). The step-length $\nu$ is set to a small fixed value within the range of $0< \nu < 1$ (Schmid and Hothorn 2008). For boosting copula regression, Hans et al. (2023) suggest a value of $\nu =0.01$ and we follow this default choice.

#### Benefits of boosting

Boosting can handle high-dimensional data (that is, $p>n$) and is thus an attractive alternative to classical inference methods for statistical models in such situations. The algorithm selects only one base-learner in each iteration, building up predictors step-by-step. At each step, the base-learner that achieves the largest empirical risk reduction is updated, allowing its effect to accumulate over iterations. Base-learners contributing most to risk minimization are selected repeatedly, while those never selected are effectively excluded from the final model. This data-driven variable selection, conducted simultaneously across all additive predictors, is controlled by the stopping iteration $m_\mathrm{{stop}}$. The number of boosting iterations $m_\mathrm{{stop}}$ determines the complexity of the final model; a higher number of iterations may include more variables, some with negligible effects that may reflect noise rather than signal. To balance sparsity and prediction accuracy, $m_\mathrm{{stop}}$ is typically optimized by cross-validation, resampling techniques, or using an additional data set (if available). The latter usually yields comparable stability in the selected stopping iteration and resulting model at lower computational cost than cross validation or resampling. In addition to encouraging the sparsity of the resulting models, the optimization of $m_\mathrm{{stop}}$ also leads to the prevention of overfitting, since the algorithm usually stops before convergence (also referred to as early stopping, Mayr et al. (2012)). For enhanced sparsity, post-hoc refinement via stability selection (Meinshausen and Bühlmann 2010) or deselection (Strömer et al. 2022, 2025) can be applied.

We denote our approach *CopBoostDepCens* in the remainder of the paper.

## Simulations

We conducted a detailed simulation study to evaluate the performance of CopBoostDepCens with regard to the following questions:

- Can CopBoostDepCens correctly estimate the corresponding distribution parameters and identify the respective active variables?
- How do different censoring rates influence the results?
- How does CopBoostDepCens compare to a model that assumes independent censoring?
- How does CopBoostDepCens perform in situations where the survival and censoring times are actually independent?

### Simulation design

To represent lower, no and upper tail dependence scenarios, we generate data using the Clayton, Gaussian and Gumbel copulas. To ensure identifiability, we combine these with either log-normal or Weibull-distributed margins for both survival and censoring times. We denote the copula parameter with $\rho$, whereby we use $\rho ^{\star }$ and $\rho ^{\diamond }$ to distinguish between different copula parameters for specific scenarios. Additionally, $\mu _T, \sigma _T,\mu _C,\sigma _C$ represent the location and scale parameters of the marginal distributions of *T* and *C*, respectively. The corresponding true predictor specifications $\eta _., g_.$, $\cdot \in \lbrace \mu _T,\sigma _T,\mu _C,\sigma _C,\rho \rbrace$ are

$$\begin{aligned} g_{\mu _T}(\mu _T) = \eta _{\mu _T}&= \beta _{0\mu _T} + 2x_1 + x_3&g_{\sigma _T}(\sigma _T)&= \eta _{\sigma _T} = 0.7 + 0.7 x_3 \\ g_{\mu _C}(\mu _C) = \eta _{\mu _C}&= \beta _{0\mu _C} -x_2 + 1.5x_4&g_{\sigma _C}(\sigma _C)&= \eta _{\sigma _C} = 0.5x_2 \\ g_{\rho ^{\star }}(\rho ^{\star }) =\eta _{\rho ^{\star }}&= 2 + 1.5x_5&g_{\rho ^{\diamond }}(\rho ^{\diamond })&=\eta _{\rho ^{\diamond }} = 0.25 + 0.4x_1 - 0.6x_5, \end{aligned}$$

where $(\beta _{0\mu _T};\beta _{0\mu _C}) \in \lbrace (-1;0.8),(0.7;0.8),(1;-0.4) \rbrace$ for Weibull-distributed margins and $(\beta _{0\mu _T};\beta _{0\mu _C}) \in \lbrace (-0.9;0.8),(1;0.8),(1.5;-0.4) \rbrace$ for log-normal distributed margins. These values are chosen to mimic different average proportions of censoring of 20%, 50% and 80%, respectively. The covariates $x_1, \ldots , x_p$ are independently drawn from uniform distributions on $(-1,1)$. We fix the number of observations to $n = 1000$ but vary the number of available covariates across all model components. Specifically, we define $p^*$ as the total number of available covariates included across all additive predictors (i.e., across all model parameters), whereas $p_k$ denotes the number of covariates used in the *k*-th predictor (e.g. for one marginal distribution or the copula parameter). Since each of the $K=5$ distribution parameters in our model has its own additive predictor, the total number of covariates is $p^*=\sum _{k = 1}^K p_k$. In our simulations, the same set of covariates is used for all distribution parameters. We consider $p^* \in \{50,250,500,1000,2500\}$, representing different levels of noise variables without any influence. The high-dimensional case, $p^* = 2500$ is evaluated only in Setting 1. The following settings are examined:

**Setting 1(dependent censoring, positive association)**: This setting evaluates all combinations of margins and copulas, with the copula parameter $\rho$ modelled by $g_{\rho ^{\star }}(\rho ^{\star }) = \eta _{\rho ^{\star }}$. The average dependence between the margins, as measured by Kendall’s $\tau$, ranges from $\left[ 0.45; 0.94\right]$ for the Clayton copula, $\left[ 0.31; 0.96\right]$ for the Gaussian copula, and $\left[ 0.39; 0.97\right]$ for the Gumbel copula (depending on the realization of covariate $x_5$).
**Setting 2(dependent censoring, weaker and negative association)**: This setting specifically explores the Gaussian copula, with the copula parameter $\rho$ modelled by $g_{\rho ^{\diamond }}(\rho ^{\diamond }) = \eta _{\rho ^{\diamond }}$. This setting allows for negative dependence and exhibits lower dependence values compared to Setting 1. On average, Kendall’s $\tau$ is within $\left[ -0.43; 0.63\right]$.
**Setting 3(no association between censoring and survival times)**: In the independent setting, where $g_{\rho }(\rho ) = \eta _\rho =0$ (no dependent censoring) and thus the copula parameter $\rho$ does not depend on any covariates, we focus on the case with $p = 10$ covariates. Further results can be found in SM A and B.

In all settings, only the additive predictor for $\rho$ changes between the settings, while the other additive predictors for the marginal distributions remain the same. For each setting we generate 100 replicated data sets using the R package copula. The corresponding link functions for the marginal distributions and copulas are listed in Table 1. The runtime for all settings can be found in SM A and B.

**Table 1:** Link functions for the considered marginal distributions and copulas

	Parameter	Link function	
*Margins*			
Log-normal	Location	Identity	$g_{\mu _T}(u) = u$, $g_{\mu _C}(v) = v$
	Scale	Log	$g_{\sigma _T}(u) = \log (u)$, $g_{\sigma _C}(v) = \log (v)$
Weibull	Scale	Log	$g_{\mu _T}(u) = \log (u)$, $g_{\mu _C}(v) = \log (v)$
	Shape	Log	$g_{\sigma _T}(u) = \log (u)$, $g_{\sigma _C}(v) = \log (v)$
*Copulas*			
Clayton	Dependence	Log	$g_{\rho }(\rho ) = \log (\rho )$
Gaussian	Dependence	Inverse hyperbolic tangent	$g_{\rho }(\rho ) = \tanh ^{-1}(\rho )$
Gumbel	Dependence	Log shifted by 1	$g_{\rho }(\rho ) = \log (\rho - 1)$

#### Benchmark methods

We benchmark CopBoostDepCens with a similar model for survival analysis, namely the distributional AFT model (referred to in the following as AFT model), which does not take dependent censoring into account. The AFT model is fully parametric and allows for direct modelling of both the location and scale parameters as functions of covariates (in the spirit of generalized additive models for location, scale and shape, Rigby and Stasinopoulos 2005). We also estimate the AFT models with gradient boosting and the same settings and predictor specifications as CopBoostDepCens. As CopBoostDepCens also considers AFT-type distributions as margins, the distributional AFT model hence represents a very similar approach to ours, only that it focuses on the survival times *T* assuming independent censoring. The most popular model for survival analysis would be the Cox model. Due to its semi-parametric nature with unspecified baseline hazards, we do not consider it to be a fair competitor. However, additional results for the Cox models are available in SM A and B.

#### Performance metrics

To evaluate the predictive performance, we generate an additional test data set of equal sample size to assess the following metrics for each setting and method: Brier score and integrated absolute error. Let $S(t|\varvec{x}_i) = P(T>t|\varvec{x}_i)$ represent the true survival function at time *t*, indicating the probability that an individual with covariate vector $\varvec{x}_i$ survives beyond time *t*. The predicted survival function, derived from the model, is denoted with ${\hat{S}}(t|\varvec{x}_i)$.

The Brier score (BS) measures the accuracy of the predicted survival function ${\hat{S}}(t|\varvec{x}_i)$ and thus the calibration of the model by calculating the mean squared difference between predicted probabilities and actual outcomes. For right-censored data, the Brier score at time $t=Y_1,\ldots ,Y_n$ is computed as:

$$ \text {BS}(t) = \frac{1}{n} \sum _{i=1}^n \left[ \frac{{\hat{S}}(t|\varvec{x}_i)^2 \cdot \mathcal {I}(Y_i \le t, \delta _i =1)}{{\hat{G}}(Y_i)} + \frac{(1-{\hat{S}}(t|\varvec{x}_i))^2 \cdot \mathcal {I}(Y_i > t)}{{\hat{G}}(t)}\right] , $$

where ${\hat{G}}(t)$ is an estimate of the survival function $P(C>t)$ for the censoring time, defined as the probability that the censoring time *C* exceeds time *t*. The latter is typically estimated using the Kaplan-Meier estimator (Kaplan and Meier 1958; Graf et al. 1999), which is applied to the AFT model. However, with CopBoostDepCens, censoring times can directly be estimated from the model, which accounts for covariates, unlike the Kaplan-Meier estimator. Thus, for CopBoostDepCens, we replace ${\hat{G}}$ by ${\hat{G}}(t|\textbf{x}_i)$, which estimates $P(C > t|\textbf{x}_i)$. This is similar to the approach of Lillelund et al. (2025), which replaces the Kaplan-Meier estimator with a copula-graphic estimator for estimating *G*(*t*). Lower values for the Brier score indicate greater accuracy.

The integrated absolute error (IAE) measures the absolute error over time, weighting all errors equally and is defined by

$$\begin{aligned} \text {IAE} = n^{-1}\sum _{i=1}^n \int _0^{t_\mathrm{{max}}} |S(t|\varvec{x}_i) - {\hat{S}}(t|\varvec{x}_i)|dt \end{aligned}$$

where $S(t|\varvec{x}_i)$ denotes as before the true survival function and ${\hat{S}}(t|\varvec{x}_i)$ represents the predicted survival function at time *t* (Moradian et al. 2017). The largest value among $Y_1,\dots ,Y_n$ is denoted as $t_\mathrm{{max}}$. Results for the integrated Brier score and integrated squared error can be found in SM A and B.

Note that most standard evaluation metrics were originally developed under the assumption of independent censoring. When applied to settings with dependent censoring, they should be interpreted with caution. We therefore focus on the Brier score, adjusted to incorporate censoring, and the IAE, which remains valid regardless of the censoring mechanism.

#### Tuning and implementation details

The CopBoostDepCens models have been implemented as an add-on to the R package gamboostLSS, where also the AFT model is implemented. The stopping iteration $m_{\text{ stop }}$ is optimized by minimizing the empirical risk on an additional validation data set with $n_{\text {val}} = 1000$ observations. The step-length is set to $\nu =0.01$ for CopBoostDepCens and the AFT model for all distribution parameters (cf., Hans et al. 2023). All code required to replicate our results is available on GitHub: https://github.com/AnnikaStr/CopBoostDepCens.

### Results

In the following, we present the results for Weibull margins and the Gaussian copula for all settings. Results for the Clayton and Gumbel copulas for Setting 1 and Setting 2 with Weibull margins can be found in SM, Section A, whereas results with log-normal margins and all three copulas are given in Section B.

#### Setting 1 (dependent censoring, positive association)

**Fig. 2:**
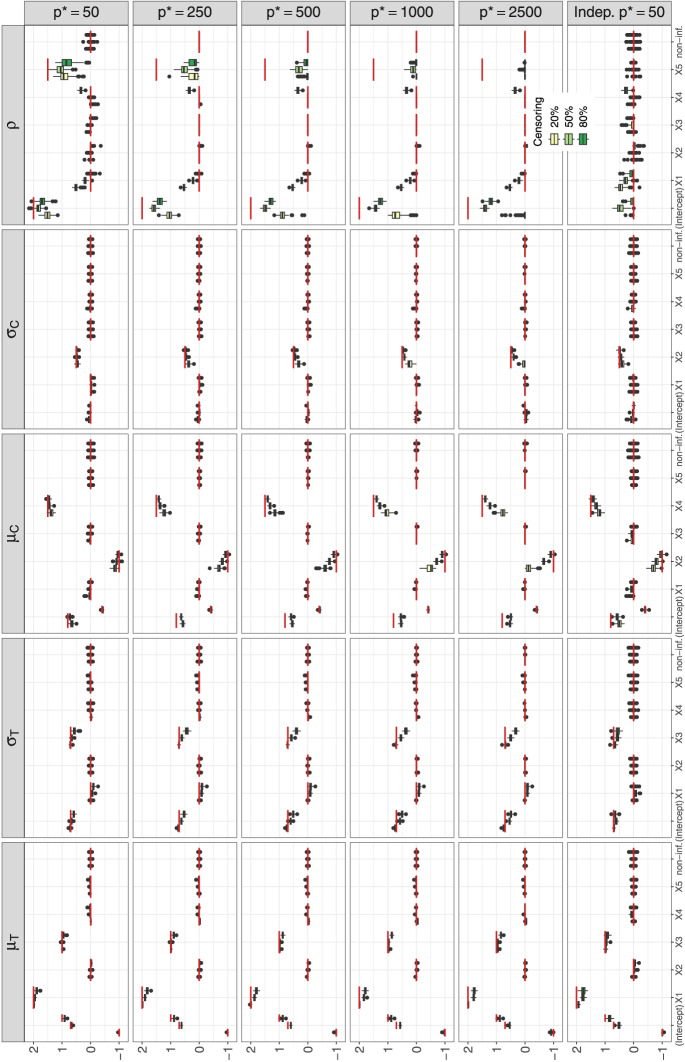
Simulation study. Boxplots of estimated coefficients of CopBoostDepCens on the 100 replicates. Results are shown for each distribution parameter (columns) of the Gaussian copula with Weibull-distributed margins for different numbers of noise variables (rows 1–5, Setting 1) as well as for Setting 3 with $p=10$ (row 6). The box colors represent the average proportions of censoring. The red horizontal lines show the true values for each

Figure 2 presents boxplots of coefficient estimates of the informative and non-informative (non-inf.) variables of CopBoostDepCens on the 100 simulation replicates. Results are shown for each distribution parameter (columns) of the Gaussian copula with Weibull-distributed margins for different numbers of noninformative variables (rows 1–5). The box colors represent the average proportions of censoring. The red horizontal lines show the true values of each corresponding coefficient. Missing boxplots indicate that the algorithm did not select the corresponding variables.

CopBoostDepCens effectively captures the true structure of informative variables for each parameter of the margins reasonably well, even with a large number of noise variables and high censoring rates. For instance, in the high-dimensional case of $p^* = 2500$, where $p^* > n$, the algorithm performs comparably to lower-dimensional settings in identifying informative variables. Only a slightly stronger shrinkage of effect estimates is observed. Estimates for non-informative variables are consistently shrunk to zero, with fewer false positives as $p^*$ increases. The true effects for the dependence parameter $\rho$ are also correctly identified but show stronger shrinkage as $p^*$ increases. In addition, $x_1$ is occasionally falsely selected for 20% and 50% censoring, with lower rates at 80% (see SM: Table A3).

Figure A1 (SM A.1.1) presents the coefficient estimates for the benchmark method: The truly informative variables for the survival time are correctly identified for both distribution parameters of the AFT models, but slightly overestimate their coefficients and include some noise variables, particularly those informative for censoring. Table 2 (rows 1–4) shows the means and standard deviations (SD) of the Brier score and IAE for performance evaluation. CopBoostDepCens consistently outperforms the AFT model in Brier scores, indicating better calibration and prediction accuracy. For the IAE, the AFT model performs best at 20% censoring, but CopBoostDepCens maintains stable results across varying covariates and censoring rates and performs best at 50% and 80% censoring. This can be explained by the fact that unlike the AFT model, CopBoostDepCens needs to estimate the censoring time, which is difficult when only 20% of the data is censored. Additionally, IAE is also calculated for the censoring time.

#### Setting 2 (dependent censoring, weaker and negative association)

Figure A2 in SM A.1.2 shows the coefficient estimates for CopBoostDepCens of Setting 2. The parameter estimates of the margins closely resemble those from Setting 1, despite lower and partly negative dependence, indicating correct identification of the informative variables. For the dependence parameter, the informative variables are also correctly detected with a slight overestimation of the intercept at 50% censoring and for the coefficient of $x_1$ at 20% censoring. This overestimation is expected because $x_1$ has a strong effect on $\mu _T$ and with 20% censoring, more events are observed, leading to a slightly overestimated effect on the dependence parameter. For the AFT model, only minor differences are observed: The AFT model correctly identifies informative variables for both distribution parameters, slightly overestimating the effect for $x_1$, which improves with more covariates (see SM A.1.2, Fig. A3). CopBoostDepCens achieves the lowest Brier scores, while the AFT model consistently performs better in IAE, which, as for Setting 1, can be attributed to the fact that CopBoostDepCens needs to estimate the censoring time (Tables A4 and A5, SM A.1.2).

#### Setting 3 (no association between censoring and survival times)

Setting 3 investigates how the model performs under conditional independent censoring, where the censoring times depend only on known covariates but not on the survival times. Similar to the observations in the dependent settings, CopBoostDepCens performs well in identifying the informative variables across different censoring rates for both survival and censoring time (see Fig. 2; row 6). However, the model includes some noise variables in the dependence parameter. The AFT model provides rather accurate estimates without overestimation (Fig. A4, SM A.1.3). This is also reflected in the IAE in Table 2, which are consistently lowest for the AFT model and do not increase as much with higher censoring as in Setting 1. The values for CopBoostDepCens are similar to the dependent settings, including for the censoring time. CopBoostDepCens remains superior for the Brier score, showing no noticeable change from the dependent settings.

### Overall summary of simulation results

CopBoostDepCens demonstrates a favorable performance in both identifying and estimating informative variables across various scenarios. It effectively identifies the relevant variables for each distribution parameter, even under more challenging conditions characterized by weaker and negative dependence. CopBoostDepCens consistently captures the true structure of the data across different levels of censoring and thus provides robust results for the considered censoring rates. In addition, performance remains reliable even when survival and censoring times are truly independent.

**Table 2:** Simulation study

		**Brier score**		**Integrated absolute error**		
				Survival time		Censoring time
	Censoring (%)	Copula	AFT	Copula	AFT	Copula
$p^*=$ 50	20	**0.07 (0.00)**	0.10 (0.13)	0.94 (0.09)	**0.13 (0.02)**	0.88 (0.12)
	50	**0.08 (0.01)**	0.13 (0.18)	**1.13 (0.05)**	1.84 (0.26)	2.05 (0.13)
	80	**0.06 (0.02)**	0.10 (0.16)	**1.23 (0.16)**	3.87 (1.00)	1.41 (0.19)
$p^*=$ 250	20	**0.07 (0.00)**	0.11 (0.16)	0.94 (0.08)	**0.13 (0.02)**	0.84 (0.10)
	50	**0.09 (0.02)**	0.12 (0.16)	**1.15 (0.05)**	1.59 (0.23)	1.99 (0.11)
	80	**0.06 (0.01)**	0.11 (0.18)	**1.29 (0.19)**	2.89 (0.81)	1.37 (0.19)
$p^*=$ 500	20	**0.07 (0.00)**	0.10 (0.14)	0.93 (0.08)	**0.12 (0.02)**	0.83 (0.11)
	50	**0.10 (0.02)**	0.13 (0.17)	**1.19 (0.06)**	1.42 (0.21)	2.02 (0.12)
	80	**0.06 (0.02)**	0.08 (0.09)	**1.30 (0.18)**	2.30 (0.55)	1.34 (0.14)
$p^*=$ 1000	20	**0.07 (0.00)**	0.10 (0.14)	0.93 (0.07)	**0.13 (0.02)**	0.87 (0.10)
	50	**0.10 (0.01)**	0.13 (0.18)	** 1.24 (0.08)**	1.27 (0.20)	2.03 (0.13)
	80	**0.06 (0.01)**	0.10 (0.15)	**1.34 (0.18)**	1.90 (0.51)	1.37 (0.17)
$p^*=$ 2500	20	**0.07 (0.00)**	0.11 (0.15)	0.91 (0.08)	**0.13 (0.02)**	1.00 (0.13)
	50	**0.07 (0.00)**	0.11 (0.15)	1.32 (0.08)	**1.16 (0.16)**	2.07 (0.13)
	80	**0.06 (0.01)**	0.10 (0.12)	**1.35 (0.20)**	1.57 (0.41)	1.35 (0.19)
Indep. $p^*=$ 50	20	**0.07 (0.00)**	0.12 (0.15)	0.88 (0.09)	**0.04 (0.01)**	0.81 (0.13)
	50	**0.08 (0.01)**	0.17 (0.21)	1.42 (0.19)	**0.25 (0.06)**	1.99 (0.21)
	80	** 0.05 (0.02)**	0.11 (0.10)	1.18 (0.32)	**0.39 (0.16)**	1.26 (0.23)

Brier score (SD) and integrated absolute error (SD) for CopBoostDepCens and AFT models on the 100 replicates of the Gaussian copula with Weibull-distributed margins for different numbers of noise variables (rows 1–5, Setting 1) as well as for Setting 3 with $p^*=50$ (row 6). The best-performing model for each metric is highlighted in bold

In terms of predictive performance, CopBoostDepCens also performs well. When compared to a model that assumes independent censoring, such as the AFT model, the results are partially comparable, particularly for the integrated absolute error which focuses on survival time alone. However, the AFT model is limited by its exclusive focus on survival time, whereas CopBoostDepCens accounts for both survival and censoring times, as well as their potential dependence. In scenarios with only low censoring rates and/or weak dependencies between *T* and *C*, the AFT model seems to be suitable if the focus is solely on survival time. However, the strength of CopBoostDepCens lies in its ability to capture complex relationships between survival and censoring time that simpler models may miss, providing a broader perspective that is particularly valuable in scenarios that require a deeper understanding of their interplay. Note that, interestingly, our approach exhibits a decreased runtime in high-dimensional settings, as overfitting tends to occur earlier, forcing the algorithm to stop early.

## Observational study on survival of colon cancer patients

We illustrate the application of our method via an observational study investigating the overall survival of colon cancer patients after surgery based on routine data.

### Data and model building

We analyse data that contain information on $n = 546$ patients listed in a registry of a local German acute care hospital. All enrolled patients underwent the surgical resection of the affected part of the intestine with radical regional excision of adjacent lymph node stations, following the corresponding guidelines (Seipp et al. 2021). The data are publicly available in the R package dirttee (Seipp and Otto-Sobotka 2022).

**Fig. 3:**
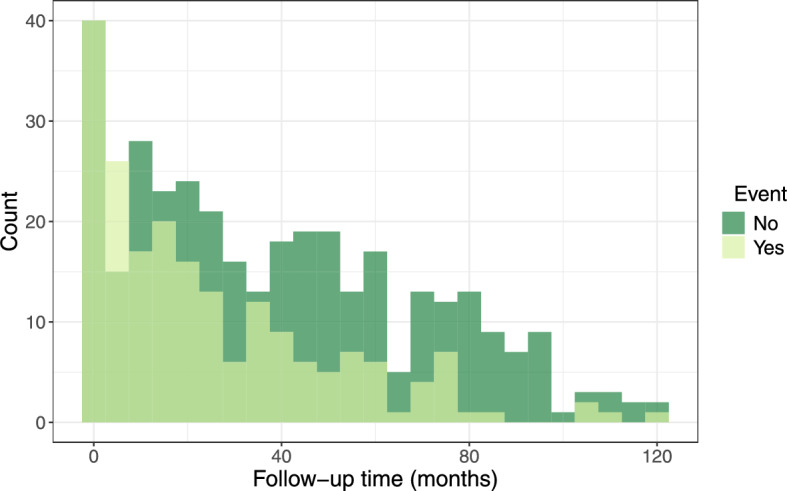
Observational study. Histogram of the follow-up time in months for patients with event (light green) and without event (dark green)

In what follows, we focus on the outcome of overall survival since surgery. The event was observed for 201 patients, while 345 patients were right-censored. Median follow-up time was 26.6 months, for a histogram of the follow-up times for patients with and without event, see Fig. 3. The high censoring rate ($63.1\%$) is a most likely result of the relatively good prognosis for colon cancer patients and the short follow-up times. There is no information, however, on the individual causes of censoring, but as this is an observational study, it is very likely that patients drop out of the study for a reason related to their survival time. The following clinical variables are considered: chemotherapy (yes or no), ASA score (general health status, mild or severe), UICC cancer stage (I–IV, higher stage means a further progressed tumor), age of the patient, LNE (number of pathologically examined lymph nodes during surgery), LNR (lymph node ratio, number of cancerous lymph nodes divided by the number of examined lymph nodes), sex, R status (residual tumor after surgery, yes or no) and preexisting cancer (yes or no). A more detailed description of the data can be found in Seipp et al. (2021).

#### Model selection

Our aim is to model the distribution of observed events and censoring times of the patients as functions of all available clinical variables as potential predictors using CopBoostDepCens. We tested Weibull and log-normal marginals in combination with the Clayton, Gaussian and Gumbel copulas. To select the best model, we evaluated the predictive log-likelihood score based on 10-fold cross-validation (cf. Hans et al. 2023) (see SM C). The best stopping iteration $m_{\text {stop}}$ for each model was also determined based on 10-fold cross-validation.

#### Benchmarks

In line with the approach used in the simulation study, we compare CopBoostDepCens to a distributional AFT model, which does not account for potential dependent censoring but is also estimated via boosting (as in the simulations). Additionally, we include a boosted Cox model in the comparison, as it is the most traditional and widely utilized method for analyzing survival data. This evaluation serves as a sanity check rather than a competitive comparison. The R-code to reproduce all analyses can be found on GitHub https://github.com/AnnikaStr/CopBoostDepCens.

### Results

The best-performing combination of marginal distributions and copula with respect to predictive performance was the Weibull distribution with a Clayton copula. We hence also used the Weibull distribution for the AFT model.

Table 3 displays the estimated coefficients for CopBoostDepCens, the Cox and AFT models. One can observe at first glance that many of the clinical variables provided in the dataset are identified as relevant predictors for the survival of colon cancer patients by the boosting approaches. The resulting models are not particularly sparse. For example, for our copula model all variables are selected for the location parameter of the survival time $\mu _T$. This is also confirmed by the Cox and AFT models, where almost all variables have been selected for the hazards or the location parameter $\mu$, respectively. When interpreting the coefficients, one has to keep in mind that the copula and the AFT model rely on modelling directly the event time, while the Cox model focuses on the hazards as quantity of interest. As a result, for example the age of a patient has a negative effect on $\mu _T$ in the copula model and $\mu$ in the AFT model but a positive (multiplicative) effect for the Cox model. Both refer hence to a negative impact of age on the survival of patients with colon cancer. When just looking at the survival part of the copula model, one can again (cf., Section 3) observe the similarities of CopBoostDepCens and the AFT model when utilizing the same marginal distribution: the resulting estimated coefficients for $\mu _T$ and $\mu$ as well as $\sigma _T$ and $\sigma$ are overall very similar regarding magnitude and direction of effects.

The strength of CopBoostDepCens is to take the association ($\rho$) between the survival and the censoring time into account. Compared to the distribution of event times, less variables are relevant for modelling the distribution of censoring times and the association between event and censoring times. In particular for the dependence parameter, only two variables are selected: chemotherapy and tumor stage IV. This provides evidence for dependent censoring, influenced also by covariates. While chemotherapy is in general associated with longer survival times (positive effect on $\mu _{T}$) it has a strong negative effect on the association parameter, leading to a smaller association between survival time and censoring time. This can be interpreted as a broader potential range of survival time given that a patient is censored. A similar effect can be observed for the tumor staging, where stage IV is unfortunately associated with much shorter survival of the patient, but also has a negative effect on the association parameter.

**Fig. 4:**
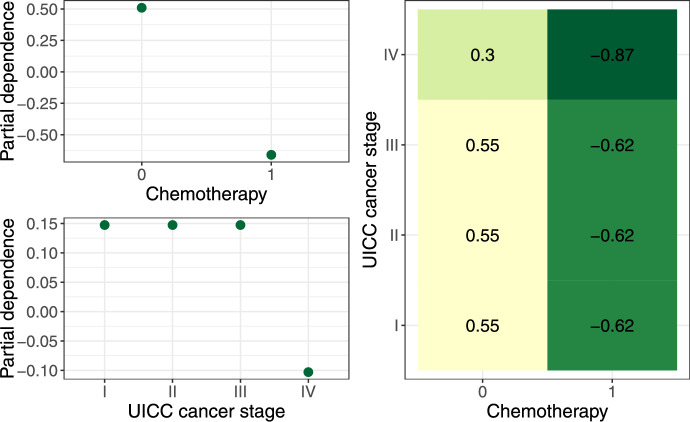
Partial effects on the copula dependence parameter ($\rho$), illustrating the effect of chemotherapy and UICC cancer stage on $\rho$. The top left plot shows the marginal effect of chemotherapy, while the bottom left plot shows the marginal effect of cancer stage. The right plot displays the joint partial dependence, illustrating the interaction between chemotherapy and cancer stage on $\rho$

Figure 4 displays the partial effects on the dependence parameter ($\rho$), showing how chemotherapy and UICC cancer stage influence $\rho$. The top left plot shows the marginal effect of chemotherapy, while the bottom left plot shows the marginal effect of cancer stage. When chemotherapy is not administered, the dependence parameter is around 0.5, indicating a positive association between survival and censoring time. When patients receive chemotherapy, $\rho$ decreases to approximately –0.7, closely aligning with the estimated coefficient of –1.17. Similarly, I - III of UICC cancer are associated with a dependence of about 0.15, while stage IV shows a decrease to approximately –0.1, consistent with the coefficient estimate of –0.25. The right plot displays the joint partial dependence, showing that the combination of chemotherapy and stage IV results in the lowest dependence values. This confirms that both factors, particularly chemotherapy, strongly reduce the association between survival and censoring time, in line with the coefficients in Table 3.

**Table 3:** Observational study

	**Copula model**					**AFT model**		** Cox model**
	$\mu _T$	$\sigma _T$	$\mu _C$	$\sigma _C$	$\rho$	$\mu$	$\sigma$	$\exp (\text {coef})$
Intercept	6.070	$-$0.270	3.061	$-$0.428	0.546	6.734	$-$0.329	0.094
Age	$-$0.016	$-$0.001	0.007	0.002	–	$-$0.019	–	1.021
Sex, male	$-$0.033	0.071	0.006	$-$0.081	–	$-$0.017	0.118	–
Chemotherapy	0.540	0.592	0.117	0.186	$-$1.170	0.334	0.752	0.500
ASA score, severe	$-$0.693	$-$0.195	–	$-$0.143	–	$-$0.728	$-$0.242	3.259
UICC cancer stage II	$-$0.092	–	–	0.029	–	–	–	1.373
stage III	$-$0.393	0.052	0.111	0.231	–	$-$0.513	–	2.100
stage IV	$-$1.081	$-$0.147	–	–	$-$0.250	$-$1.349	$-$0.228	3.175
LNE	0.009	0.008	0.007	0.011	–	0.006	0.007	0.981
LNR	$-$1.616	0.434	0.560	–	–	$-$1.679	0.387	3.702
R status	$-$0.153	$-$0.014	0.220	0.519	–	$-$0.063	$-$0.033	2.424
Preexisting cancer	$-$0.398	$-$0.020	$-$0.060	$-$0.014	–	$-$0.462	–	1.524

Estimated coefficients for all clinical variables with CopBoostDepCens, the AFT model and the Cox model

## Discussion

We developed CopBoostDepCens, a model-based boosting approach that relies on parametric marginal distributions and copulas, ensuring model identification. CopBoostDepCens does not assume a known copula and allows to include large numbers of potential predictor variables.

Modelling both survival and censoring times together through a copula in combination with a component-wise boosting approach bridges the gap from a compelling theoretical idea to a practical and versatile modelling option in many disciplines.

We demonstrated through simulations that CopBoostDepCens is able to identify the correct predictors in various data situations, including varying numbers of potential covariates and different censoring rates. While boosting the distributional AFT model (assuming independence) also may yield satisfying results, particularly in situations with a low censoring rate or a weak dependence between survival and censoring times, CopBoostDepCens provides additional insights into the dependence between survival and censoring times, not captured by classical approaches. For instance, in our analysis of the overall survival of colon cancer patients—a scenario commonly encountered by biostatisticians—we identified, among other findings, a negative effect of chemotherapy on the relationship between survival and censoring. This insight could not be obtained using previous models.

Evaluating the predictive performance of our model and comparing it with standard approaches such as the AFT model, is challenging. Many conventional metrics, such as the mean absolute error or concordance index, either exclude censored observations or assume non-informative censoring, which leads to biased results or misleading conclusions (Qi et al. 2023). Even metrics that attempt to account for censoring, such as the Brier score, pose challenges. Traditional implementations estimate the censoring distribution using Kaplan–Meier estimator, which assumes independence between survival and censoring times. In contrast, CopBoostDepCens explicitly models the censoring distribution as function of covariates. This fundamental difference complicates the comparison between models and raises concerns about the appropriateness of using standard evaluation metrics that assume independent censoring.

These limitations underscore the need for developing metrics that adequately capture the model performance under dependent censoring (Foomani et al. 2023). Recent work has started to close this gap: Prince et al. (2025) demonstrates that the Brier score is sensitive to the choice of weighting and provides guidance on inverse probability weighting (IPCW), while Lillelund et al. (2025) demonstrates the bias of traditional metrics and propose a copula-based alternative that uses a known Archimedean copula to estimate ${\hat{G}}(t)$. However, this approach requires a known copula structure and ignores covariates. Our method avoids these constraints: rather than relying on a copula-graphic estimator, our model directly estimates the censoring distribution conditional on covariates and allows the dependency parameter to vary with covariate effects.

An approach related to ours is that of Midtfjord et al. (2022). The authors employ the Clayton copula to account for dependent censoring. However, their model is restricted to the Clayton copula and limited to a fixed dependence parameter. Furthermore, it assumes that censoring is independent of covariates. In contrast, our boosting approach flexibly models all distributional parameters as functions of covariates.

This flexibility and the ability to model each parameter of the margins and the copula based on covariates also comes with the challenge of interpreting the effects of covariates involved in multiple parameters. Further research is warranted to reduce model complexity, for example, by deselecting covariates that have only a minor impact on overall model performance (Strömer et al. 2022, 2025). An inherent limitation of CopBoostDepCens is the difficulty in providing standard errors for the resulting coefficients, which is true for all boosting approaches. Due to early stopping and the resulting shrinkage of effect estimates, there are no closed formulas for standard errors. To address this, permutation tests could be utilized for significance testing and to provide *p*-values (Mayr et al. 2017; Hepp et al. 2019), though this would further increase computational costs. Another limitation of our boosting approach is that we currently only have empirical evidence from the simulation study and the application on its performance, but no formal theoretical proof regarding the consistency of effect estimates or asymptotics. Together with the missing standard errors, this makes statistical boosting in general most suitable for exploratory data analyses or prediction modelling—not for confirmatory data analyses (Mayr and Hofner 2018; Strömer et al. 2023).

Lastly, our conditional censoring approach is naturally limited to settings with a sufficient number of censored and uncensored individuals. In our simulations, we considered different censoring rates ranging from 20% to 80%. Also it is clear, that for a complex model as ours where we relate all distribution parameters from two marginal distributions as well as a copula parameter to covariates, one needs a reasonable large sample size for reliable estimation and to detect the most informative predictors. In our simulations, we used $n = 1000$ for most settings, a setting with $n = 100$ can be found in the Supplementary Material. This limitation is already true for most distributional regression approaches, but for CopBoostDepCens becomes even more pressing, as only parts of the observations can be used for estimating the corresponding marginal distributions.

While our current implementation uses one-parameter copulas, the modular structure of the boosting algorithm would allow the integration of more advanced copula families. These could include, for example, Vine (Czado and Nagler 2022) and mixture copulas (Pan et al. 2025) to better capture asymmetric or time-varying dependencies. However, careful consideration of pair-copula selection, identifiability constraints and sample size requirements is necessary for these extensions, particularly in smaller studies (Barthel et al. 2019). Another potential topic for future research involves extending the approach to accommodate left censored or truncated data (Deresa et al. 2022). Doing so could broaden the applicability of the model to a wider range of survival analysis scenarios. Building on recent advances by Deresa and Van Keilegom (2024a) and Deresa and Van Keilegom (2024b), future work could incorporate semi-parametric margins (e.g., a Cox proportional hazards model for survival times with nonparametric baseline hazards) to relax the parametric assumptions while preserving identifiability under dependent censoring.

## Supplementary Materials

Tables and figures referenced in Sects. 3 and 4 are available with this paper online. The code is available at https://github.com/AnnikaStr/CopBoostDepCens.

## Supplementary Information

Below is the link to the electronic supplementary material.

Supplementary file 1 (pdf 10050 KB)
