# Supplementary material for: Modelling dependent censoring in time-to-event data using boosting copula regression
Source: Lifetime Data Anal. 2025 Oct 21;31(4):994–1016. doi: 10.1007/s10985-025-09674-x (PMC12586418; doi:10.1007/s10985-025-09674-x)
Supplement: Supplementary file 1 — (pdf 10050 KB) [file 10985_2025_9674_MOESM1_ESM.pdf]

Supplementary Materials - Modelling dependent censoring in time-to-event data by boosting copula regression

This Supplementary Material contains the following sections.

A Simulation results with Weibull marginal distributions

B Simulation results with log-normal marginal distributions

C Comparison of intercept models with Czado and Van Keilegom (2023)

D Model selection results for the application on survival of colon cancer patients

Address(es) of author(s) should be given

Overview of simulation settings

Setting	Copula	Margins	Kendall's τ	n	p^*
**1**: positive dependence	Clayton<br>Gumbel<br>Gaussian	Weibull<br>Log-normal	$[0.45; 0.94]$<br>$[0.31; 0.96]$<br>$[0.39; 0.97]$	1000<br>100	$p^* \in \{50, 250, 500, 1000, 2500\}$<br>$p^* = 1000$
**2**: weaker/negative dependence	Gaussian	Weibull	$[-0.43; 0.63]$	1000	$p^* \in \{50, 250, 500, 1000\}$
**3**: independent censoring	Clayton<br>Gumbel<br>Gaussian	Weibull<br>Log-normal	$\tau = 0$	1000	$p^* \in \{50, 250, 500, 1000\}$

A Simulation results with Weibull marginal distributions

A.1 Gaussian Copula

A.1.1 Setting 1

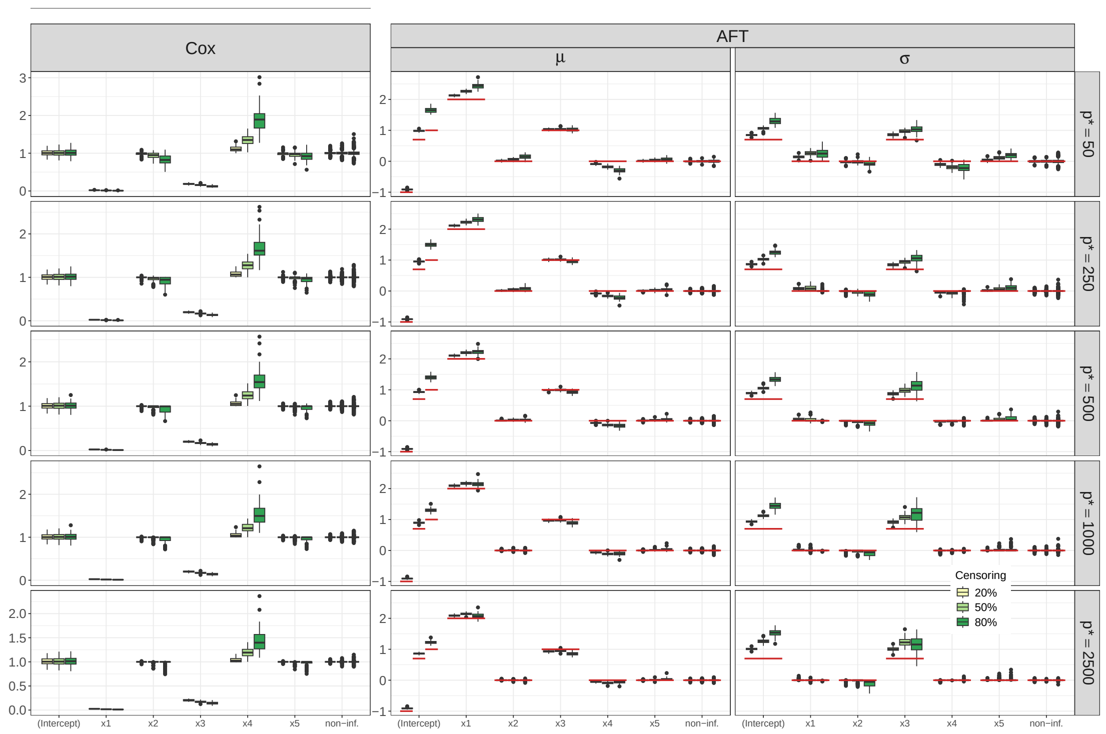

Fig. A1 Setting 1: Boxplot of estimated coefficients of the Cox and AFT models on the 100 replicates of the Gauss copula with Weibull distributed margins for different numbers of noise variables. The box colors represent the average proportions of censoring. The red horizontal lines show the true values for each.

	Censoring	Brier score Cox	Integrated Brier score Copula	Cox	AFT
p* = 50	20%	0.45 (0.09)	0.07 (0.01)	0.15 (0.18)	0.07 (0.11)
	50%	0.41 (0.12)	0.07 (0.05)	0.16 (0.21)	0.14 (0.21)
	80%	0.23 (0.14)	0.07 (0.05)	0.17 (0.11)	0.13 (0.20)
p* = 250	20%	0.46 (0.10)	0.07 (0.01)	0.15 (0.19)	0.10 (0.17)
	50%	0.41 (0.11)	0.09 (0.06)	0.14 (0.21)	0.10 (0.16)
	80%	0.25 (0.16)	0.08 (0.04)	0.17 (0.14)	0.13 (0.21)
p* = 500	20%	0.46 (0.08)	0.07 (0.01)	0.14 (0.17)	0.08 (0.13)
	50%	0.42 (0.11)	0.10 (0.07)	0.16 (0.23)	0.13 (0.20)
	80%	0.22 (0.10)	0.08 (0.05)	0.16 (0.13)	0.10 (0.12)
p* = 1000	20%	0.46 (0.09)	0.07 (0.00)	0.13 (0.16)	0.09 (0.15)
	50%	0.43 (0.14)	0.11 (0.06)	0.15 (0.21)	0.12 (0.19)
	80%	0.23 (0.13)	0.08 (0.04)	0.17 (0.16)	0.12 (0.19)
p* = 2500	20%	0.47 (0.11)	0.06 (0.00)	0.14 (0.17)	0.09 (0.15)
	50%	0.44 (0.14)	0.13 (0.05)	0.17 (0.26)	0.13 (0.21)
	80%	0.24 (0.12)	0.07 (0.05)	0.17 (0.16)	0.12 (0.17)

Table A1 Setting 1: Mean (SDs) of the Brier score and integrated Brier score for CopBoostDepCens, the Cox and the AFT models on the 100 replicates of the Gaussian copula with Weibull-distributed margins for different numbers of noise variables.

		IAE	Integrated squared error			
		Survival time	Survival time			Censoring time
	Censoring	Cox	Copula	Cox	AFT	Copula
p* = 50	20%	1.50 (0.23)	0.21 (0.02)	0.91 (0.17)	0.02 (0.01)	0.20 (0.03)
	50%	7.36 (1.76)	0.19 (0.01)	4.68 (1.30)	0.77 (0.17)	0.46 (0.03)
	80%	6.28 (2.52)	0.25 (0.06)	3.99 (1.93)	2.25 (0.77)	0.21 (0.02)
p* = 250	20%	1.47 (0.21)	0.21 (0.02)	0.87 (0.15)	0.02 (0.01)	0.18 (0.03)
	50%	6.91 (1.31)	0.20 (0.02)	4.26 (0.95)	0.58 (0.14)	0.43 (0.02)
	80%	5.96 (2.76)	0.31 (0.08)	3.68 (2.12)	1.40 (0.55)	0.20 (0.02)
p* = 500	20%	1.42 (0.20)	0.20 (0.02)	0.83 (0.13)	0.02 (0.01)	0.18 (0.03)
	50%	7.12 (1.45)	0.23 (0.03)	4.38 (1.03)	0.48 (0.12)	0.44 (0.02)
	80%	5.54 (1.98)	0.32 (0.09)	3.32 (1.46)	1.00 (0.36)	0.20 (0.02)
p* = 1000	20%	1.44 (0.17)	0.20 (0.01)	0.84 (0.12)	0.02 (0.01)	0.21 (0.04)
	50%	7.16 (1.58)	0.26 (0.04)	4.40 (1.13)	0.41 (0.11)	0.45 (0.03)
	80%	6.10 (2.55)	0.34 (0.09)	3.72 (1.91)	0.75 (0.33)	0.20 (0.02)
p* = 2500	20%	1.43 (0.21)	0.19 (0.02)	0.83 (0.14)	0.02 (0.01)	0.30 (0.05)
	50%	0.47 (0.11)	0.31 (0.05)	4.48 (1.27)	0.36 (0.09)	0.47 (0.03)
	80%	6.15 (3.09)	0.36 (0.10)	3.75 (2.33)	0.55 (0.24)	0.19 (0.02)

Table A2 Setting 1: Means (SDs) of the integrated absolute (IAE) and integrated squared error (ISE) for CopBoostDepCens, the Cox and the AFT models on the 100 replicates of the Gaussian copula with Weibull-distributed margins for different numbers of noise variables.

	Censoring	Copula	Cox	AFT
p* = 50	20%	104.61 (23.27)	1.30 (1.04)	1.38 (0.97)
	50%	77.31 (19.23)	1.07 (0.66)	2.65 (1.11)
	80%	93.48 (23.48)	2.38 (0.72)	3.70 (1.14)
p* = 250	20%	51.33 (12.38)	0.79 (0.14)	0.86 (0.20)
	50%	53.69 (10.51)	0.98 (0.49)	1.61 (0.60)
	80%	53.23 (10.49)	1.71 (0.54)	2.73 (0.50)
p* = 500	20%	50.92 (8.72)	1.00 (0.35)	1.11 (0.49)
	50%	46.84 (9.13)	1.04 (0.45)	1.63 (0.56)
	80%	46.18 (8.37)	2.10 (0.84)	2.73 (0.73)
p* = 1000	20%	41.43 (12.56)	1.02 (0.40)	1.15 (0.53)
	50%	42.10 (7.91)	1.04 (0.51)	1.92 (0.66)
	80%	54.89 (8.81)	1.70 (0.69)	3.07 (0.57)
p* = 2500	20%	33.96 (2.68)	0.79 (0.09)	0.85 (0.07)
	50%	39.63 (5.42)	0.94 (0.07)	1.03 (0.20)
	80%	38.09 (4.56)	1.26 (0.46)	1.48 (0.48)

Table A3 Setting 1: Means (SDs) of the runtime for CopBoostDepCens, the Cox and AFT models of the Gaussian copula with Weibull distributed margins on the 100 replicates.

	Parameter	Censoring 20% (Int)	x_1	x_2	x_3	x_4	x_5	non-inf.	Censoring 50% (Int)	x_1	x_2	x_3	x_4	x_5	non-inf.	Censoring 80% (Int)	x_1	x_2	x_3	x_4	x_5	non-inf.
p* = 50	μ_T	100	100	87	100	100	86	81.6	100	100	76	100	86	87	78.4	100	100	81	100	74	87	63
	σ_T	100	66	56	100	89	77	65.6	100	100	50	100	49	66	58.4	100	96	54	100	39	51	45.8
	μ_C	100	60	100	53	100	39	45.4	100	59	100	50	100	48	55.2	100	48	100	59	100	60	52.6
	σ_C	100	66	100	51	57	61	45.8	100	77	100	49	55	56	59.6	100	62	100	61	65	62	60
	ρ	100	100	7	6	19	100	5	100	100	7	5	6	100	4.8	100	29	33	12	100	100	3.4
p* = 250	μ_T	100	100	63	100	100	60	49.2	100	100	41	100	64	72	43.2	100	100	57	100	38	69	21.0
	σ_T	100	25	20	100	70	51	26.2	100	98	9	100	22	36	16.8	100	96	18	100	6	36	9.6
	μ_C	100	21	100	6	100	4	5.2	100	42	100	10	100	12	11.8	100	14	100	15	100	15	15.0
	σ_C	100	5	100	13	30	10	6.8	100	51	100	20	12	21	20.2	100	25	100	33	23	23	24.0
	ρ	100	100	0	0	1	82	0.0	100	100	0	0	0	100	0.0	100	9	17	0	100	88	0.0
p* = 500	μ_T	100	100	62	100	100	50	36.0	100	100	25	100	59	67	28.2	100	100	38	100	17	62	14.4
	σ_T	100	11	14	100	64	39	12.6	100	98	5	100	9	35	6.8	100	95	19	100	4	33	4.4
	μ_C	100	6	100	1	100	1	1.4	100	34	100	3	100	4	4.8	100	4	100	6	100	9	8.0
	σ_C	100	1	100	6	26	3	1.0	100	38	100	12	5	9	10.2	100	14	100	28	15	13	16.2
	ρ	100	100	0	0	1	82	0.0	100	100	0	0	0	100	0.0	100	9	17	0	100	88	0.0
p* = 1000	μ_T	100	100	51	100	100	28	22.0	100	100	12	100	57	59	16.8	100	100	25	100	7	59	8.8
	σ_T	100	8	11	100	42	20	3.8	100	98	0	100	2	29	2.2	100	94	15	100	3	28	2.0
	μ_C	100	0	100	0	100	0	0.0	100	30	100	0	100	1	1.2	100	2	100	5	100	8	5.0
	σ_C	100	0	99	3	24	1	0.0	100	20	100	10	2	4	4.8	100	7	100	22	10	5	11.4
	ρ	100	100	0	0	0	1	0.0	100	100	0	0	0	85	0.0	100	8	17	0	100	34	0.0
p* = 2500	μ_T	100	100	24	100	100	5	5.2	100	100	4	100	48	51	8.2	100	100	10	100	0	48	3.6
	σ_T	100	4	3	100	3	1	0.0	100	98	0	100	1	21	0.6	100	91	10	100	1	17	0.4
	μ_C	100	0	90	0	100	0	0.0	100	22	100	0	100	0	0.4	100	1	100	3	100	7	3.0
	σ_C	100	0	63	1	16	0	0.0	100	15	100	6	1	1	1.6	100	4	100	18	7	4	5.4
	ρ	100	100	0	0	0	0	0.0	100	100	0	0	0	31	0.0	100	8	17	0	100	2	0.0

Table A4 Setting 1: Selection rates for CopBoostDepCens of the Gaussian copula with Weibull distributed margins on the 100 replicates.

Special Case: Results for $n = 100$ and $p^* = 1000$

	Censoring	Brier score Copula	Brier score Cox	Brier score AFT	Integrated Brier score Copula	Integrated Brier score Cox	Integrated Brier score AFT
$p^* = 1000$	20%	0.12 (0.03)	0.31 (0.21)	0.17 (0.25)	0.12 (0.03)	0.20 (0.28)	0.16 (0.27)
	50%	0.13 (0.05)	0.35 (0.27)	0.25 (0.29)	0.21 (0.09)	0.28 (0.35)	0.28 (0.32)
	80%	0.07 (0.04)	0.25 (0.20)	0.21 (0.21)	0.12 (0.06)	0.28 (0.30)	0.33 (0.31)

Table A5 Special case: Mean (SD) of the Brier score and integrated Brier score for CopBoostDepCens, the Cox and the AFT models on the 100 replicates of the Gaussian copula with Weibull-distributed margins, with sample size $n = 100$ and $p^* = 1000$ covariates.

		Integrated absolute error				Integrated squared error			
		Survival time			Censoring time	Survival time			Censoring time
	Censoring	Copula	Cox	AFT	Copula	Copula	Cox	AFT	Copula
$p^* = 1000$	20%	0.81 (0.10)	1.20 (0.28)	0.12 (0.04)	1.41 (0.19)	0.17 (0.02)	0.65 (0.19)	0.02 (0.01)	0.57 (0.08)
	50%	1.75 (0.46)	5.39 (1.87)	1.02 (0.34)	2.13 (0.26)	0.70 (0.33)	3.02 (1.34)	0.29 (0.18)	0.62 (0.11)
	80%	2.42 (0.67)	3.77 (1.81)	2.14 (0.64)	0.94 (0.12)	1.36 (0.53)	1.87 (1.20)	1.02 (0.48)	0.14 (0.02)

Table A6 Special case: Means (SDs) of the integrated absolute and integrated squared error for CopBoostDepCens, the Cox and the AFT models on the 100 replicates of the Gaussian copula with Weibull-distributed margins, with sample size $n = 100$ and $p^* = 1000$ covariates.

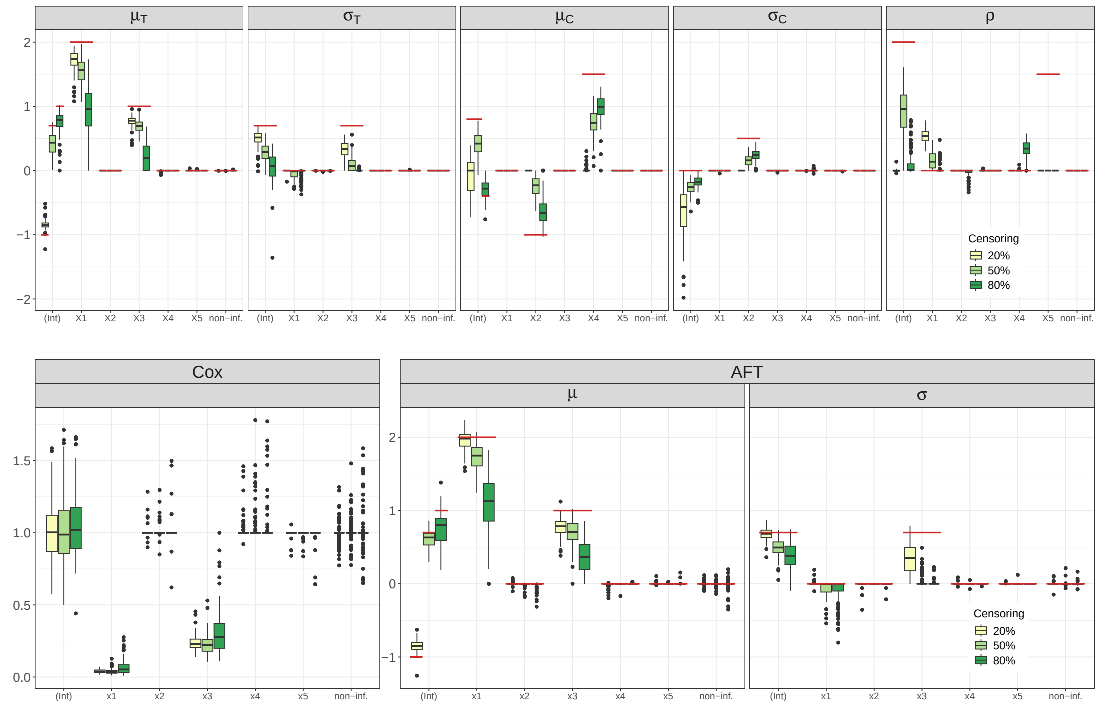

Fig. A2 Special case: Boxplot of estimated coefficients of CopBoostDepCens, the AFT and the Cox model on the 100 replicates. Results are shown for the specific setting with sample size $n = 100$ and $p^* = 1000$ covariates of the Gaussian copula with Weibull-distributed margins. The box colors represent the average proportions of censoring. The red horizontal lines show the true values for each.

	Censoring	Copula	Cox	AFT
$p^* = 1000$	20%	3.12 (0.66)	0.07 (0.00)	0.6 (0.06)
	50%	4.55 (1.28)	0.09 (0.02)	0.84 (0.24)
	80%	4.61 (1.20)	0.08 (0.03)	1.24 (0.62)

Table A7 Special case: Means (SDs) of the runtime for CopBoostDepCens, the Cox and AFT models of the Gaussian copula with Weibull distributed margins, with sample size $n = 100$ and $p^* = 1000$ covariates on the 100 replicates.

A.1.2 Setting 2

		Brier score			Integrated Brier score		
	Censoring	Copula	Cox	AFT	Copula	Cox	AFT
p* = 50	20%	0.07 (0.00)	0.47 (0.08)	0.10 (0.10)	0.07 (0.00)	0.15 (0.15)	0.08 (0.08)
	50%	0.08 (0.01)	0.45 (0.11)	0.15 (0.18)	0.08 (0.04)	0.17 (0.22)	0.16 (0.22)
	80%	0.05 (0.01)	0.32 (0.14)	0.16 (0.19)	0.07 (0.03)	0.20 (0.25)	0.21 (0.28)
p* = 250	20%	0.07 (0.00)	0.46 (0.06)	0.10 (0.11)	0.07 (0.00)	0.14 (0.16)	0.09 (0.14)
	50%	0.08 (0.01)	0.45 (0.11)	0.14 (0.17)	0.09 (0.04)	0.14 (0.19)	0.12 (0.17)
	80%	0.05 (0.02)	0.31 (0.14)	0.14 (0.18)	0.07 (0.03)	0.17 (0.21)	0.16 (0.22)
p* = 500	20%	0.08 (0.00)	0.46 (0.04)	0.09 (0.06)	0.07 (0.00)	0.13 (0.12)	0.08 (0.07)
	50%	0.08 (0.01)	0.45 (0.10)	0.15 (0.16)	0.10 (0.04)	0.18 (0.24)	0.15 (0.20)
	80%	0.05 (0.01)	0.30 (0.11)	0.14 (0.15)	0.07 (0.02)	0.20 (0.24)	0.18 (0.23)
p* = 1000	20%	0.08 (0.00)	0.46 (0.06)	0.10 (0.09)	0.07 (0.00)	0.13 (0.13)	0.09 (0.12)
	50%	0.08 (0.01)	0.46 (0.10)	0.15 (0.18)	0.10 (0.03)	0.19 (0.26)	0.15 (0.21)
	80%	0.05 (0.01)	0.29 (0.09)	0.13 (0.11)	0.06 (0.02)	0.18 (0.23)	0.16 (0.20)

Table A8 Setting 2: Mean (SD) of the Brier score and integrated Brier score for CopBoostDepCens, the Cox and the AFT models on the 100 replicates of the Gaussian copula with Weibull-distributed margins for different numbers of noise variables.

		Integrated absolute error				Integrated squared error			
		Survival time			Censoring time	Survival time			Censoring time
	Censoring	Copula	Cox	AFT	Copula	Copula	Cox	AFT	Copula
p* = 50	20%	0.91 (0.09)	1.35 (0.22)	0.06 (0.02)	0.83 (0.12)	0.20 (0.02)	0.77 (0.15)	0.01 (0.00)	0.18 (0.04)
	50%	1.19 (0.10)	5.85 (1.52)	0.58 (0.17)	1.97 (0.14)	0.23 (0.04)	3.29 (1.03)	0.09 (0.05)	0.44 (0.03)
	80%	1.20 (0.18)	4.51 (1.96)	0.88 (0.32)	1.33 (0.18)	0.25 (0.06)	2.34 (1.30)	0.18 (0.10)	0.20 (0.02)
p* = 250	20%	0.90 (0.08)	1.31 (0.19)	0.07 (0.01)	0.81 (0.11)	0.20 (0.02)	0.73 (0.13)	0.01 (0.00)	0.18 (0.03)
	50%	1.24 (0.11)	5.48 (1.25)	0.51 (0.10)	1.94 (0.13)	0.27 (0.06)	2.98 (0.83)	0.07 (0.03)	0.43 (0.02)
	80%	1.21 (0.19)	4.16 (1.79)	0.73 (0.17)	1.29 (0.16)	0.27 (0.07)	2.05 (1.14)	0.13 (0.05)	0.19 (0.02)
p* = 500	20%	0.89 (0.08)	1.27 (0.19)	0.07 (0.01)	0.83 (0.13)	0.19 (0.02)	0.70 (0.13)	0.01 (0.00)	0.20 (0.05)
	50%	1.28 (0.12)	5.64 (1.28)	0.49 (0.08)	1.98 (0.13)	0.29 (0.07)	3.05 (0.85)	0.07 (0.02)	0.44 (0.03)
	80%	1.23 (0.18)	4.09 (1.61)	0.74 (0.14)	1.28 (0.15)	0.29 (0.08)	1.98 (1.01)	0.15 (0.05)	0.19 (0.02)
p* = 1000	20%	0.90 (0.07)	1.29 (0.16)	0.08 (0.01)	0.90 (0.12)	0.19 (0.01)	0.70 (0.11)	0.01 (0.00)	0.25 (0.05)
	50%	1.33 (0.14)	5.76 (1.35)	0.50 (0.07)	1.99 (0.14)	0.32 (0.08)	3.10 (0.88)	0.07 (0.02)	0.45 (0.03)
	80%	1.28 (0.23)	4.38 (2.01)	0.83 (0.18)	1.30 (0.18)	0.32 (0.11)	2.14 (1.26)	0.19 (0.07)	0.19 (0.02)

Table A9 Setting 2: Means (SDs) of the integrated absolute and integrated squared error for CopBoostDepCens, the Cox and the AFT models on the 100 replicates of the Gaussian copula with Weibull-distributed margins for different numbers of noise variables.

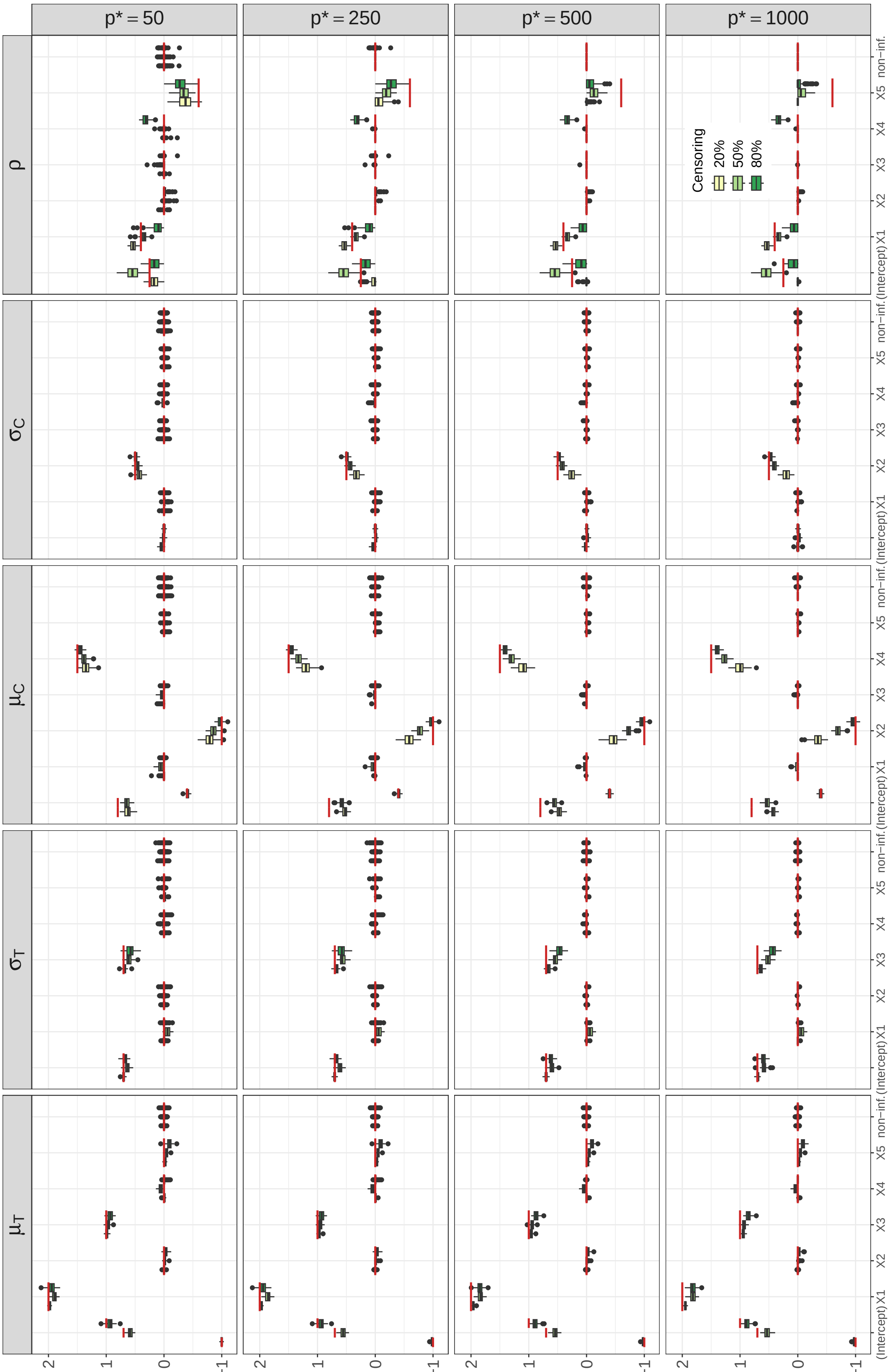

Fig. A3 Setting 2: Boxplot of estimated coefficients of CopBoostDepCens on the 100 replicates. Results are shown for each distribution parameter (columns) of the Gaussian copula with Weibull-distributed margins for different numbers of noise variables. The box colors represent the average proportions of censoring. The red horizontal lines show the true values for each.

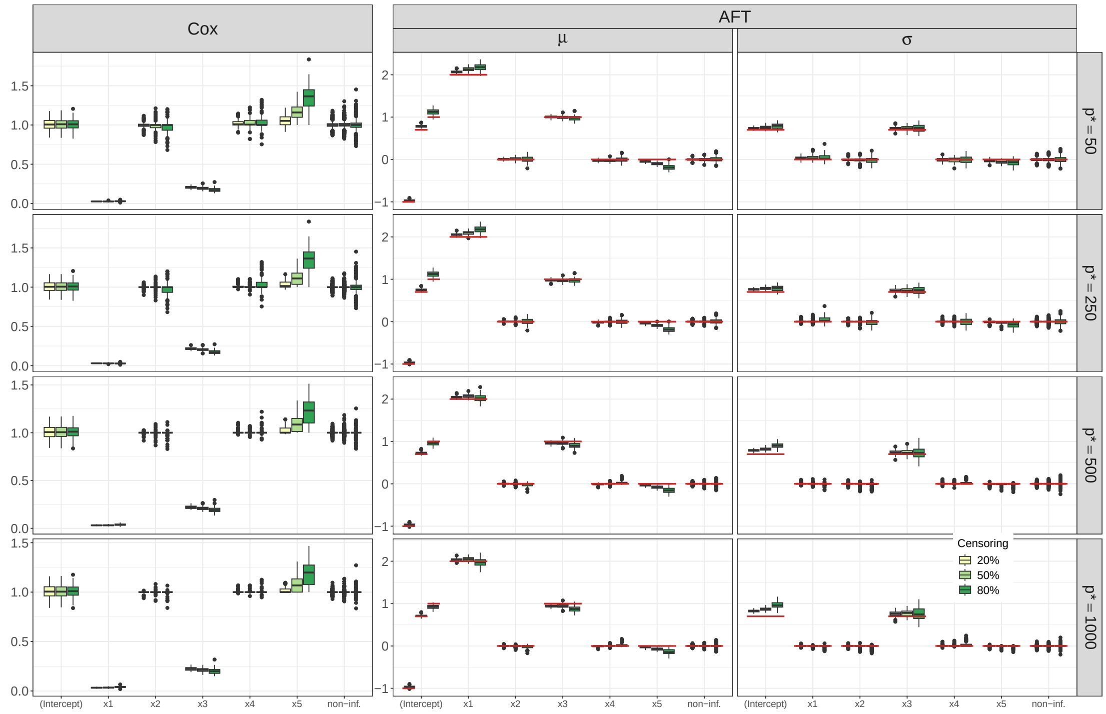

Fig. A4 Setting 2: Boxplot of estimated coefficients of the Cox and AFT models on the 100 replicates. Results are shown for each distribution parameter (columns) of the Gaussian copula with Weibull-distributed margins for different numbers of noise variables. The box colors represent the average proportions of censoring. The red horizontal lines show the true values for each.

	Censoring	Copula	Cox	AFT
$p^* = 50$	20%	43.08 (10.23)	0.88 (0.55)	0.63 (0.26)
	50%	36.25 (10.08)	0.83 (0.51)	1.19 (0.45)
	80%	40.02 (10.00)	1.13 (0.57)	1.72 (0.35)
$p^* = 250$	20%	35.85 (6.33)	0.86 (0.34)	0.74 (0.34)
	50%	44.44 (6.72)	0.89 (0.33)	1.57 (0.53)
	80%	49.27 (10.30)	1.28 (0.80)	2.58 (0.64)
$p^* = 500$	20%	25.34 (5.55)	0.77 (0.24)	0.68 (0.26)
	50%	30.11 (5.47)	0.75 (0.28)	1.23 (0.44)
	80%	36.51 (6.87)	0.97 (0.57)	1.94 (0.50)
$p^* = 1000$	20%	25.25 (5.00)	0.79 (0.24)	0.84 (0.36)
	50%	31.73 (6.46)	0.83 (0.33)	1.45 (0.54)
	80%	38.63 (7.30)	0.95 (0.59)	2.36 (0.66)

Table A10 Setting 2: Means (SDs) of the runtime for CopBoostDepCens, the Cox and AFT models of the Gaussian copula with Weibull distributed margins on the 100 replicates.

	Parameter	Censoring 20% (Int)	x_1	x_2	x_3	x_4	x_5	non-inf.	Censoring 50% (Int)	x_1	x_2	x_3	x_4	x_5	non-inf.	Censoring 80% (Int)	x_1	x_2	x_3	x_4	x_5	non-inf.
$p^* = 50$	μ_T	100	100	75	100	86	99	73.4	100	100	66	100	91	95	63.2	100	100	74	100	38	96	44.2
	σ_T	100	55	61	100	56	62	54.8	100	85	44	100	59	38	43.6	100	27	37	100	36	33	30.4
	μ_C	100	28	100	33	100	32	30.4	100	88	100	75	100	43	40.6	100	45	100	40	100	50	46.4
	σ_C	100	40	100	45	62	38	43.8	100	45	100	43	41	50	45.8	100	52	100	56	47	55	56.8
	ρ	100	100	8	6	6	100	7.0	100	100	18	27	18	100	9.8	100	89	42	6	100	99	5.8
$p^* = 250$	μ_T	100	100	45	100	61	95	40.0	100	100	46	100	86	94	33.0	100	100	74	100	38	96	44.2
	σ_T	100	16	23	100	23	33	18.4	100	82	14	100	24	5	12.8	100	27	37	100	36	33	30.4
	μ_C	100	3	100	2	100	12	3.2	100	71	100	48	100	11	9.8	100	45	100	40	100	50	46.4
	σ_C	100	5	100	11	40	14	7.2	100	24	100	16	10	10	14.4	100	52	100	56	47	55	56.8
	ρ	100	100	0	0	0	69	0.0	100	100	2	4	2	97	0.0	100	89	42	6	100	99	5.8
$p^* = 500$	μ_T	100	100	27	100	47	97	27.0	100	100	41	100	85	94	21.0	100	100	61	100	2	95	6.8
	σ_T	100	4	9	100	10	18	8.0	100	82	7	100	16	3	7.4	100	8	5	100	4	3	2.6
	μ_C	100	1	100	1	100	4	0.4	100	63	100	31	100	6	3.8	100	4	100	10	100	11	9.4
	σ_C	100	2	100	3	30	7	1.0	100	18	100	9	4	6	7.2	100	17	100	12	11	15	15.8
	ρ	100	100	0	0	0	21	0.0	100	100	2	1	1	87	0.0	100	74	12	0	100	65	0.0
$p^* = 1000$	μ_T	100	100	21	100	38	89	17.4	100	100	32	100	83	95	13.0	100	100	55	100	0	93	3.4
	σ_T	100	1	3	100	7	11	3.2	100	82	1	100	7	3	2.2	100	6	1	100	2	2	0.8
	μ_C	100	0	100	0	100	1	0.0	100	48	100	17	100	3	1.2	100	0	100	6	100	5	5.8
	σ_C	100	1	100	1	22	1	0.0	100	11	100	3	1	3	3.8	100	12	100	8	10	11	9.0
	ρ	100	100	0	0	0	0	0.0	100	100	1	1	1	76	0.0	100	74	9	0	100	43	0.0

Table A11 Setting 2: Selection rates for CopBoostDepCens of the Gaussian copula with Weibull distributed margins on the 100 replicates.

A.1.3 Setting 3

	Censoring	Brier score Copula	Brier score Cox	Brier score AFT	Integrated Brier score Copula	Integrated Brier score Cox	Integrated Brier score AFT
p* = 50	20%	0.07 (0.00)	0.47 (0.11)	0.12 (0.15)	0.07 (0.01)	0.14 (0.17)	0.10 (0.16)
	50%	0.08 (0.01)	0.47 (0.14)	0.17 (0.21)	0.09 (0.03)	0.18 (0.24)	0.16 (0.23)
	80%	0.05 (0.02)	0.30 (0.09)	0.11 (0.10)	0.07 (0.03)	0.15 (0.17)	0.14 (0.17)
p* = 250	20%	0.07 (0.00)	0.47 (0.08)	0.12 (0.14)	0.07 (0.01)	0.16 (0.20)	0.11 (0.18)
	50%	0.08 (0.01)	0.46 (0.12)	0.15 (0.17)	0.10 (0.03)	0.17 (0.23)	0.14 (0.2)
	80%	0.05 (0.01)	0.31 (0.14)	0.15 (0.17)	0.08 (0.03)	0.20 (0.25)	0.19 (0.23)
p* = 500	20%	0.08 (0.00)	0.47 (0.08)	0.11 (0.13)	0.07 (0.00)	0.15 (0.18)	0.09 (0.13)
	50%	0.09 (0.01)	0.46 (0.11)	0.15 (0.16)	0.11 (0.03)	0.18 (0.24)	0.15 (0.19)
	80%	0.05 (0.01)	0.30 (0.10)	0.12 (0.12)	0.07 (0.02)	0.16 (0.21)	0.15 (0.18)
p* = 1000	20%	0.08 (0.00)	0.48 (0.09)	0.13 (0.16)	0.07 (0.01)	0.17 (0.21)	0.13 (0.22)
	50%	0.09 (0.01)	0.45 (0.10)	0.14 (0.15)	0.12 (0.05)	0.16 (0.20)	0.14 (0.19)
	80%	0.06 (0.01)	0.31 (0.12)	0.13 (0.14)	0.08 (0.04)	0.15 (0.16)	0.15 (0.18)

Table A12 Setting 3: Mean (SD) of the Brier score and integrated Brier score for CopBoostDepCens, the Cox and the AFT models on the 100 replicates of the independent setting for the Gaussian copula with Weibull-distributed margins for different numbers of noise variables.

		Integrated absolute error				Integrated squared error			
		Survival time			Censoring time	Survival time			Censoring time
	Censoring	Copula	Cox	AFT	Copula	Copula	Cox	AFT	Copula
p* =50	20%	0.88 (0.09)	1.20 (0.22)	0.04 (0.01)	0.81 (0.13)	0.20 (0.02)	0.66 (0.15)	0.00 (0.00)	0.18 (0.04)
	50%	1.42 (0.19)	4.48 (1.30)	0.25 (0.06)	1.99 (0.21)	0.37 (0.10)	2.27 (0.82)	0.02 (0.01)	0.46 (0.04)
	80%	1.18 (0.32)	2.70 (1.72)	0.39 (0.16)	1.26 (0.23)	0.30 (0.14)	1.14 (1.00)	0.04 (0.03)	0.21 (0.03)
p* = 250	20%	0.90 (0.11)	1.13 (0.21)	0.06 (0.01)	0.87 (0.17)	0.20 (0.02)	0.6 (0.13)	0.01 (0.00)	0.23 (0.06)
	50%	1.53 (0.24)	4.18 (1.60)	0.40 (0.08)	1.96 (0.24)	0.47 (0.13)	2.03 (0.99)	0.05 (0.02)	0.47 (0.06)
	80%	1.38 (0.35)	2.74 (1.63)	0.69 (0.24)	1.25 (0.22)	0.44 (0.18)	1.13 (0.95)	0.14 (0.09)	0.20 (0.03)
p* = 500	20%	0.90 (0.11)	1.14 (0.23)	0.07 (0.01)	0.93 (0.18)	0.20 (0.02)	0.60 (0.14)	0.01 (0.00)	0.27 (0.07)
	50%	1.61 (0.21)	4.12 (1.13)	0.48 (0.09)	1.99 (0.19)	0.52 (0.13)	1.98 (0.70)	0.07 (0.03)	0.49 (0.05)
	80%	1.41 (0.33)	2.60 (1.22)	0.82 (0.24)	1.25 (0.18)	0.47 (0.18)	1.03 (0.66)	0.20 (0.09)	0.20 (0.02)
p* = 1000	20%	0.91 (0.12)	1.15 (0.24)	0.08 (0.01)	1.01 (0.20)	0.20 (0.02)	0.61 (0.16)	0.01 (0.00)	0.33 (0.09)
	50%	1.66 (0.26)	4.30 (1.36)	0.54 (0.10)	2.03 (0.22)	0.55 (0.15)	2.09 (0.83)	0.10 (0.03)	0.51 (0.06)
	80%	1.52 (0.37)	2.72 (1.61)	0.98 (0.28)	1.24 (0.19)	0.55 (0.20)	1.10 (0.90)	0.28 (0.11)	0.20 (0.02)

Table A13 Setting3: Means (SDs) of the integrated absolute and integrated squared error for CopBoostDepCens, the Cox and the AFT models on the 100 replicates of the independent setting for the Gaussian copula with Weibull-distributed margins for different numbers of noise variables.

	Censoring	Copula	Cox	AFT
p* = 50	20%	35.93 (13.09)	0.82 (0.43)	0.82 (0.17)
	50%	33.97 (15.88)	0.75 (0.40)	1.33 (0.25)
	80%	35.73 (9.68)	0.91 (0.52)	2.51 (0.57)
p* = 250	20%	24.31 (4.05)	0.71 (0.03)	0.81 (0.06)
	50%	23.68 (3.54)	0.63 (0.03)	1.23 (0.15)
	80%	29.02 (3.49)	0.72 (0.30)	2.14 (0.22)
p* = 500	20%	21.46 (3.83)	0.71 (0.02)	0.83 (0.07)
	50%	22.55 (3.80)	0.64 (0.03)	1.23 (0.21)
	80%	27.20 (2.66)	0.67 (0.24)	2.01 (0.29)
p* = 1000	20%	16.68 (2.54)	0.53 (0.02)	0.80 (0.06)
	50%	18.63 (2.97)	0.50 (0.06)	1.12 (0.19)
	80%	23.59 (2.26)	0.56 (0.22)	1.87 (0.34)

Table A14 Setting 3: Means (SDs) of the runtime for CopBoostDepCens, the Cox and AFT models of the Gaussian copula with Weibull distributed margins on the 100 replicates.

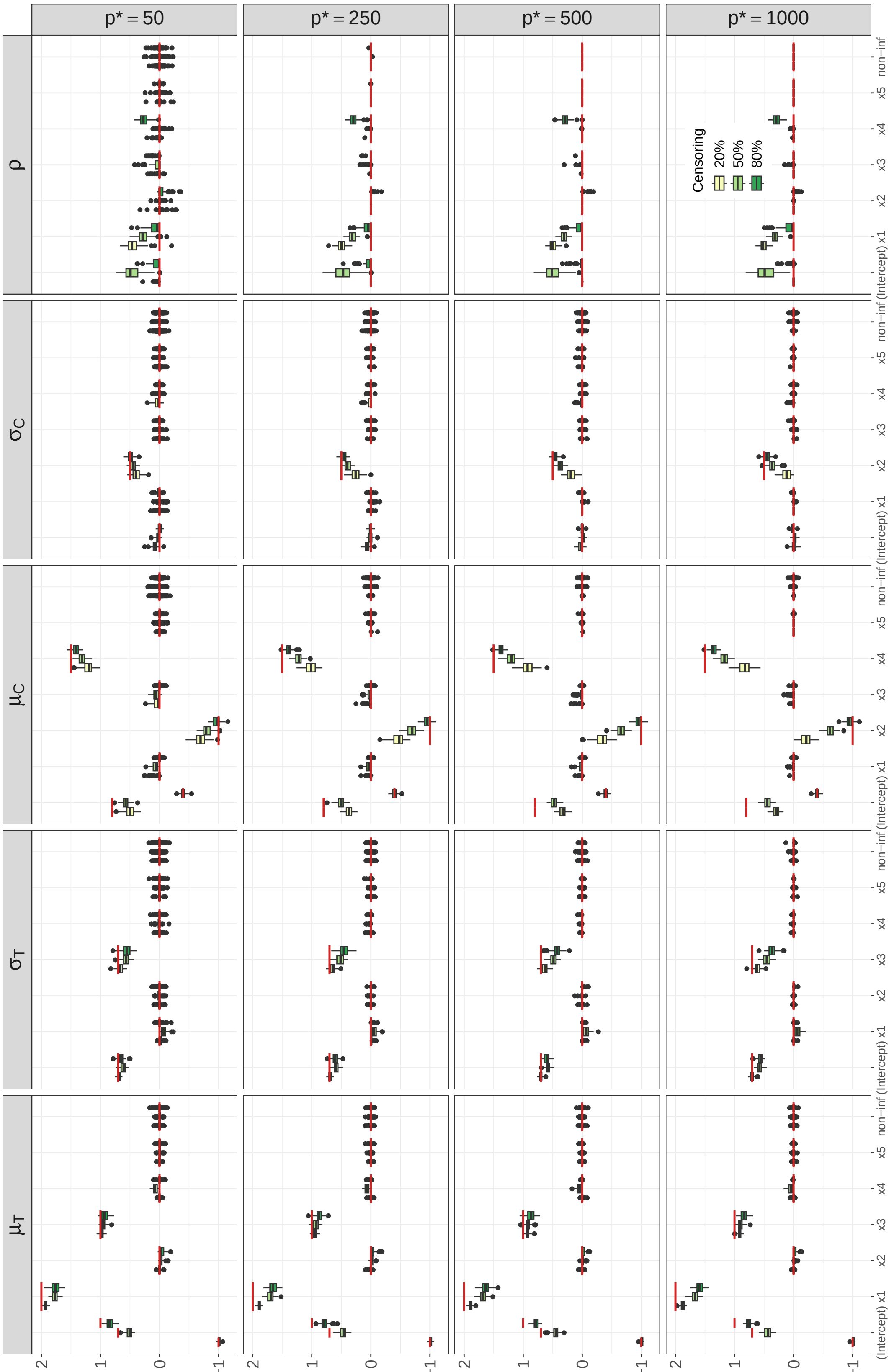

Fig. A5 Setting 3: Boxplot of estimated coefficients of CopBoostDepCens on the 100 replicates. Results are shown for each distribution parameter (columns) of the independent setting for the Gaussian copula with Weibull-distributed margins for different numbers of noise variables. The box colors represent the average proportions of censoring. The red horizontal lines show the true values for each.

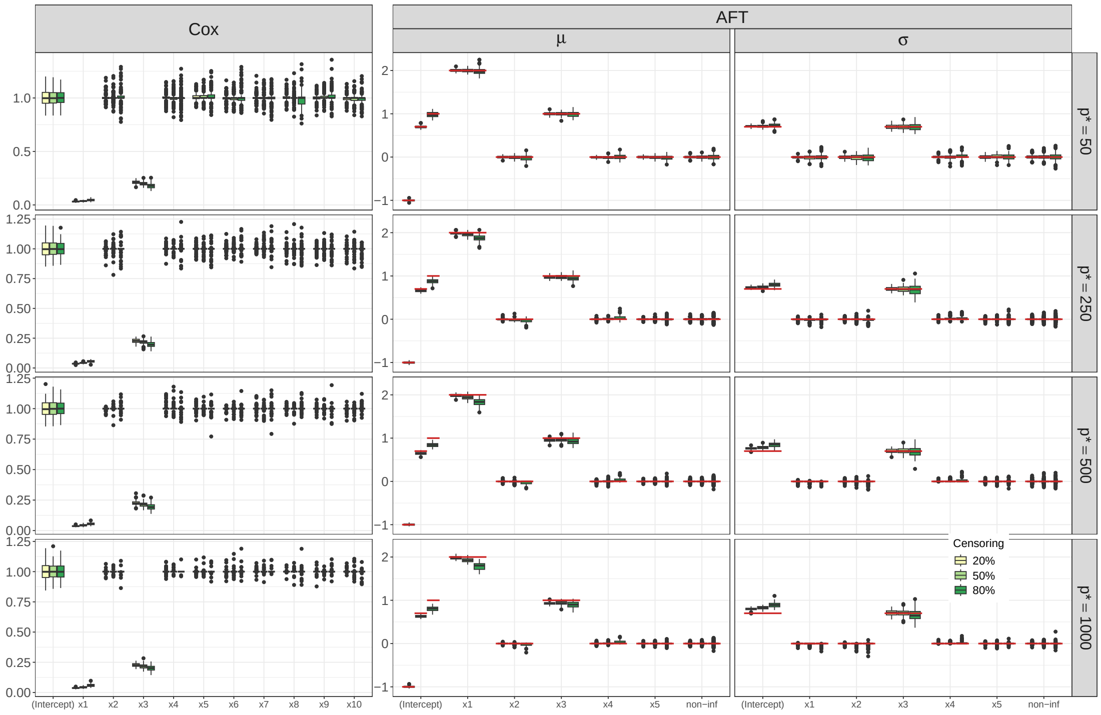

Fig. A6 Setting 3: Boxplot of estimated coefficients of the Cox and AFT models for different numbers of noise variables on the 100 replicates. The box colors represent the average proportions of censoring. The red horizontal lines show the true values for each.

	Parameter	Censoring 20% (Int)	x_1	x_2	x_3	x_4	x_5	non-inf.	Censoring 50% (Int)	x_1	x_2	x_3	x_4	x_5	non-inf.	Censoring 80% (Int)	x_1	x_2	x_3	x_4	x_5	non-inf.
p* = 50	μ_T	100	100	71	100	74	71	74.4	100	100	76	100	92	70	65.4	100	100	67	100	30	45	46.8
	σ_T	100	59	66	100	53	68	59.2	100	90	59	100	44	58	48.8	100	33	44	100	30	37	34.4
	μ_C	100	20	100	56	100	33	31.0	100	91	100	86	100	45	46.8	100	54	100	41	100	58	47.8
	σ_C	100	48	100	36	61	49	47.6	100	56	100	49	57	48	50.6	100	60	100	61	49	55	62.0
	ρ	100	100	16	14	15	14	10.4	100	100	16	44	24	17	17.4	100	60	38	16	100	15	11.0
p* = 250	μ_T	100	100	43	100	40	43	48.0	100	100	59	100	87	31	34.6	100	100	56	100	6	14	16.4
	σ_T	100	37	24	100	23	21	22.2	100	86	17	100	18	14	14.0	100	13	8	100	12	10	8.0
	μ_C	100	20	100	28	100	2	4.6	100	66	100	54	100	11	11.2	100	15	100	18	100	21	20.8
	σ_C	100	13	99	6	46	8	11.8	100	26	100	19	7	16	16.6	100	34	100	30	24	24	25.4
	ρ	100	100	0	1	1	0	0	100	100	0	10	6	0	0.2	100	57	8	3	100	1	0.2
p* = 500	μ_T	100	100	34	100	30	28	32.8	100	100	36	100	89	25	21.0	100	100	45	100	6	8	8.2
	σ_T	100	22	14	100	9	14	12.8	100	84	6	100	10	14	7.4	100	8	4	100	6	5	2.8
	μ_C	100	9	98	17	100	1	0.6	100	57	100	41	100	3	5.0	100	13	100	10	100	8	11.6
	σ_C	100	0	96	5	42	4	4.0	100	10	100	17	9	6	9.6	100	19	100	20	21	12	21.0
	ρ	100	100	0	1	0	0	0.0	100	100	0	5	4	0	0.0	100	57	12	1	99	0	0.0
p* = 1000	μ_T	100	100	20	100	31	21	20.8	100	100	26	100	86	15	15.8	100	100	51	100	2	5	4.2
	σ_T	100	17	5	100	7	6	4.6	100	82	5	100	4	7	3.2	100	6	2	100	4	2	1.0
	μ_C	100	1	93	4	100	0	0.2	100	33	100	29	100	0	2.6	100	5	100	7	100	3	7.2
	σ_C	100	0	88	2	27	1	0.8	100	10	100	14	5	6	7.2	100	11	100	21	12	11	15.0
	ρ	100	100	0	0	1	0	0.0	100	100	1	6	4	0	0.0	100	55	13	0	100	0	0.0

Table A15 Setting 3: Selection rates for CopBoostDepCens of the independent setting for the Gaussian copula with Weibull-distributed margins on the 100 replicates.

A.2 Clayton Copula

A.2.1 Setting 1

		Brier score			Integrated Brier score		
	Censoring	Copula	Cox	AFT	Copula	Cox	AFT
p* = 50	20%	0.07 (0.00)	0.44 (0.05)	0.08 (0.07)	0.07 (0.01)	0.13 (0.13)	0.07 (0.11)
	50%	0.07 (0.01)	0.40 (0.09)	0.11 (0.14)	0.06 (0.03)	0.15 (0.23)	0.11 (0.19)
	80%	0.04 (0.01)	0.25 (0.17)	0.12 (0.19)	0.05 (0.02)	0.18 (0.16)	0.15 (0.26)
p* = 250	20%	0.07 (0.00)	0.45 (0.08)	0.09 (0.11)	0.06 (0.00)	0.12 (0.11)	0.08 (0.12)
	50%	0.06 (0.01)	0.42 (0.12)	0.13 (0.18)	0.07 (0.03)	0.14 (0.21)	0.12 (0.18)
	80%	0.04 (0.01)	0.24 (0.16)	0.11 (0.17)	0.05 (0.01)	0.16 (0.14)	0.12 (0.20)
p* = 500	20%	0.07 (0.00)	0.44 (0.02)	0.08 (0.02)	0.06 (0.00)	0.11 (0.06)	0.06 (0.05)
	50%	0.06 (0.01)	0.43 (0.13)	0.14 (0.19)	0.06 (0.02)	0.17 (0.25)	0.13 (0.21)
	80%	0.04 (0.02)	0.24 (0.12)	0.10 (0.13)	0.05 (0.03)	0.18 (0.19)	0.12 (0.17)
p* = 1000	20%	0.07 (0.00)	0.45 (0.04)	0.09 (0.06)	0.06 (0.00)	0.13 (0.13)	0.07 (0.10)
	50%	0.06 (0.01)	0.43 (0.14)	0.13 (0.18)	0.07 (0.02)	0.16 (0.23)	0.12 (0.17)
	80%	0.04 (0.01)	0.22 (0.11)	0.08 (0.12)	0.05 (0.02)	0.15 (0.12)	0.10 (0.15)
p* = 2500	20%	0.07 (0.00)	0.45 (0.03)	0.08 (0.03)	0.07 (0.00)	0.12 (0.12)	0.06 (0.02)
	50%	0.07 (0)	0.45 (0.14)	0.15 (0.18)	0.07 (0.02)	0.16 (0.23)	0.12 (0.17)
	80%	0.04 (0.01)	0.21 (0.07)	0.07 (0.07)	0.06 (0.03)	0.14 (0.13)	0.10 (0.15)

Table A16 Setting 1: Mean (SD) of the Brier score and integrated Brier score for CopBoostDepCens, the Cox and the AFT models on the 100 replicates of the Clayton copula with Weibull-distributed margins for different numbers of noise variables.

		Integrated absolute error				Integrated squared error			
		Survival time			Censoring time	Survival time			Censoring time
	Censoring	Copula	Cox	AFT	Copula	Copula	Cox	AFT	Copula
p* = 50	20%	0.92 (0.09)	1.47 (0.21)	0.13 (0.02)	0.88 (0.11)	0.20 (0.02)	0.88 (0.14)	0.03 (0.01)	0.20 (0.03)
	50%	1.22 (0.08)	6.93 (1.64)	1.68 (0.21)	2.10 (0.13)	0.25 (0.04)	4.40 (1.23)	0.70 (0.14)	0.52 (0.04)
	80%	1.67 (0.30)	5.58 (2.34)	3.08 (0.80)	1.36 (0.17)	0.58 (0.18)	3.49 (1.81)	1.67 (0.59)	0.22 (0.02)
p* = 250	20%	0.94 (0.08)	1.46 (0.20)	0.13 (0.02)	0.84 (0.10)	0.21 (0.02)	0.87 (0.14)	0.03 (0.01)	0.18 (0.03)
	50%	1.32 (0.10)	6.87 (1.65)	1.48 (0.23)	2.03 (0.14)	0.32 (0.05)	4.26 (1.21)	0.54 (0.14)	0.47 (0.04)
	80%	1.49 (0.25)	5.42 (2.47)	2.28 (0.54)	1.34 (0.17)	0.45 (0.14)	3.30 (1.88)	0.99 (0.34)	0.21 (0.02)
p* = 500	20%	0.91 (0.08)	1.38 (0.19)	0.13 (0.02)	0.79 (0.11)	0.20 (0.02)	0.81 (0.13)	0.02 (0.01)	0.17 (0.03)
	50%	1.32 (0.09)	6.67 (1.31)	1.32 (0.20)	2.01 (0.12)	0.32 (0.05)	4.10 (0.96)	0.44 (0.12)	0.46 (0.03)
	80%	1.39 (0.21)	5.37 (1.89)	1.89 (0.43)	1.34 (0.15)	0.38 (0.12)	3.21 (1.40)	0.75 (0.27)	0.21 (0.02)
p* = 1000	20%	0.92 (0.07)	1.41 (0.18)	0.13 (0.02)	0.85 (0.11)	0.20 (0.01)	0.82 (0.12)	0.02 (0.01)	0.21 (0.04)
	50%	1.28 (0.09)	6.80 (1.48)	1.19 (0.19)	1.98 (0.13)	0.30 (0.05)	4.18 (1.08)	0.37 (0.10)	0.44 (0.03)
	80%	1.30 (0.17)	5.57 (2.37)	1.62 (0.36)	1.34 (0.18)	0.31 (0.08)	3.36 (1.77)	0.59 (0.22)	0.21 (0.02)
p* = 2500	20%	0.92 (0.08)	1.40 (0.20)	0.13 (0.01)	0.96 (0.13)	0.20 (0.02)	0.81 (0.14)	0.02 (0.01)	0.28 (0.05)
	50%	1.23 (0.08)	7.00 (1.38)	1.12 (0.15)	2.00 (0.12)	0.26 (0.04)	4.30 (1.01)	0.35 (0.09)	0.44 (0.02)
	80%	1.20 (0.16)	5.40 (2.13)	1.41 (0.25)	1.31 (0.16)	0.26 (0.05)	3.21 (1.55)	0.46 (0.14)	0.20 (0.02)

Table A17 Setting 1: Means (SDs) of the integrated absolute and integrated squared error for CopBoostDepCens, the Cox and the AFT models on the 100 replicates of the Clayton copula with Weibull-distributed margins for different numbers of noise variables.

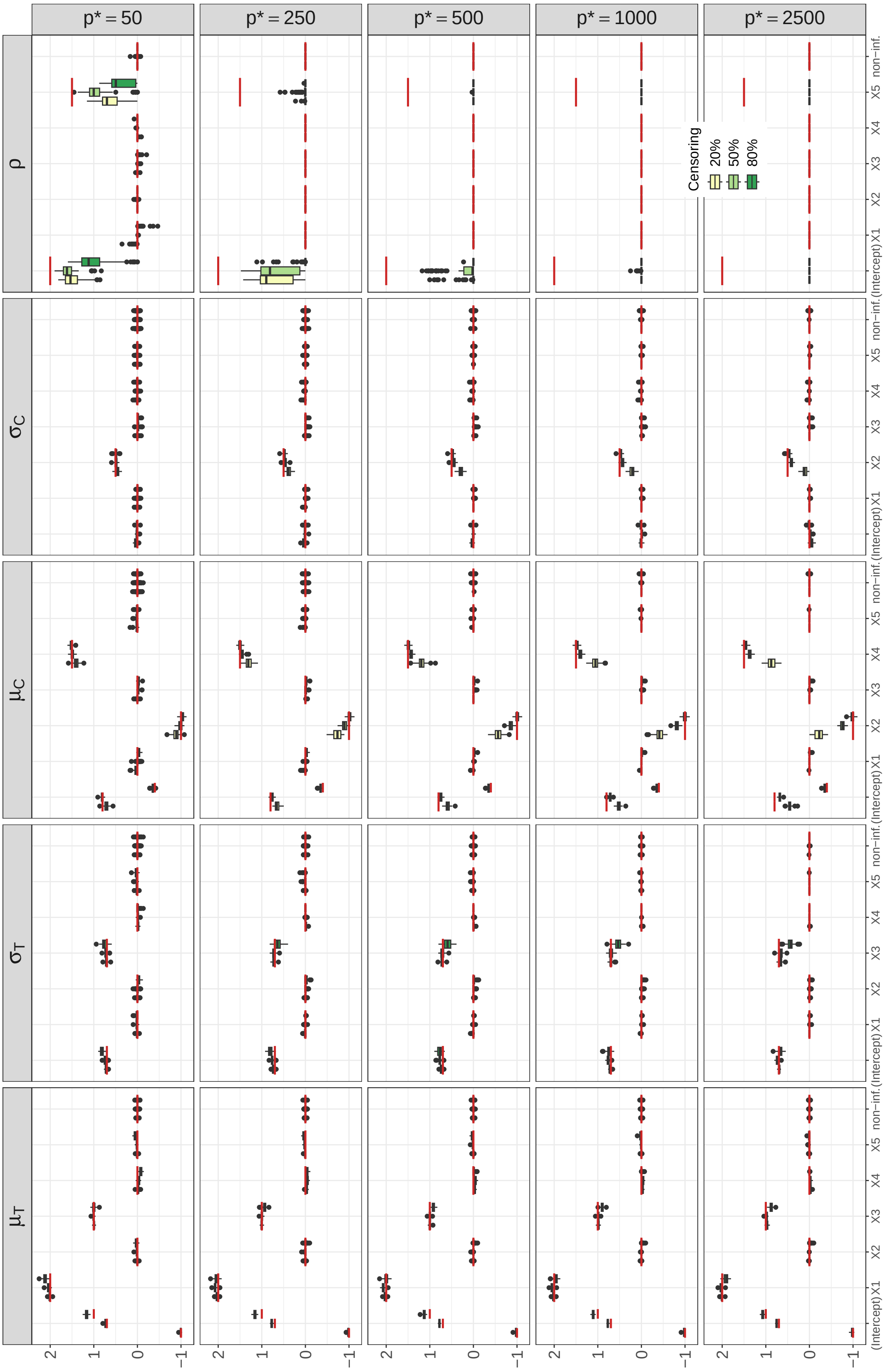

Fig. A7 Setting 1: Boxplot of estimated coefficients of CopBoostDepCens on the 100 replicates. Results are shown for each distribution parameter (columns) of the Clayton copula with Weibull-distributed margins for different numbers of noise variables. The box colors represent the average proportions of censoring. The red horizontal lines show the true values for each.

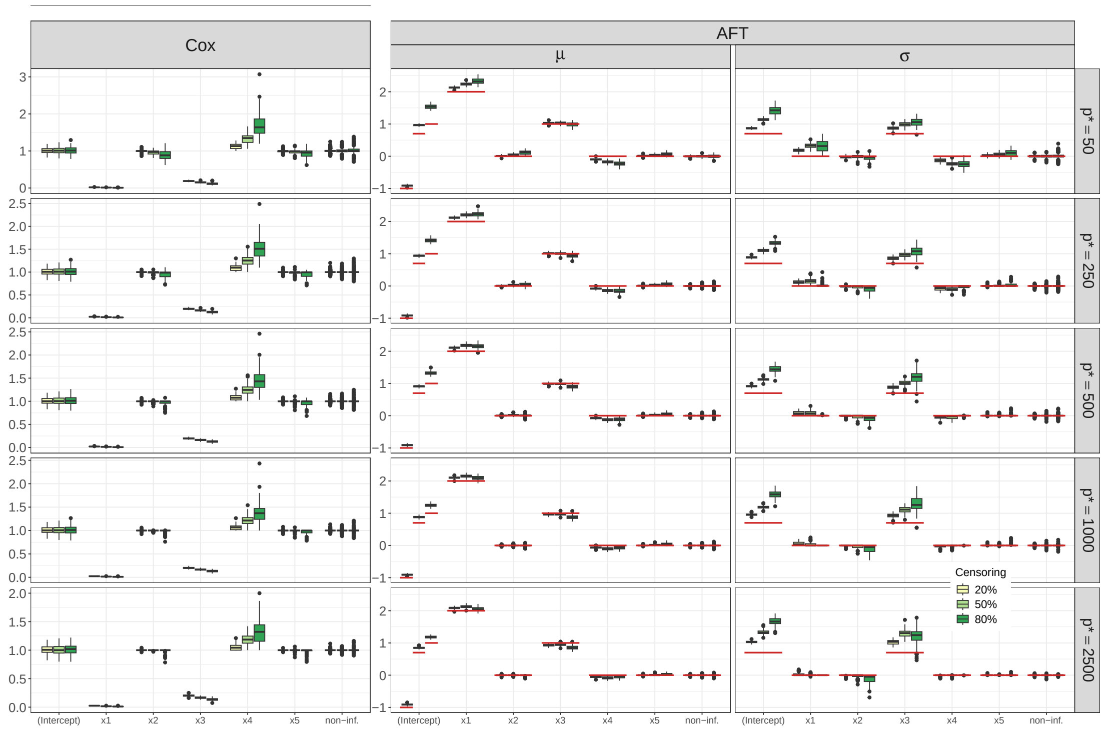

Fig. A8 Setting 1: Boxplot of estimated coefficients of the Cox and AFT models for different numbers of noise variables on the 100 replicates. The box colors represent the average proportions of censoring. The red horizontal lines show the true values for each.

	Censoring	Copula	Cox	AFT
p* = 50	20%	154.53 (37.90)	1.05 (0.63)	1.69 (1.01)
	50%	118.19 (24.44)	1.15 (0.78)	2.54 (1.01)
	80%	125.91 (35.85)	2.31 (0.77)	3.50 (1.18)
p* = 250	20%	55.91 (15.36)	0.8 (0.17)	0.81 (0.27)
	50%	46.73 (16.62)	0.77 (0.19)	1.25 (0.45)
	80%	45.04 (9.75)	1.60 (0.47)	1.81 (0.29)
p* = 500	20%	37.20 (8.89)	0.85 (0.26)	0.89 (0.34)
	50%	60.17 (18.60)	1.03 (0.26)	2.32 (0.37)
	80%	51.60 (7.17)	1.87 (0.61)	2.46 (0.48)
p* = 1000	20%	34.77 (5.03)	0.87 (0.14)	1.18 (0.20)
	50%	38.23 (6.00)	0.84 (0.23)	1.75 (0.37)
	80%	33.33 (5.04)	1.25 (0.92)	1.14 (0.63)
p* = 2500	20%	20.87 (2.67)	0.69 (0.03)	0.92 (0.09)
	50%	24.52 (3.29)	0.64 (0.04)	1.28 (0.24)
	80%	36.56 (4.57)	1.31 (0.48)	2.28 (0.24)

Table A18 Setting 1: Means (SDs) of the runtime for CopBoostDepCens, the Cox and AFT models of the Clayton copula with Weibull distributed margins on the 100 replicates.

	Parameter	Censoring 20% (Int)	x_1	x_2	x_3	x_4	x_5	non-inf.	Censoring 50% (Int)	x_1	x_2	x_3	x_4	x_5	non-inf.	Censoring 80% (Int)	x_1	x_2	x_3	x_4	x_5	non-inf.
p* = 50	μ_T	100	100	86	100	100	92	81.6	100	100	89	100	100	98	82.0	100	100	87	100	98	98	62.2
	σ_T	100	72	68	100	86	83	66.0	100	66	69	100	68	75	60.8	100	51	66	100	45	71	37.8
	μ_C	100	66	100	41	100	57	42.6	100	50	100	69	100	66	58.8	100	75	100	72	100	58	54.0
	σ_C	100	48	100	51	55	58	57.0	100	66	100	74	61	67	68.2	100	63	100	64	63	68	60.8
	ρ	100	15	0	3	3	96	0.0	100	2	6	4	2	98	1.8	98	14	0	8	1	80	0.0
p* = 250	μ_T	100	100	57	100	100	63	54.2	100	100	49	100	97	81	41.0	100	100	20	100	70	91	22.8
	σ_T	100	52	37	100	63	43	27.4	100	18	36	100	21	35	13.6	100	4	49	100	0	15	4.2
	μ_C	100	20	100	4	100	14	6.6	100	9	100	46	100	14	11.6	100	65	100	61	100	20	18.2
	σ_C	100	8	100	23	30	13	14.0	100	26	100	44	21	20	17.4	100	28	100	40	32	30	27.4
	ρ	85	0	0	0	0	4	0.0	97	0	0	0	0	29	0.0	19	0	0	0	0	1	0.0
p* = 500	μ_T	100	100	34	100	98	44	34.0	100	100	28	100	94	70	26.2	100	100	17	100	53	81	14.0
	σ_T	100	36	27	100	45	14	7.6	100	6	33	100	9	21	5.6	100	5	38	100	0	7	1.8
	μ_C	100	3	100	0	100	1	0.2	100	2	100	36	100	4	4.0	100	56	100	49	100	11	11.8
	σ_C	100	0	100	9	27	2	1.4	100	19	100	31	16	10	8.4	100	19	100	35	26	21	20.4
	ρ	17	0	0	0	0	0	0.0	55	0	0	0	0	1	0.0	1	0	0	0	0	0	0.0
p* = 1000	μ_T	100	100	18	100	95	29	20.4	100	100	5	100	88	60	15.0	100	100	18	100	22	70	7.2
	σ_T	100	6	20	100	24	4	1.8	100	3	24	100	1	6	1.8	100	4	28	100	0	2	0.6
	μ_C	100	1	100	0	100	0	0.0	100	0	100	17	100	1	0.6	100	45	100	43	100	6	7.0
	σ_C	100	0	100	4	19	0	0.0	100	14	100	21	10	3	1.6	100	11	100	25	17	14	13.0
	ρ	0	0	0	0	0	0	0.0	7	0	0	0	0	0	0.0	0	0	0	0	0	0	0.0
p* = 2500	μ_T	100	100	8	100	89	18	7.6	100	100	2	100	75	38	9.6	100	100	14	100	2	48	2.6
	σ_T	100	0	10	100	7	0	0.2	100	3	13	100	0	0	0.8	100	3	24	100	0	1	0.0
	μ_C	100	1	99	0	100	0	0.0	100	0	100	6	100	0	0.0	100	30	100	33	100	1	4.2
	σ_C	100	0	98	0	12	0	0.0	100	4	100	11	2	1	0.2	100	4	100	13	9	4	7.0
	ρ	0	0	0	0	0	0	0.0	0	0	0	0	0	0	0.0	0	0	0	0	0	0	0.0

Table A19 Setting 1: Selection rates for CopBoostDepCens of the Clayton copula with Weibull distributed margins on the 100 replicates.

A.2.2 Setting 3

	Censoring	Brier score Copula	Brier score Cox	Brier score AFT	Integrated Brier score Copula	Integrated Brier score Cox	Integrated Brier score AFT
p* = 50	20%	0.08 (0.00)	0.47 (0.11)	0.12 (0.15)	0.07 (0.01)	0.14 (0.17)	0.10 (0.16)
	50%	0.08 (0.01)	0.47 (0.14)	0.17 (0.21)	0.09 (0.02)	0.18 (0.24)	0.16 (0.23)
	80%	0.06 (0.02)	0.30 (0.09)	0.11 (0.10)	0.08 (0.04)	0.15 (0.17)	0.14 (0.17)
p* = 250	20%	0.08 (0.00)	0.47 (0.08)	0.12 (0.14)	0.07 (0.01)	0.16 (0.20)	0.11 (0.18)
	50%	0.08 (0.01)	0.46 (0.12)	0.15 (0.17)	0.10 (0.02)	0.17 (0.23)	0.14 (0.20)
	80%	0.06 (0.01)	0.31 (0.14)	0.15 (0.17)	0.09 (0.05)	0.20 (0.25)	0.19 (0.23)
p* = 500	20%	0.08 (0.00)	0.47 (0.08)	0.11 (0.13)	0.08 (0.01)	0.15 (0.18)	0.09 (0.13)
	50%	0.08 (0.01)	0.46 (0.11)	0.15 (0.16)	0.10 (0.03)	0.18 (0.24)	0.15 (0.19)
	80%	0.06 (0.01)	0.30 (0.10)	0.12 (0.12)	0.09 (0.04)	0.16 (0.21)	0.15 (0.18)
p* = 1000	20%	0.08 (0.00)	0.48 (0.09)	0.13 (0.16)	0.08 (0.01)	0.17 (0.21)	0.13 (0.22)
	50%	0.09 (0.01)	0.45 (0.10)	0.14 (0.15)	0.10 (0.03)	0.16 (0.20)	0.14 (0.19)
	80%	0.07 (0.01)	0.31 (0.12)	0.13 (0.14)	0.10 (0.06)	0.15 (0.16)	0.15 (0.18)

Table A20 Setting 3: Mean (SD) of the Brier score and integrated Brier score for CopBoostDepCens, the Cox and the AFT models on the 100 replicates of the independent setting for the Clayton copula with Weibull-distributed margins for different numbers of noise variables.

	Censoring	Integrated absolute error: Survival time Copula	Survival time Cox	Survival time AFT	Censoring time Copula	Integrated squared error: Survival time Copula	Survival time Cox	Survival time AFT	Censoring time Copula
p* =50	20%	0.92 (0.09)	1.20 (0.22)	0.04 (0.01)	1.01 (0.17)	0.21 (0.02)	0.66 (0.15)	0.00 (0.00)	0.29 (0.07)
	50%	1.36 (0.15)	4.48 (1.30)	0.25 (0.06)	2.00 (0.20)	0.32 (0.06)	2.27 (0.82)	0.02 (0.01)	0.46 (0.04)
	80%	1.60 (0.41)	2.70 (1.72)	0.39 (0.16)	1.28 (0.23)	0.56 (0.23)	1.14 (1.00)	0.04 (0.03)	0.21 (0.03)
p* = 250	20%	0.93 (0.12)	1.13 (0.21)	0.06 (0.01)	1.10 (0.19)	0.21 (0.02)	0.60 (0.13)	0.01 (0.00)	0.35 (0.08)
	50%	1.44 (0.17)	4.18 (1.6)	0.40 (0.08)	1.97 (0.24)	0.39 (0.07)	2.03 (0.99)	0.05 (0.02)	0.47 (0.05)
	80%	1.92 (0.42)	2.74 (1.63)	0.69 (0.24)	1.27 (0.22)	0.81 (0.24)	1.13 (0.95)	0.14 (0.09)	0.20 (0.03)
p* = 500	20%	0.94 (0.11)	1.14 (0.23)	0.07 (0.01)	1.12 (0.18)	0.21 (0.02)	0.60 (0.14)	0.01 (0.00)	0.37 (0.07)
	50%	1.48 (0.16)	4.12 (1.13)	0.48 (0.09)	1.99 (0.19)	0.41 (0.08)	1.98 (0.70)	0.07 (0.03)	0.48 (0.05)
	80%	1.98 (0.40)	2.6 (1.22)	0.82 (0.24)	1.26 (0.19)	0.87 (0.24)	1.03 (0.66)	0.20 (0.09)	0.20 (0.02)
p* = 1000	20%	0.95 (0.13)	1.15 (0.24)	0.08 (0.01)	1.17 (0.21)	0.22 (0.03)	0.61 (0.16)	0.01 (0.00)	0.4 (0.09)
	50%	1.54 (0.16)	4.30 (1.36)	0.54 (0.10)	2.02 (0.20)	0.46 (0.08)	2.09 (0.83)	0.10 (0.03)	0.50 (0.05)
	80%	2.13 (0.44)	2.72 (1.61)	0.98 (0.28)	1.26 (0.20)	0.99 (0.26)	1.10 (0.90)	0.28 (0.11)	0.20 (0.02)

Table A21 Setting 3: Means (SDs) of the integrated absolute and integrated squared error for CopBoostDepCens, the Cox and the AFT models on the 100 replicates of the independent setting for the Clayton copula with Weibull-distributed margins for different numbers of noise variables.

	Censoring	Copula	Cox	AFT
p* = 50	20%	30.23 (8.72)	0.59 (0.32)	0.58 (0.12)
	50%	27.47 (7.05)	0.59 (0.31)	0.98 (0.19)
	80%	29.78 (8.55)	0.7 (0.4)	1.68 (0.31)
p* = 250	20%	24.93 (3.91)	0.67 (0.02)	0.61 (0.04)
	50%	23.86 (3.66)	0.59 (0.02)	0.93 (0.12)
	80%	27.58 (3.11)	0.66 (0.28)	1.58 (0.17)
p* = 500	20%	20.21 (3.57)	0.67 (0.03)	0.60 (0.04)
	50%	21.53 (3.69)	0.61 (0.04)	0.89 (0.17)
	80%	27.05 (2.94)	0.68 (0.25)	1.56 (0.25)
p* = 1000	20%	17.41 (2.42)	0.65 (0.01)	0.61 (0.02)
	50%	18.99 (2.93)	0.58 (0.03)	0.86 (0.16)
	80%	23.46 (3.21)	0.62 (0.25)	1.41 (0.26)

Table A22 Setting 3: Means (SDs) of the runtime for CopBoostDepCens, the Cox and AFT models of the Clayton copula with Weibull distributed margins on the 100 replicates.

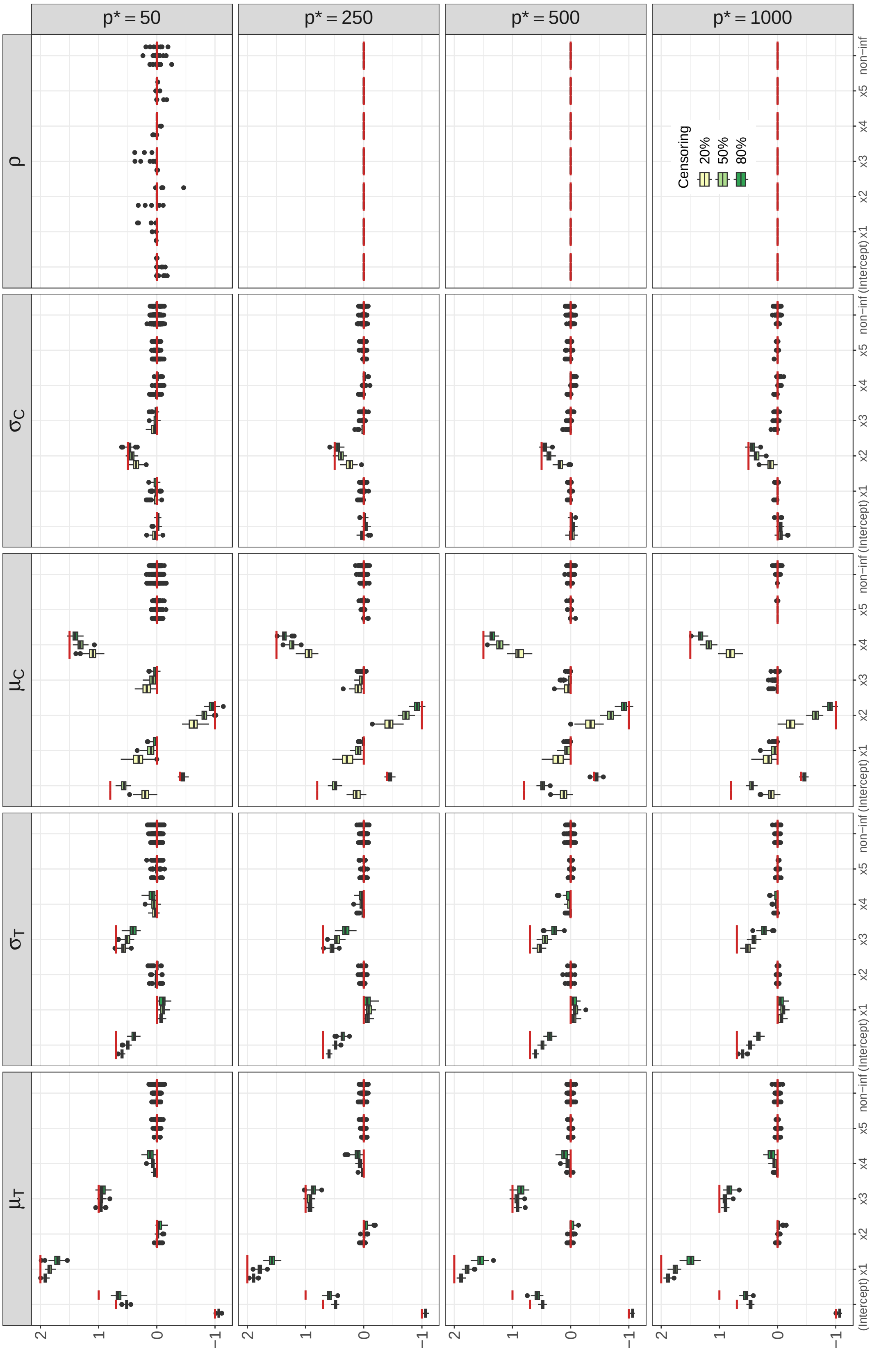

Fig. A9 Setting 3: Boxplot of estimated coefficients of CopBoostDepCens on the 100 replicates. Results are shown for each distribution parameter (columns) of the independent setting for the Clayton copula with Weibull-distributed margins for different numbers of noise variables. The box colors represent the average proportions of censoring. The red horizontal lines show the true values for each.

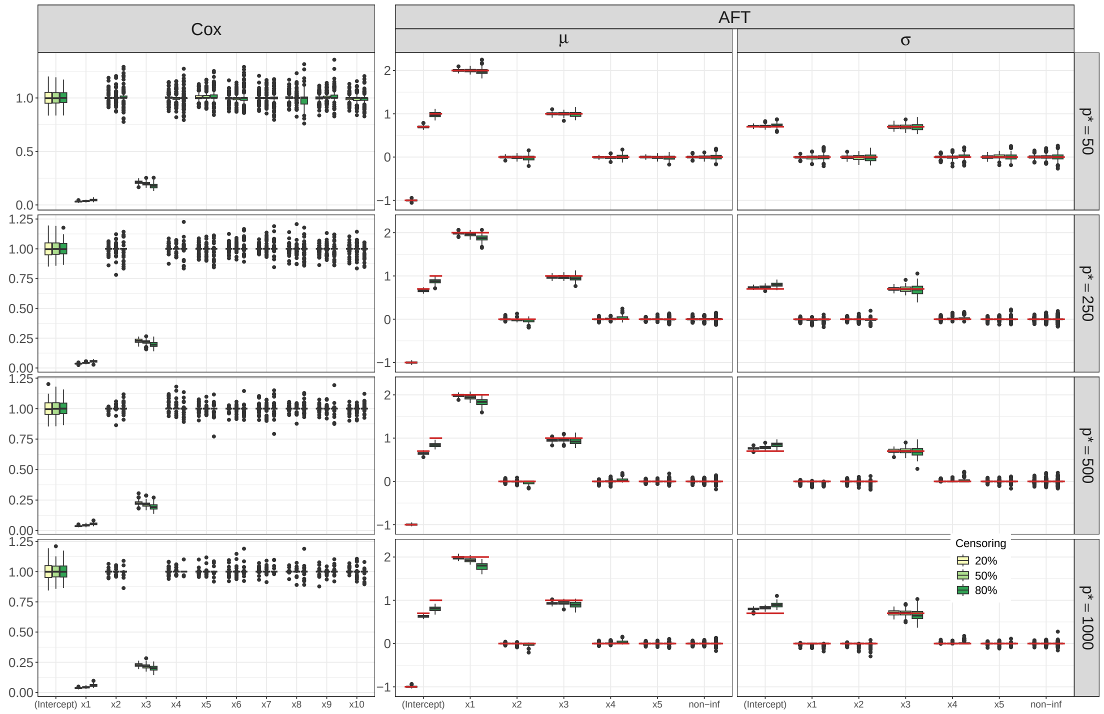

Fig. A10 Setting 3: Boxplot of estimated coefficients of the Cox and AFT models for different numbers of noise variables on the 100 replicates. The box colors represent the average proportions of censoring. The red horizontal lines show the true values for each.

		Censoring 20%							Censoring 50%							Censoring 80%						
	Parameter	(Int)	x_1	x_2	x_3	x_4	x_5	non-inf.	(Int)	x_1	x_2	x_3	x_4	x_5	non-inf.	(Int)	x_1	x_2	x_3	x_4	x_5	non-inf.
p* = 50	μ_T	100	100	67	100	90	75	74.0	100	100	71	100	98	63	63.0	100	100	76	100	95	46	48.0
	σ_T	100	97	62	100	88	72	64.0	100	97	61	100	81	57	51.6	100	88	58	100	87	50	42.4
	μ_C	100	99	100	100	100	37	37.0	100	91	100	87	100	41	45.6	100	66	100	71	100	56	51.6
	σ_C	100	55	100	82	52	54	54.4	100	57	100	63	47	49	51.4	100	66	100	62	58	58	64.8
	ρ	19	1	5	2	4	4	2.4	16	2	0	7	2	2	2.4	10	4	4	3	0	1	1.6
p* = 250	μ_T	100	100	35	100	69	40	50.0	100	100	48	100	94	30	32.2	100	100	59	100	96	9	12.8
	σ_T	100	91	25	100	48	27	26.2	100	95	17	100	68	15	17.0	100	75	9	100	66	11	11.2
	μ_C	100	99	100	82	100	3	5.0	100	90	100	57	100	13	11.2	100	33	100	29	100	22	21.8
	σ_C	100	21	100	44	21	10	15.2	100	22	100	25	18	16	18.2	100	35	100	33	48	29	28.4
	ρ	0	0	0	0	0	0	0.0	0	0	0	0	0	0	0.0	0	0	0	0	0	0	0.0
p* = 500	μ_T	100	100	30	100	55	27	34.2	100	100	28	100	88	25	17.4	100	100	45	100	92	10	7.2
	σ_T	100	89	17	100	37	18	16.6	100	89	9	100	58	14	10.6	100	67	7	100	55	10	5.0
	μ_C	100	93	98	63	100	2	1.0	100	79	100	43	100	2	4.6	100	24	100	17	100	12	11.2
	σ_C	100	14	99	21	11	6	6.2	100	8	100	14	19	7	11.0	100	24	100	24	31	13	21.4
	ρ	0	0	0	0	0	0	0.0	0	0	0	0	0	0	0.0	0	0	0	0	0	0	0.0
p* = 1000	μ_T	100	100	14	100	41	20	20.0	100	100	20	100	85	11	13.6	100	100	47	100	94	3	4.0
	σ_T	100	81	7	100	15	10	7.8	100	92	7	100	40	8	4.2	100	79	6	100	44	3	3.0
	μ_C	100	96	97	32	100	0	0.2	100	74	100	29	100	0	2.6	100	16	100	10	100	3	7.4
	σ_C	100	6	95	11	9	1	2.2	100	0	100	10	8	5	9.4	100	13	100	22	31	10	16.4
	ρ	0	0	0	0	0	0	0.0	0	0	0	0	0	0	0.0	0	0	0	0	0	0	0.0

Table A23 Setting 3: Selection rates for CopBoostDepCens of the independent setting for the Clayton copula with Weibull-distributed margins on the 100 replicates.

A.3 Gumbel Copula

A.3.1 Setting 1

	Censoring	Brier score Copula	Brier score Cox	Brier score AFT	Integrated Brier score Copula	Integrated Brier score Cox	Integrated Brier score AFT
p* = 50	20%	0.08 (0.01)	0.45 (0.06)	0.09 (0.07)	0.07 (0.02)	0.12 (0.11)	0.07 (0.10)
	50%	0.09 (0.01)	0.40 (0.09)	0.10 (0.12)	0.07 (0.07)	0.11 (0.15)	0.09 (0.13)
	80%	0.07 (0.02)	0.21 (0.08)	0.08 (0.09)	0.09 (0.07)	0.17 (0.12)	0.11 (0.17)
p* = 250	20%	0.08 (0.00)	0.45 (0.02)	0.08 (0.03)	0.08 (0.01)	0.11 (0.07)	0.06 (0.06)
	50%	0.09 (0.02)	0.42 (0.11)	0.12 (0.16)	0.09 (0.06)	0.13 (0.20)	0.12 (0.20)
	80%	0.07 (0.02)	0.24 (0.13)	0.11 (0.15)	0.09 (0.07)	0.18 (0.15)	0.15 (0.23)
p* = 500	20%	0.08 (0.00)	0.45 (0.02)	0.08 (0.01)	0.08 (0.01)	0.11 (0.07)	0.06 (0.01)
	50%	0.09 (0.01)	0.41 (0.08)	0.11 (0.12)	0.08 (0.06)	0.13 (0.19)	0.10 (0.16)
	80%	0.07 (0.02)	0.22 (0.11)	0.09 (0.13)	0.07 (0.06)	0.19 (0.18)	0.13 (0.20)
p* = 1000	20%	0.08 (0.00)	0.45 (0.06)	0.08 (0.09)	0.09 (0.01)	0.11 (0.09)	0.06 (0.08)
	50%	0.09 (0.01)	0.42 (0.10)	0.11 (0.13)	0.09 (0.07)	0.13 (0.19)	0.09 (0.12)
	80%	0.07 (0.02)	0.22 (0.09)	0.08 (0.09)	0.09 (0.07)	0.17 (0.16)	0.10 (0.15)
p* = 2500	20%	0.09 (0.00)	0.45 (0.05)	0.08 (0.05)	0.10 (0.01)	0.10 (0.02)	0.06 (0.01)
	50%	0.10 (0.01)	0.43 (0.12)	0.13 (0.17)	0.10 (0.06)	0.15 (0.23)	0.11 (0.17)
	80%	0.07 (0.02)	0.24 (0.12)	0.10 (0.13)	0.08 (0.06)	0.18 (0.19)	0.13 (0.17)

Table A24 Setting 1: Mean (SD) of the Brier score and integrated Brier score for CopBoostDepCens, the Cox and the AFT models on the 100 replicates of the Gumbel copula with Weibull-distributed margins for different numbers of noise variables.

		Integrated absolute error				Integrated squared error			
		Survival time			Censoring time	Survival time			Censoring time
	Censoring	Copula	Cox	AFT	Copula	Copula	Cox	AFT	Copula
p* =50	20%	0.94 (0.09)	1.49 (0.21)	0.13 (0.02)	0.88 (0.11)	0.21 (0.02)	0.89 (0.15)	0.03 (0.01)	0.19 (0.03)
	50%	1.14 (0.05)	7.60 (1.83)	1.98 (0.28)	2.07 (0.14)	0.19 (0.01)	4.83 (1.33)	0.86 (0.18)	0.47 (0.03)
	80%	1.26 (0.16)	6.75 (3.16)	4.11 (1.11)	1.46 (0.20)	0.26 (0.06)	4.34 (2.45)	2.42 (0.86)	0.22 (0.02)
p* = 250	20%	0.96 (0.07)	1.47 (0.19)	0.13 (0.02)	0.87 (0.09)	0.21 (0.01)	0.86 (0.13)	0.02 (0.01)	0.19 (0.03)
	50%	1.15 (0.05)	7.57 (1.60)	1.74 (0.25)	2.04 (0.11)	0.19 (0.01)	4.72 (1.17)	0.66 (0.16)	0.44 (0.02)
	80%	1.31 (0.17)	6.54 (2.67)	3.20 (0.79)	1.47 (0.19)	0.29 (0.08)	4.11 (2.06)	1.60 (0.56)	0.21 (0.02)
p* = 500	20%	0.96 (0.09)	1.46 (0.20)	0.13 (0.02)	0.92 (0.13)	0.21 (0.02)	0.85 (0.14)	0.02 (0.01)	0.22 (0.05)
	50%	1.14 (0.06)	7.41 (1.80)	1.54 (0.24)	2.04 (0.14)	0.19 (0.01)	4.58 (1.30)	0.54 (0.14)	0.44 (0.02)
	80%	1.31 (0.18)	6.42 (3.04)	2.51 (0.58)	1.45 (0.21)	0.31 (0.08)	3.98 (2.30)	1.12 (0.39)	0.21 (0.02)
p* = 1000	20%	0.98 (0.07)	1.46 (0.17)	0.13 (0.01)	1.04 (0.13)	0.22 (0.01)	0.84 (0.11)	0.02 (0.01)	0.30 (0.06)
	50%	1.14 (0.05)	7.11 (1.61)	1.34 (0.22)	2.01 (0.13)	0.19 (0.01)	4.36 (1.16)	0.44 (0.12)	0.44 (0.02)
	80%	1.36 (0.21)	6.05 (2.35)	2.02 (0.52)	1.42 (0.17)	0.35 (0.11)	3.67 (1.76)	0.82 (0.34)	0.20 (0.02)
p* = 2500	20%	1.01 (0.09)	1.45 (0.19)	0.13 (0.01)	1.21 (0.14)	0.22 (0.02)	0.84 (0.13)	0.02 (0.00)	0.41 (0.06)
	50%	1.17 (0.06)	7.49 (1.74)	1.22 (0.17)	2.06 (0.13)	0.21 (0.02)	4.59 (1.25)	0.39 (0.10)	0.46 (0.03)
	80%	1.46 (0.21)	6.31 (2.92)	1.66 (0.37)	1.42 (0.18)	0.43 (0.12)	3.86 (2.25)	0.60 (0.22)	0.20 (0.02)

Table A25 Setting 1: Means (SDs) of the integrated absolute and integrated squared error for CopBoostDepCens, the Cox and the AFT models on the 100 replicates of the Gumbel copula with Weibull-distributed margins for different numbers of noise variables.

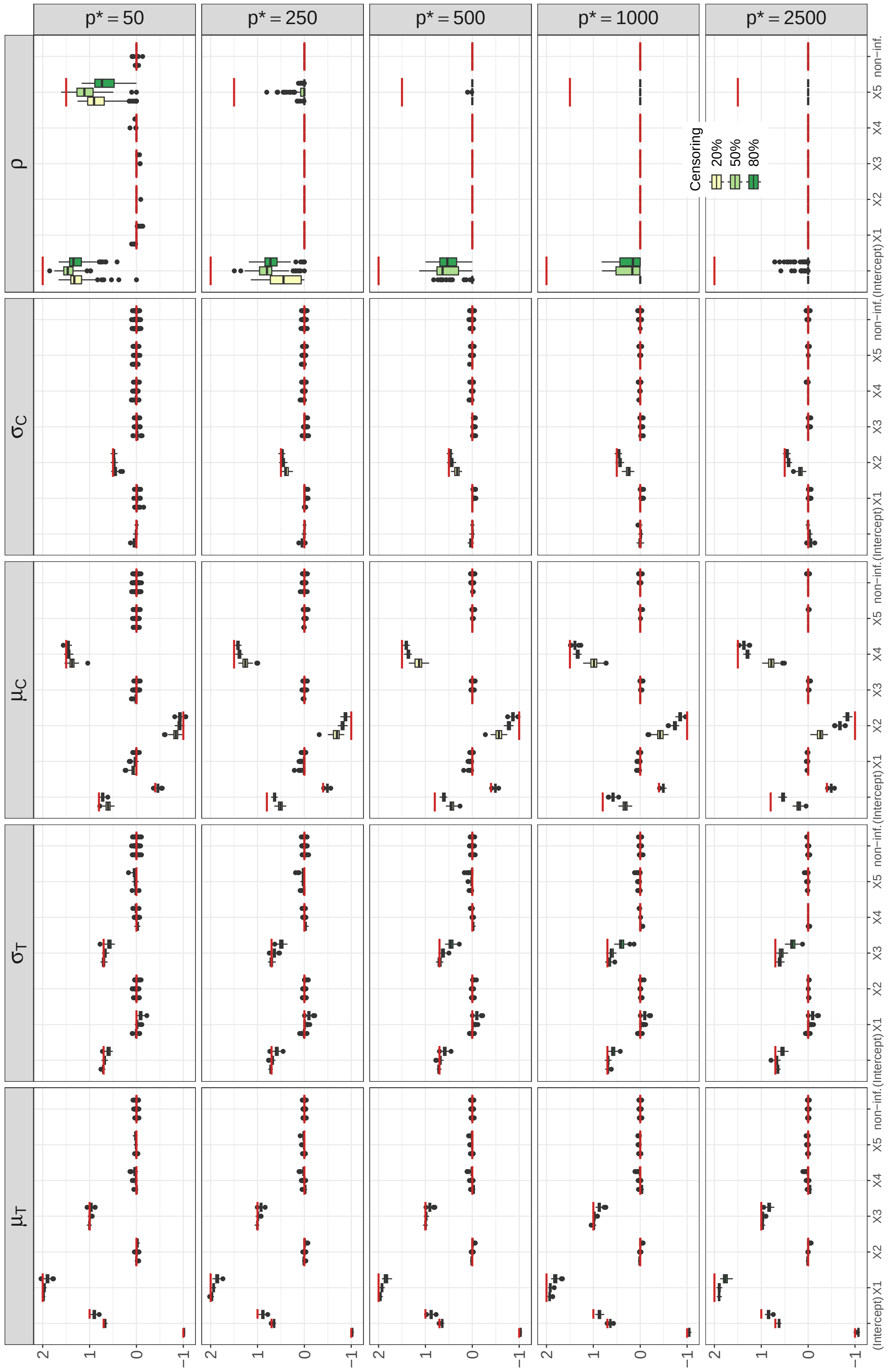

Fig. A11 Setting 1: Boxplot of estimated coefficients of CopBoostDepCens on the 100 replicates. Results are shown for each distribution parameter (columns) of the Gumbel copula with Weibull-distributed margins for different numbers of noise variables. The box colors represent the average proportions of censoring. The red horizontal lines show the true values for each.

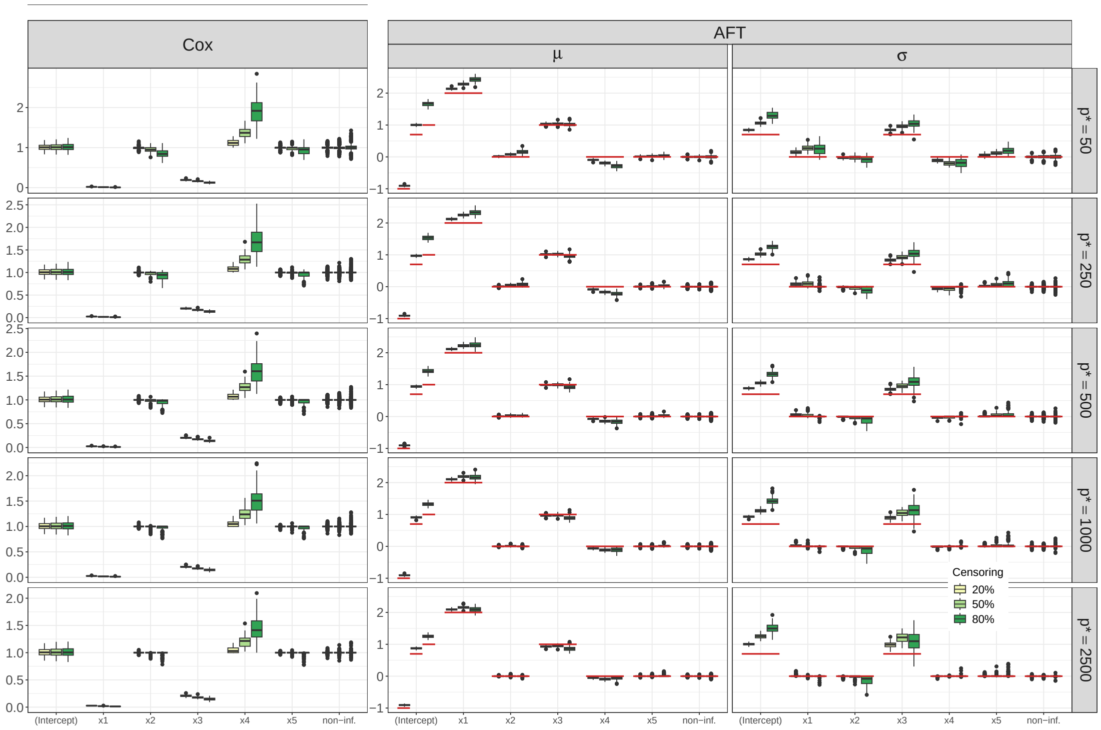

Fig. A12 Setting 1: Boxplot of estimated coefficients of the Cox and AFT models for different numbers of noise variables on the 100 replicates. The box colors represent the average proportions of censoring. The red horizontal lines show the true values for each.

	Censoring	Copula	Cox	AFT
p* = 50	20%	144.22 (36.08)	1.28 (0.96)	1.62 (1.18)
	50%	88.39 (23.09)	1.02 (0.65)	2.13 (0.97)
	80%	125.03 (32.51)	1.92 (0.45)	3.62 (1.01)
p* = 250	20%	58.31 (15.37)	0.77 (0.13)	0.87 (0.19)
	50%	53.27 (13.22)	0.8 (0.19)	1.49 (0.34)
	80%	67.14 (12.56)	1.80 (0.58)	2.83 (0.67)
p* = 500	20%	47.68 (12.62)	1.01 (0.41)	1.03 (0.47)
	50%	45.16 (10.96)	0.89 (0.30)	1.49 (0.51)
	80%	64.00 (14.36)	2.25 (0.89)	2.9 (0.72)
p* = 1000	20%	35.92 (6.83)	1.09 (0.42)	1.23 (0.51)
	50%	42.45 (12.01)	1.04 (0.50)	1.69 (0.57)
	80%	44.91 (11.97)	1.75 (0.81)	2.50 (0.70)
p* = 2500	20%	24.82 (3.91)	0.69 (0.06)	0.90 (0.19)
	50%	29.07 (5.23)	0.68 (0.09)	1.65 (0.42)
	80%	43.40 (11.42)	1.34 (0.54)	2.60 (0.59)

Table A26 Setting 1: Means (SDs) of the runtime for CopBoostDepCens, the Cox and AFT models of the Gumbel copula with Weibull distributed margins on the 100 replicates.

	Parameter	Censoring 20% (Int)	x_1	x_2	x_3	x_4	x_5	non-inf.	Censoring 50% (Int)	x_1	x_2	x_3	x_4	x_5	non-inf.	Censoring 80% (Int)	x_1	x_2	x_3	x_4	x_5	non-inf.
p* = 50	μ_T	100	100	97	100	100	85	86.0	100	100	76	100	84	85	79.8	100	100	78	100	93	89	68.0
	σ_T	100	76	68	100	89	87	64.8	100	75	51	100	63	94	64.0	100	100	48	100	58	83	49.0
	μ_C	100	78	100	47	100	50	46.0	100	67	100	58	100	60	54.4	100	60	100	56	100	65	55.6
	σ_C	100	48	100	67	63	62	59.8	100	63	100	69	57	67	62.4	100	67	100	71	67	65	67.6
	ρ	99	5	0	0	0	97	0.4	100	0	1	2	3	99	1.2	100	9	0	2	3	95	0.0
p* = 250	μ_T	100	100	92	100	100	58	58.8	100	100	41	100	43	58	43.8	100	100	48	100	68	65	29.0
	σ_T	100	59	24	100	68	53	28.0	100	45	18	100	20	71	18.8	100	100	16	100	16	65	9.6
	μ_C	100	34	100	4	100	4	4.6	100	43	100	14	100	18	11.6	100	25	100	22	100	29	21.0
	σ_C	100	5	100	37	22	16	11.4	100	38	100	36	22	25	19.8	100	42	100	40	38	25	30.6
	ρ	84	0	0	0	0	8	0.0	99	0	0	0	0	45	0.0	98	0	0	0	0	9	0.0
p* = 500	μ_T	100	100	84	100	100	45	42.2	100	100	24	100	30	52	32.6	100	100	40	100	57	45	17.6
	σ_T	100	49	13	100	59	32	13.0	100	42	10	100	11	53	8.6	100	100	10	100	10	47	4.2
	μ_C	100	8	100	0	100	0	0.6	100	32	100	7	100	6	4.0	100	13	100	16	100	19	13.0
	σ_C	100	0	100	21	8	1	2.8	100	33	100	29	9	13	10.2	100	33	100	37	27	17	22.4
	ρ	18	0	0	0	0	0	0.0	92	0	0	0	0	2	0.0	93	0	0	0	0	0	0.0
p* = 1000	μ_T	100	100	79	100	100	28	23.8	100	100	12	100	19	37	19.6	100	100	36	100	43	34	7.6
	σ_T	100	44	4	100	43	8	3.6	100	42	7	100	1	32	3.4	100	100	8	100	2	20	1.4
	μ_C	100	5	100	0	100	0	0.0	100	17	100	3	100	1	1.2	100	7	100	9	100	9	6.8
	σ_C	100	0	100	4	1	0	0.2	100	29	100	22	3	3	5.0	100	22	100	29	19	11	12.6
	ρ	0	0	0	0	0	0	0.0	75	0	0	0	0	0	0.0	69	0	0	0	0	0	0.0
p* = 2500	μ_T	100	100	79	100	99	10	9.8	100	100	5	100	12	22	8.2	100	100	32	100	43	18	2.2
	σ_T	100	42	3	100	19	2	0.6	100	42	2	100	0	8	1.0	100	100	2	100	0	10	0.2
	μ_C	100	1	100	0	100	0	0.0	100	4	100	2	100	0	0.0	100	3	100	7	100	5	2.2
	σ_C	100	0	100	0	0	0	0.0	100	25	100	15	0	1	1.8	100	12	100	24	11	9	5.6
	ρ	0	0	0	0	0	0	0.0	19	0	0	0	0	0	0.0	25	0	0	0	0	0	0.0

Table A27 Setting 1: Selection rates for CopBoostDepCens of the Gumbel copula with Weibull distributed margins on the 100 replicates.

A.3.2 Setting 3

	Censoring	Brier score Copula	Brier score Cox	Brier score AFT	Integrated Brier score Copula	Integrated Brier score Cox	Integrated Brier score AFT
p* = 50	20%	0.09 (0.01)	0.47 (0.11)	0.12 (0.15)	0.09 (0.02)	0.14 (0.17)	0.10 (0.16)
	50%	0.09 (0.02)	0.47 (0.14)	0.17 (0.21)	0.09 (0.05)	0.18 (0.24)	0.16 (0.23)
	80%	0.09 (0.03)	0.30 (0.09)	0.11 (0.10)	0.11 (0.07)	0.15 (0.17)	0.14 (0.17)
p* = 250	20%	0.09 (0.00)	0.47 (0.08)	0.12 (0.14)	0.09 (0.01)	0.16 (0.20)	0.11 (0.18)
	50%	0.11 (0.01)	0.46 (0.12)	0.15 (0.17)	0.14 (0.05)	0.17 (0.23)	0.14 (0.20)
	80%	0.09 (0.02)	0.31 (0.14)	0.15 (0.17)	0.14 (0.09)	0.20 (0.25)	0.19 (0.23)"
p* = 500	20%	0.09 (0.00)	0.47 (0.08)	0.11 (0.13)	0.10 (0.01)	0.15 (0.18)	0.09 (0.13)
	50%	0.11 (0.02)	0.46 (0.11)	0.15 (0.16)	0.15 (0.07)	0.18 (0.24)	0.15 (0.19)
	80%	0.09 (0.02)	0.30 (0.10)	0.12 (0.12)	0.14 (0.09)	0.16 (0.21)	0.15 (0.18)
p* = 1000	20%	0.09 (0.00)	0.48 (0.09)	0.13 (0.16)	0.11 (0.01)	0.17 (0.21)	0.13 (0.22)
	50%	0.12 (0.02)	0.45 (0.10)	0.14 (0.15)	0.17 (0.06)	0.16 (0.20)	0.14 (0.19)
	80%	0.10 (0.02)	0.31 (0.12)	0.13 (0.14)	0.18 (0.09)	0.15 (0.16)	0.15 (0.18)

Table A28 Setting 3: Mean (SD) of the Brier score and integrated Brier score for CopBoostDepCens, the Cox and the AFT models on the 100 replicates of the independent setting for the Gumbel copula with Weibull-distributed margins for different numbers of noise variables.

		Integrated absolute error				Integrated squared error			
		Survival time			Censoring time	Survival time			Censoring time
	Censoring	Copula	Cox	AFT	Copula	Copula	Cox	AFT	Copula
p* =50	20%	0.96 (0.10)	1.20 (0.22)	0.04 (0.01)	1.28 (0.18)	0.22 (0.02)	0.66 (0.15)	0.00 (0.00)	0.45 (0.08)
	50%	1.54 (0.25)	4.48 (1.30)	0.25 (0.06)	2.07 (0.21)	0.45 (0.16)	2.27 (0.82)	0.02 (0.01)	0.50 (0.05)
	80%	2.58 (0.50)	2.70 (1.72)	0.39 (0.16)	1.33 (0.24)	1.34 (0.32)	1.14 (1.00)	0.04 (0.03)	0.21 (0.03)
p* = 250	20%	0.99 (0.12)	1.13 (0.21)	0.06 (0.01)	1.36 (0.19)	0.23 (0.03)	0.60 (0.13)	0.01 (0.00)	0.51 (0.07)
	50%	1.96 (0.21)	4.18 (1.60)	0.40 (0.08)	2.14 (0.25)	0.77 (0.12)	2.03 (0.99)	0.05 (0.02)	0.58 (0.06)
	80%	2.89 (0.47)	2.74 (1.63)	0.69 (0.24)	1.32 (0.23)	1.61 (0.29)	1.13 (0.95)	0.14 (0.09)	0.21 (0.03)
p* = 500	20%	1.01 (0.11)	1.14 (0.23)	0.07 (0.01)	1.38 (0.18)	0.24 (0.02)	0.60 (0.14)	0.01 (0.00)	0.52 (0.07)
	50%	2.04 (0.18)	4.12 (1.13)	0.48 (0.09)	2.18 (0.20)	0.84 (0.10)	1.98 (0.70)	0.07 (0.03)	0.60 (0.06)
	80%	2.94 (0.42)	2.60 (1.22)	0.82 (0.24)	1.30 (0.19)	1.66 (0.27)	1.03 (0.66)	0.20 (0.09)	0.20 (0.02)
p* = 1000	20%	1.03 (0.13)	1.15 (0.24)	0.08 (0.01)	1.41 (0.20)	0.25 (0.03)	0.61 (0.16)	0.01 (0.00)	0.55 (0.08)
	50%	2.13 (0.19)	4.30 (1.36)	0.54 (0.10)	2.22 (0.21)	0.91 (0.11)	2.09 (0.83)	0.10 (0.03)	0.63 (0.06)
	80%	3.04 (0.45)	2.72 (1.61)	0.98 (0.28)	1.30 (0.20)	1.75 (0.28)	1.10 (0.90)	0.28 (0.11)	0.20 (0.02)

Table A29 Setting 3: Means (SDs) of the integrated absolute and integrated squared error for CopBoostDepCens, the Cox and the AFT models on the 100 replicates of the independent setting for the Gumbel copula with Weibull-distributed margins for different numbers of noise variables.

	Censoring	Copula	Cox	AFT
p* = 50	20%	44.60 (31.10)	0.59 (0.32)	0.56 (0.12)
	50%	43.41 (31.23)	0.57 (0.30)	0.92 (0.16)
	80%	43.57 (33.71)	0.69 (0.39)	1.66 (0.32)
p* = 250	20%	26.65 (4.18)	0.65 (0.04)	0.57 (0.04)
	50%	23.85 (4.06)	0.57 (0.02)	0.87 (0.11)
	80%	26.93 (3.82)	0.63 (0.27)	1.46 (0.16)
p* = 500	20%	21.10 (3.70)	0.50 (0.03)	0.56 (0.05)
	50%	19.45 (3.38)	0.49 (0.03)	0.86 (0.18)
	80%	21.95 (3.77)	0.52 (0.18)	1.37 (0.24)
p* = 1000	20%	20.08 (2.89)	0.66 (0.02)	0.60 (0.02)
	50%	19.04 (2.75)	0.58 (0.03)	0.85 (0.15)
	80%	23.60 (3.96)	0.62 (0.25)	1.37 (0.25)

Table A30 Setting 3: Means (SDs) of the runtime for CopBoostDepCens, the Cox and AFT models of the Gumbel copula with Weibull distributed margins on the 100 replicates.

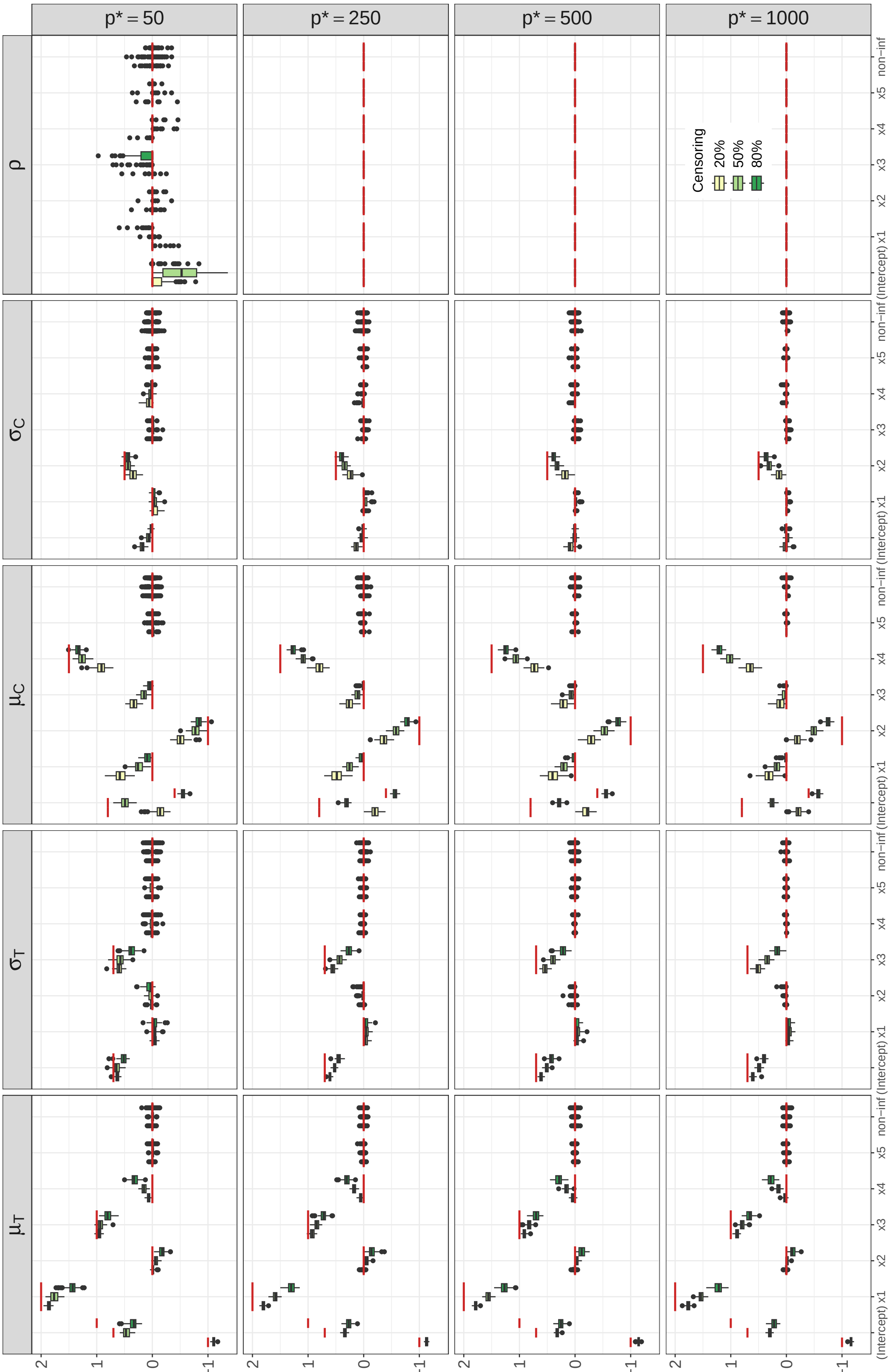

Fig. A13 Setting 3: Results of the estimated linear effects for the independent setting for the Gumbel copula with Weibull margins for different numbers of covariates p from 100 replicates. The horizontal red lines correspond to the true values.

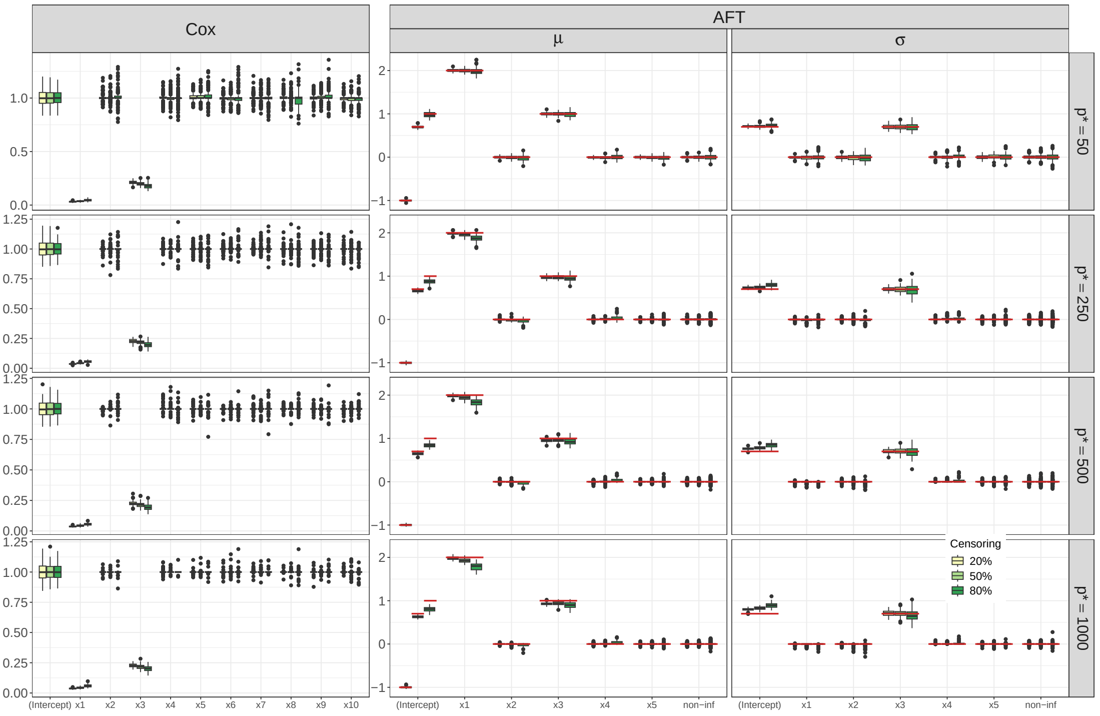

Fig. A14 Setting 3: Boxplot of estimated coefficients of the Cox and AFT models for different numbers of noise variables on the 100 replicates. The box colors represent the average proportions of censoring. The red horizontal lines show the true values for each.

	Parameter	Censoring 20% (Int)	x_1	x_2	x_3	x_4	x_5	non-inf.	Censoring 50% (Int)	x_1	x_2	x_3	x_4	x_5	non-inf.	Censoring 80% (Int)	x_1	x_2	x_3	x_4	x_5	non-inf.
p* = 50	μ_T	100	100	72	100	72	57	52.2	100	100	96	100	100	58	50.6	100	100	100	93	100	43	40.8
	σ_T	100	96	86	100	80	86	80.6	100	98	89	100	92	75	73.8	100	98	76	100	81	66	65.0
	μ_C	100	100	100	95	100	53	61.2	100	100	100	80	100	71	69.2	100	100	100	90	100	69	65.4
	σ_C	100	70	100	98	97	75	69.4	100	78	100	97	89	77	75.4	100	71	100	90	75	76	70.6
	ρ	88	20	59	20	31	8	10.2	84	12	23	10	16	9	10.2	52	17	5	6	10	7	6.0
p* = 250	μ_T	100	100	20	99	17	18	13.8	100	100	79	98	92	14	11.2	100	100	99	63	99	9	4.8
	σ_T	100	91	55	100	65	37	44.0	100	96	61	100	74	36	32.0	100	96	23	100	51	21	22.0
	μ_C	100	100	100	65	100	19	20.6	100	100	100	20	100	31	28.2	100	96	100	66	100	34	30.2
	σ_C	100	31	100	93	79	28	31.4	100	37	100	86	53	35	34.8	100	39	100	53	40	35	39.2
	ρ	0	0	0	0	0	0	0.0	0	0	0	0	0	0	0.0	0	0	0	0	0	0	0.0
p* = 500	μ_T	100	100	6	100	7	9	3.2	100	100	60	86	58	5	3.6	100	100	86	25	93	1	1.8
	σ_T	100	85	51	100	43	31	33.2	100	95	53	100	68	17	21.8	100	95	16	100	41	14	16.2
	μ_C	100	100	100	29	100	8	7.6	100	100	100	12	100	15	17.4	100	85	100	59	100	26	22.2
	σ_C	100	10	99	87	89	23	20.6	100	30	100	76	50	31	25.2	100	27	100	44	30	31	28.6
	ρ	0	0	0	0	0	0	0.0	0	0	0	0	0	0	0.0	0	0	0	0	0	0	0.0
p* = 1000	μ_T	100	100	1	100	1	0	0.8	100	100	37	55	31	0	1.8	100	100	65	5	79	0	0.2
	σ_T	100	85	51	100	27	17	20.8	100	89	55	100	49	15	14.4	100	80	21	100	29	9	6.4
	μ_C	100	100	100	9	100	0	3.8	100	100	100	11	100	6	9.6	100	70	100	45	100	9	11.2
	σ_C	100	10	95	78	85	14	8.8	100	23	100	61	54	24	17.8	100	25	100	28	21	22	21.6
	ρ	0	0	0	0	0	0	0.0	0	0	0	0	0	0	0.0	0	0	0	0	0	0	0.0

Table A31 Setting 3: Selection rates for CopBoostDepCens of the independent setting with Weibull distributed margins on the 100 replicates.

B Simulation results with log-normal marginal distributions

B.1 Gaussian copula

B.1.1 Setting 1

	Censoring	Brier score Copula	Brier score Cox	Brier score AFT	Integrated Brier score Copula	Integrated Brier score Cox	Integrated Brier score AFT
p* = 50	20%	0.22 (0.01)	0.76 (0.13)	0.53 (0.34)	0.36 (0.04)	0.61 (0.41)	0.61 (0.41)
	50%	0.24 (0.02)	0.81 (0.11)	0.50 (0.28)	0.40 (0.07)	0.55 (0.34)	0.55 (0.34)
	80%	0.16 (0.02)	0.58 (0.19)	0.39 (0.23)	0.27 (0.10)	0.45 (0.27)	0.45 (0.27)
p* = 250	20%	0.24 (0.01)	0.73 (0.12)	0.46 (0.31)	0.41 (0.05)	0.56 (0.41)	0.56 (0.41)
	50%	0.27 (0.02)	0.80 (0.11)	0.47 (0.27)	0.48 (0.06)	0.52 (0.33)	0.52 (0.34)
	80%	0.18 (0.02)	0.59 (0.21)	0.40 (0.23)	0.35 (0.08)	0.43 (0.27)	0.43 (0.27)
p* = 500	20%	0.26 (0.01)	0.75 (0.12)	0.53 (0.31)	0.43 (0.04)	0.65 (0.40)	0.64 (0.41)
	50%	0.29 (0.02)	0.79 (0.11)	0.49 (0.28)	0.53 (0.07)	0.57 (0.35)	0.56 (0.35)
	80%	0.20 (0.02)	0.57 (0.18)	0.39 (0.23)	0.39 (0.08)	0.45 (0.28)	0.46 (0.28)
p* = 1000	20%	0.27 (0.01)	0.77 (0.12)	0.55 (0.32)	0.44 (0.04)	0.65 (0.41)	0.64 (0.41)
	50%	0.33 (0.03)	0.80 (0.10)	0.50 (0.27)	0.58 (0.07)	0.57 (0.35)	0.56 (0.36)
	80%	0.22 (0.02)	0.58 (0.18)	0.42 (0.22)	0.43 (0.04)	0.49 (0.30)	0.49 (0.30)
p* = 2500	20%	0.28 (0.01)	0.76 (0.13)	0.53 (0.32)	0.45 (0.04)	0.64 (0.40)	0.63 (0.40)
	50%	0.36 (0.02)	0.79 (0.11)	0.46 (0.28)	0.61 (0.05)	0.50 (0.33)	0.50 (0.34)
	80%	0.23 (0.02)	0.55 (0.17)	0.37 (0.20)	0.44 (0.04)	0.41 (0.25)	0.41 (0.25)

Table A32 Setting 1: Mean (SD) of the Brier score and integrated Brier score for CopBoostDepCens, the Cox and the AFT models on the 100 replicates of the Gaussian copula with log-normal distributed margins for different numbers of noise variables.

	Censoring	Integrated absolute error: Survival time, Copula	Survival time, Cox	Survival time, AFT	Censoring time, Copula	Integrated squared error: Survival time, Copula	Survival time, Cox	Survival time, AFT	Censoring time, Copula
p* = 50	20%	1.72 (0.88)	17.98 (22.64)	2.69 (2.84)	1.26 (0.25)	0.06 (0.04)	3.88 (5.61)	0.14 (0.16)	0.16 (0.06)
	50%	4.67 (2.72)	40.28 (62.49)	15.29 (13.17)	0.60 (0.13)	0.25 (0.16)	13.89 (27.20)	2.30 (1.95)	0.03 (0.01)
	80%	5.11 (3.57)	20.57 (30.21)	8.88 (6.13)	0.16 (0.04)	0.66 (0.48)	9.47 (17.68)	1.98 (1.22)	0.01 (0.00)
p* = 250	20%	2.92 (1.49)	18.78 (33.24)	1.97 (1.20)	1.91 (0.29)	0.17 (0.07)	3.86 (8.28)	0.07 (0.05)	0.36 (0.11)
	50%	8.17 (4.67)	38.40 (49.91)	8.74 (6.29)	1.05 (0.14)	0.75 (0.36)	12.25 (17.43)	0.91 (0.62)	0.10 (0.03)
	80%	7.40 (4.59)	20.52 (24.19)	5.78 (3.66)	0.26 (0.05)	1.35 (0.78)	8.99 (11.69)	1.02 (0.59)	0.02 (0.01)
p* = 500	20%	3.24 (1.16)	14.94 (14.85)	1.84 (0.85)	2.42 (0.32)	0.24 (0.08)	2.94 (4.11)	0.07 (0.04)	0.58 (0.14)
	50%	9.43 (4.13)	33.48 (47.78)	6.38 (4.8)	1.38 (0.27)	1.12 (0.51)	10.7 (21.27)	0.55 (0.45)	0.18 (0.09)
	80%	8.16 (4.22)	18.68 (25.79)	4.34 (2.43)	0.34 (0.05)	1.80 (0.86)	8.33 (15.80)	0.65 (0.39)	0.03 (0.01)
p* = 1000	20%	3.71 (1.33)	15.56 (14.94)	1.99 (0.81)	2.77 (0.22)	0.31 (0.09)	3.05 (3.94)	0.09 (0.03)	0.73 (0.10)
	50%	12.68 (4.93)	33.88 (26.65)	5.73 (2.82)	1.98 (0.31)	2.02 (0.73)	10.49 (10.02)	0.43 (0.24)	0.37 (0.12)
	80%	9.29 (3.76)	18.12 (13.26)	3.97 (1.82)	0.41 (0.04)	2.33 (0.84)	7.79 (6.81)	0.53 (0.27)	0.05 (0.01)
p* = 2500	20%	3.91 (1.11)	13.69 (10.36)	2.12 (0.65)	2.99 (0.20)	0.36 (0.09)	2.47 (2.46)	0.10 (0.03)	0.81 (0.09)
	50%	13.12 (3.93)	29.72 (22.02)	5.10 (2.21)	2.29 (0.11)	2.35 (0.51)	8.86 (8.38)	0.37 (0.16)	0.49 (0.05)
	80%	9.47 (3.58)	16.99 (13.21)	3.86 (2.03)	0.45 (0.04)	2.56 (0.82)	7.19 (6.79)	0.50 (0.29)	0.06 (0.01)

Table A33 Setting 1: Means (SDs) of the integrated absolute and integrated squared error for CopBoostDepCens, the Cox and the AFT models on the 100 replicates of the Gaussian copula with log-normal distributed margins for different numbers of noise variables.

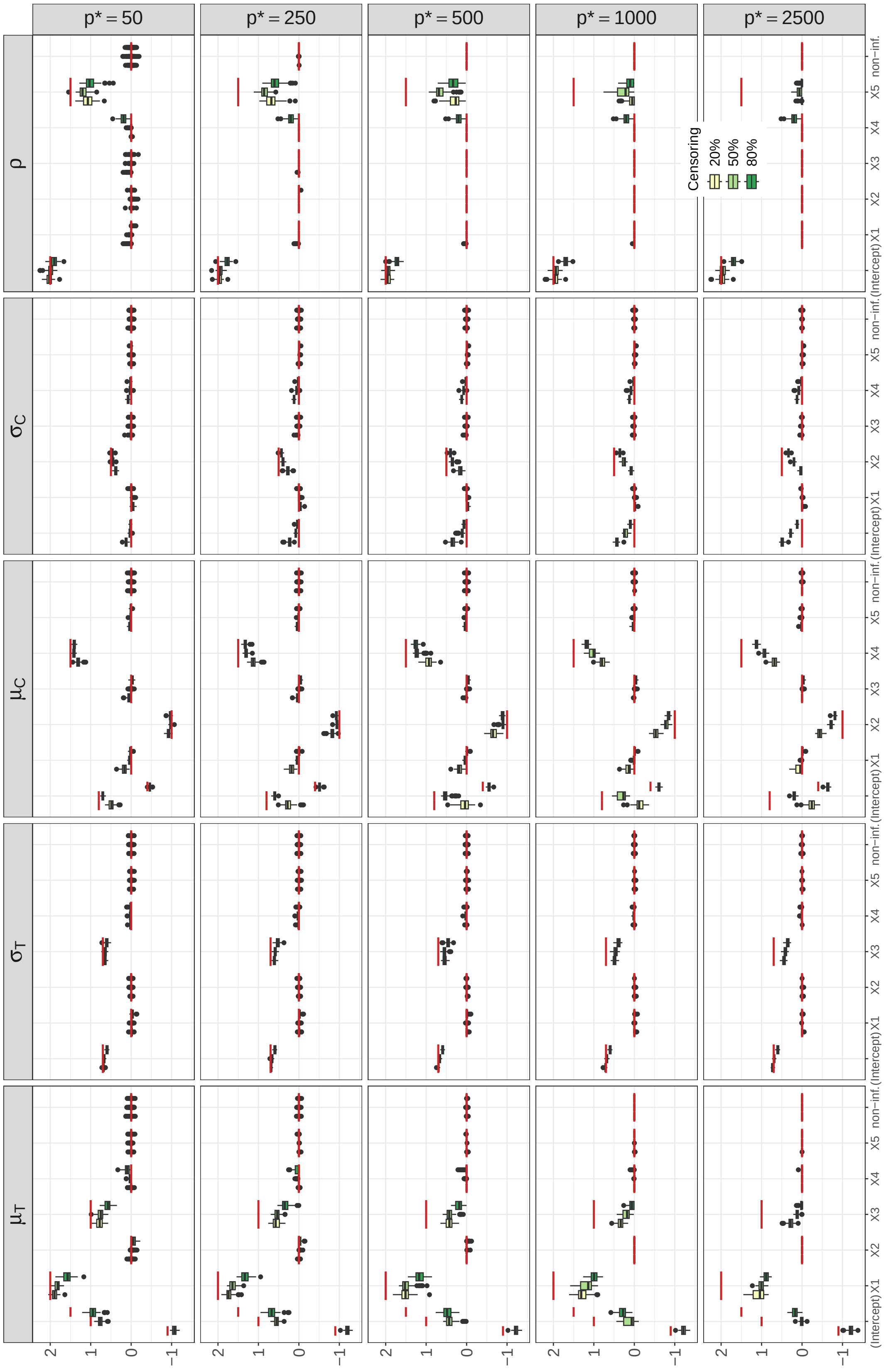

Fig. A15 Setting 1: Results of the estimated linear effects for the Gaussian copula with log-normal margins for different numbers of covariates p from 100 replicates. The horizontal red lines correspond to the true values.

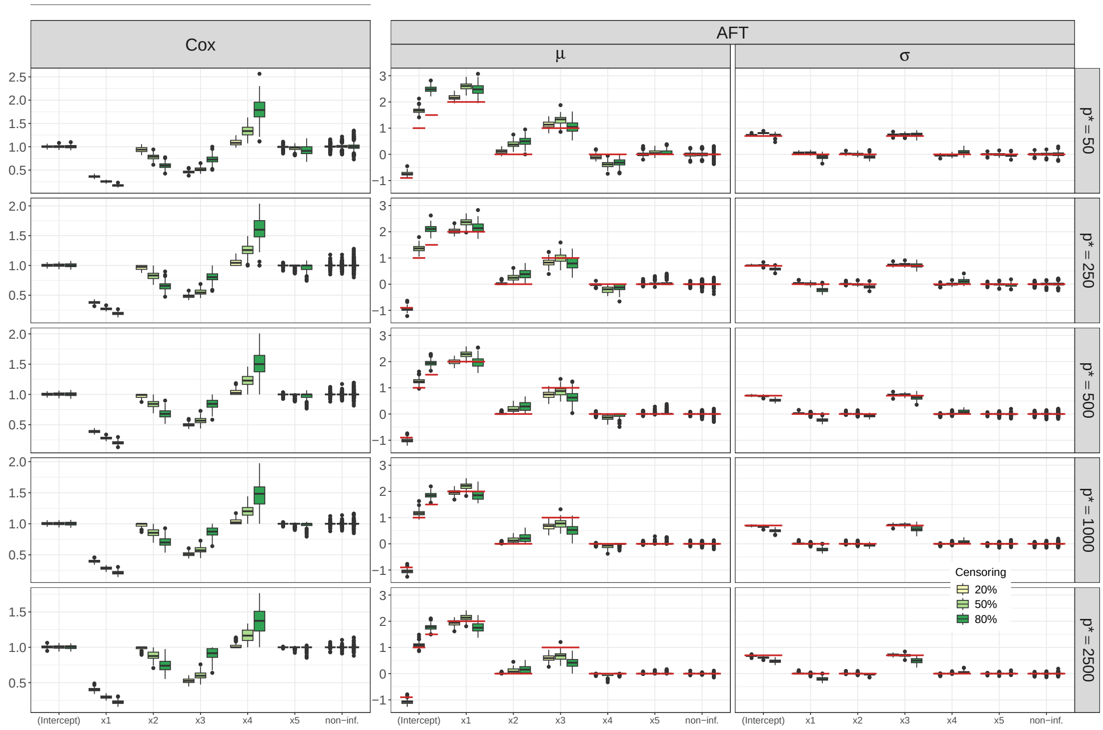

Fig. A16 Setting 1: Boxplot of estimated coefficients of the Cox and AFT models for different numbers of noise variables on the 100 replicates. The box colors represent the average proportions of censoring. The red horizontal lines show the true values for each.

	Censoring	Copula	Cox	AFT
p* = 50	20%	108.00 (20.24)	0.94 (0.59)	1.42 (0.40)
	50%	93.67 (19.31)	0.90 (0.56)	1.75 (0.37)
	80%	102.35 (27.92)	1.47 (0.93)	1.74 (0.56)
p* = 250	20%	61.08 (14.22)	0.72 (0.12)	0.57 (0.22)
	50%	58.26 (13.16)	0.74 (0.12)	1.36 (0.38)
	80%	65.48 (17.62)	0.88 (0.38)	2.10 (0.42)
p* = 500	20%	45.19 (16.30)	0.88 (0.16)	0.85 (0.32)
	50%	48.26 (13.37)	0.86 (0.34)	1.29 (0.50)
	80%	36.07 (12.63)	0.80 (0.43)	1.42 (0.60)
p* = 1000	20%	26.85 (6.72)	0.85 (0.24)	0.71 (0.38)
	50%	27.04 (13.53)	0.78 (0.12)	1.68 (0.39)
	80%	25.41 (9.04)	0.83 (0.29)	1.79 (0.60)
p* = 2500	20%	16.28 (4.33)	0.59 (0.05)	0.68 (0.19)
	50%	13.05 (1.46)	0.53 (0.03)	1.52 (0.25)
	80%	15.52 (2.65)	0.55 (0.04)	1.80 (0.35)

Table A34 Setting 1: Means (SDs) of the runtime for CopBoostDepCens, the Cox and AFT models of the Gaussian copula with log-normal distributed margins on the 100 replicates.

	Parameter	Censoring 20%							Censoring 50%							Censoring 80%						
		(Int)	x_1	x_2	x_3	x_4	x_5	non-inf.	(Int)	x_1	x_2	x_3	x_4	x_5	non-inf.	(Int)	x_1	x_2	x_3	x_4	x_5	non-inf.
p* = 50	μ_T	100	100	87	100	100	86	81.6	100	100	76	100	86	87	78.4	100	100	81	100	74	87	63.0
	σ_T	100	66	56	100	89	77	65.6	100	100	50	100	49	66	58.4	100	96	54	100	39	51	45.8
	μ_C	100	60	100	53	100	39	45.4	100	59	100	50	100	48	55.2	100	48	100	59	100	60	52.6
	σ_C	100	66	100	51	57	61	45.8	100	77	100	49	55	56	59.6	100	62	100	61	65	62	60.0
	ρ	100	100	7	6	19	100	5.0	100	100	7	5	6	100	4.8	100	29	33	12	100	100	3.4
p* = 250	μ_T	100	100	63	100	100	60	49.2	100	100	41	100	64	72	43.2	100	100	57	100	38	69	21.0
	σ_T	100	25	20	100	70	51	26.2	100	98	9	100	22	36	16.8	100	96	18	100	6	36	9.6
	μ_C	100	21	100	6	100	4	5.2	100	42	100	10	100	12	11.8	100	14	100	15	100	15	15.0
	σ_C	100	5	100	13	30	10	6.8	100	51	100	20	12	21	20.2	100	25	100	33	23	23	24.0
	ρ	100	100	0	0	1	82	0.0	100	100	0	0	0	100	0.0	100	9	17	0	100	88	0.0
p* = 500	μ_T	100	100	62	100	100	50	36.0	100	100	25	100	59	67	28.2	100	100	38	100	17	62	14.4
	σ_T	100	11	14	100	64	39	12.6	100	98	5	100	9	35	6.8	100	95	19	100	4	33	4.4
	μ_C	100	6	100	1	100	1	1.4	100	34	100	3	100	4	4.8	100	4	100	6	100	9	8.0
	σ_C	100	1	100	6	26	3	1.0	100	38	100	12	5	9	10.2	100	14	100	28	15	13	16.2
	ρ	100	100	0	0	0	33	0.0	100	100	0	0	0	96	0.0	100	9	17	0	100	70	0.0
p* = 1000	μ_T	100	100	51	100	100	28	22.0	100	100	12	100	57	59	16.8	100	100	25	100	7	59	8.8
	σ_T	100	8	11	100	42	20	3.8	100	98	0	100	2	29	2.2	100	94	15	100	3	28	2.0
	μ_C	100	0	100	0	100	0	0.0	100	30	100	0	100	1	1.2	100	2	100	5	100	8	5.0
	σ_C	100	0	99	3	24	1	0.0	100	20	100	10	2	4	4.8	100	7	100	22	10	5	11.4
	ρ	100	100	0	0	0	1	0.0	100	100	0	0	0	85	0.0	100	8	17	0	100	34	0.0
p* = 2500	μ_T	100	100	0	100	0	1	0.0	100	100	0	99	0	0	0.0	100	100	0	42	1	0	0.0
	σ_T	100	40	22	100	1	12	6.0	100	1	11	100	55	2	2.6	100	15	1	100	5	1	0.8
	μ_C	100	70	100	0	100	58	0.0	100	6	100	17	100	27	1.2	100	53	100	92	100	5	2.6
	σ_C	100	42	79	10	100	7	1.2	100	18	100	8	99	22	2.0	100	2	100	2	77	50	5.4
	ρ	100	0	0	0	0	13	0.0	100	0	0	0	0	79	0.0	100	0	0	0	100	37	0.0

Table A35 Setting 1: Selection rates for CopBoostDepCens of the Gaussian copula with log-normal distributed margins on the 100 replicates.

B.1.2 Setting 3

	Censoring	Brier score			Integrated Brier score		
		Cox	AFT	Copula	Cox	AFT	
p* = 50	20%	0.21 (0.02)	0.69 (0.11)	0.32 (0.25)	0.29 (0.07)	0.35 (0.33)	0.35 (0.33)
	50%	0.22 (0.03)	0.69 (0.13)	0.36 (0.23)	0.34 (0.08)	0.40 (0.29)	0.39 (0.29)
	80%	0.19 (0.03)	0.58 (0.17)	0.35 (0.20)	0.30 (0.11)	0.39 (0.24)	0.39 (0.24)
p* = 250	20%	0.22 (0.03)	0.69 (0.10)	0.33 (0.25)	0.33 (0.09)	0.38 (0.34)	0.37 (0.35)
	50%	0.25 (0.03)	0.70 (0.12)	0.37 (0.23)	0.40 (0.08)	0.42 (0.30)	0.41 (0.30)
	80%	0.21 (0.03)	0.56 (0.17)	0.33 (0.19)	0.36 (0.11)	0.38 (0.23)	0.37 (0.23)
p* = 500	20%	0.24 (0.03)	0.70 (0.12)	0.38 (0.28)	0.36 (0.09)	0.44 (0.37)	0.44 (0.38)
	50%	0.27 (0.03)	0.70 (0.13)	0.37 (0.23)	0.46 (0.10)	0.45 (0.31)	0.44 (0.31)
	80%	0.21 (0.03)	0.58 (0.17)	0.36 (0.22)	0.40 (0.10)	0.42 (0.26)	0.41 (0.26)
p* = 1000	20%	0.24 (0.03)	0.70 (0.11)	0.37 (0.28)	0.38 (0.08)	0.42 (0.37)	0.42 (0.37)
	50%	0.29 (0.03)	0.70 (0.12)	0.37 (0.24)	0.49 (0.09)	0.43 (0.31)	0.42 (0.31)
	80%	0.22 (0.04)	0.55 (0.14)	0.33 (0.18)	0.43 (0.11)	0.40 (0.24)	0.39 (0.25)

Table A36 Setting 3: Mean (SD) of the Brier score and integrated Brier score for CopBoostDepCens, the Cox and the AFT models on the 100 replicates of the independent setting for the Gaussian copula with log-normal margins for different numbers of noise variables.

	Censoring	Integrated absolute error: Survival time Copula	Cox	AFT	Censoring time Copula	Integrated squared error: Survival time Copula	Cox	AFT	Censoring time Copula
p* =50	20%	1.61 (0.72)	3.11 (2.13)	0.57 (0.27)	3.33 (0.38)	0.14 (0.07)	0.43 (0.41)	0.02 (0.01)	1.00 (0.18)
	50%	6.43 (2.61)	5.55 (2.93)	1.58 (0.67)	2.44 (0.35)	1.13 (0.53)	0.83 (0.58)	0.08 (0.04)	0.49 (0.13)
	80%	5.78 (2.08)	3.10 (2.13)	1.21 (0.61)	0.49 (0.07)	1.80 (0.63)	0.61 (0.61)	0.10 (0.08)	0.06 (0.02)
p* = 250	20%	2.20 (0.81)	3.01 (1.77)	1.13 (0.52)	3.57 (0.30)	0.25 (0.10)	0.38 (0.30)	0.07 (0.04)	1.15 (0.17)
	50%	7.91 (2.22)	5.53 (3.66)	3.07 (1.13)	2.95 (0.22)	1.71 (0.43)	0.85 (0.89)	0.28 (0.13)	0.72 (0.09)
	80%	6.02 (2.11)	2.86 (1.94)	2.52 (1.26)	0.59 (0.06)	2.03 (0.64)	0.55 (0.53)	0.39 (0.25)	0.09 (0.02)
p* = 500	20%	2.32 (0.88)	3.06 (2.45)	1.28 (0.54)	3.67 (0.41)	0.29 (0.11)	0.40 (0.43)	0.09 (0.04)	1.23 (0.18)
	50%	8.62 (3.10)	6.02 (4.26)	3.94 (1.88)	3.06 (0.24)	1.90 (0.61)	0.92 (0.87)	0.42 (0.24)	0.78 (0.09)
	80%	6.56 (2.69)	3.44 (2.91)	3.09 (1.74)	0.64 (0.07)	2.22 (0.86)	0.72 (0.94)	0.53 (0.36)	0.10 (0.02)
p* = 1000	20%	2.54 (0.93)	3.07 (2.3)	1.48 (0.58)	3.75 (0.35)	0.34 (0.11)	0.38 (0.37)	0.11 (0.05)	1.28 (0.17)
	50%	9.29 (3.95)	6.5 (5.68)	4.56 (2.69)	3.19 (0.23)	2.16 (0.75)	1.01 (1.05)	0.53 (0.34)	0.85 (0.10)
	80%	6.91 (3.16)	3.76 (3.41)	3.48 (2.17)	0.67 (0.06)	2.35 (0.93)	0.80 (0.95)	0.62 (0.44)	0.11 (0.02)

Table A37 Setting 3: Means (SDs) of the integrated absolute and integrated squared error for CopBoostDepCens, the Cox and the AFT models on the 100 replicates of the independent setting for the Gaussian copula with log-normal margins for different numbers of noise variables.

	Censoring	Copula	Cox	AFT
p* = 50	20%	55.76 (29.93)	0.58 (0.35)	0.79 (0.19)
	50%	48.93 (25.10)	0.55 (0.34)	0.93 (0.14)
	80%	39.90 (25.54)	0.57 (0.31)	0.99 (0.11)
p* = 250	20%	26.09 (6.47)	0.50 (0.08)	0.39 (0.17)
	50%	26.28 (4.85)	0.50 (0.03)	0.57 (0.14)
	80%	22.36 (5.59)	0.46 (0.04)	0.63 (0.16)
p* = 500	20%	20.44 (6.23)	0.48 (0.01)	0.33 (0.08)
	50%	21.22 (4.93)	0.44 (0.03)	0.45 (0.11)
	80%	17.42 (4.60)	0.41 (0.02)	0.49 (0.13)
p* = 1000	20%	18.14 (4.60)	0.63 (0.01)	0.34 (0.11)
	50%	17.74 (5.55)	0.46 (0.16)	0.45 (0.16)
	80%	16.65 (5.80)	0.45 (0.08)	0.52 (0.17)

Table A38 Setting 3: Means (SDs) of the runtime for CopBoostDepCens, the Cox and AFT models of the Gaussian copula with log-normal distributed margins on the 100 replicates.

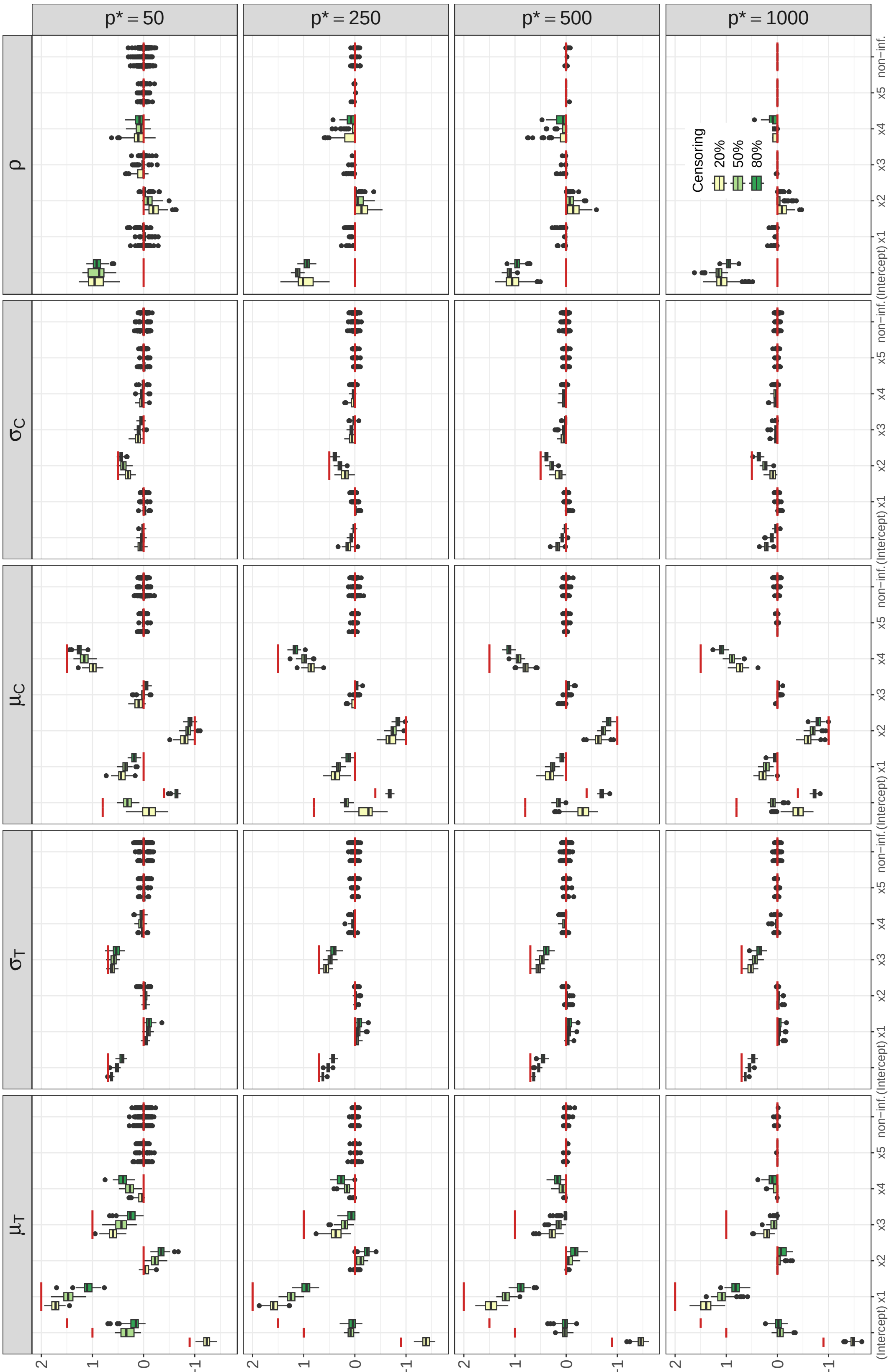

Fig. A17 Setting 3: Results of the estimated linear effects for the independent setting for the Gaussian copula with log-normal margins for different numbers of covariates p from 100 replicates. The horizontal red lines correspond to the true values.

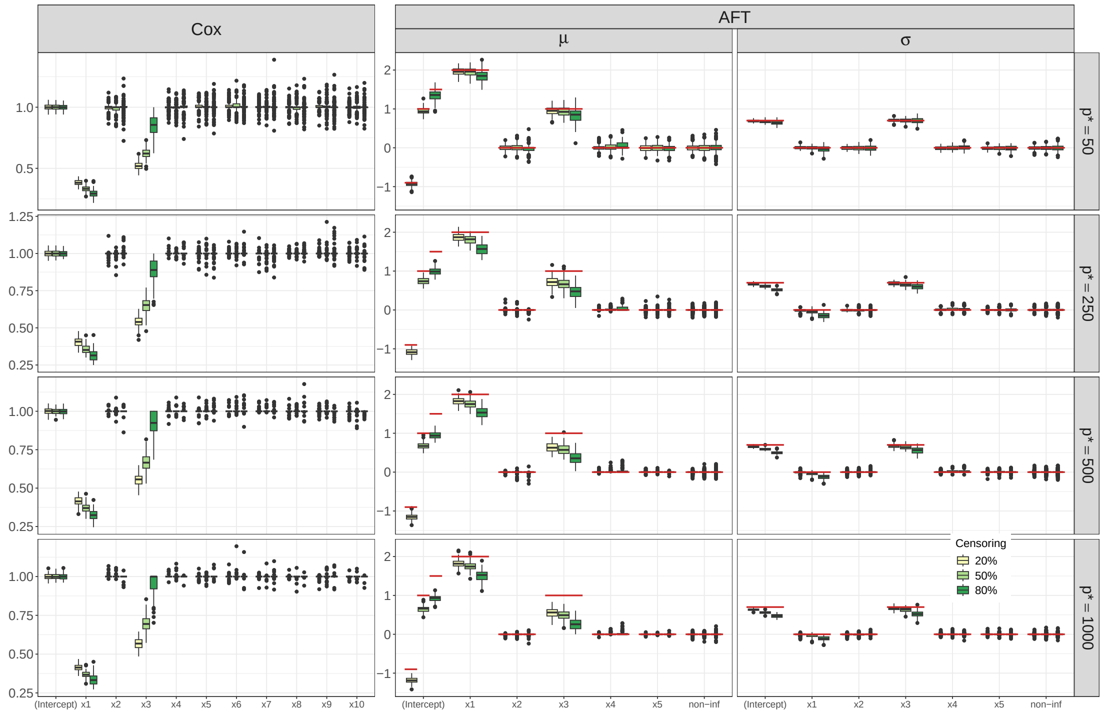

Fig. A18 Setting 3: Boxplot of estimated coefficients of the Cox and AFT models for different numbers of noise variables on the 100 replicates. The box colors represent the average proportions of censoring. The red horizontal lines show the true values for each.

		Censoring 20%							Censoring 50%							Censoring 80%						
	Parameter	(Int)	x_1	x_2	x_3	x_4	x_5	non-inf.	(Int)	x_1	x_2	x_3	x_4	x_5	non-inf.	(Int)	x_1	x_2	x_3	x_4	x_5	non-inf.
p* = 50	μ_T	100	100	63	100	60	54	48.8	100	100	98	100	100	60	53.0	100	100	100	95	100	43	37.2
	σ_T	100	92	84	100	79	81	77.2	100	98	83	100	92	77	74.0	100	95	60	100	72	68	65.0
	μ_C	100	100	100	81	100	47	52.6	100	100	100	84	100	74	66.6	100	100	100	84	100	71	65.4
	σ_C	100	66	100	95	78	62	64.6	100	71	100	100	94	73	75.8	100	63	100	90	81	69	69.2
	ρ	100	31	90	51	74	31	35.6	100	48	79	53	75	42	41.8	100	40	37	28	81	30	30.8
p* = 250	μ_T	100	100	14	100	12	15	12.6	100	100	83	100	95	11	10.6	100	100	98	69	100	12	5.0
	σ_T	100	76	53	100	58	38	42.0	100	90	54	100	71	35	31.6	100	90	19	100	40	24	21.0
	μ_C	100	100	100	43	100	18	19.0	100	100	100	30	100	28	31.6	100	98	100	76	100	32	32.2
	σ_C	100	37	98	75	56	17	25.0	100	45	100	96	71	31	34.4	100	37	100	61	33	31	38.2
	ρ	100	8	74	25	47	3	3.8	100	7	62	4	37	1	2.6	100	20	32	1	75	4	3.2
p* = 500	μ_T	100	100	3	100	4	6	3.6	100	100	64	98	72	3	4.6	100	100	88	38	92	1	2.6
	σ_T	100	66	46	100	38	31	29.2	100	89	49	100	68	23	21.0	100	80	9	100	31	16	14.0
	μ_C	100	100	100	25	100	5	5.8	100	100	100	37	100	14	20.0	100	91	100	67	100	23	22.6
	σ_C	100	31	99	75	75	18	13.6	100	23	100	85	80	30	25.4	100	26	100	50	36	34	27.4
	ρ	100	9	76	9	33	1	0.6	100	2	64	3	42	0	0.2	100	25	23	2	76	0	0.8
p* = 1000	μ_T	100	100	0	100	1	0	0.8	100	100	39	77	47	1	1.8	100	100	69	13	79	0	0.2
	σ_T	100	67	46	100	17	15	18.2	100	67	57	100	51	17	13.6	100	67	9	100	24	8	7.0
	μ_C	100	99	100	2	100	0	3.0	100	100	100	33	100	7	10.4	100	74	100	53	100	9	10.6
	σ_C	100	26	89	64	70	12	6.6	100	13	100	74	74	18	16.0	100	22	100	32	32	23	21.6
	ρ	100	11	69	2	34	0	0.0	100	3	42	0	21	0	0.0	100	16	22	0	74	0	0.0

Table A39 Setting 3: Selection rates for CopBoostDepCens of the independent setting for the Gaussian copula with log-normal distributed margins on the 100 replicates.

B.2 Clayton copula

B.2.1 Setting 1

	Censoring	Brier score Copula	Cox	AFT	Integrated Brier score Copula	Cox	AFT
$p^* = 50$	20%	0.18 (0.01)	0.74 (0.13)	0.51 (0.31)	0.21 (0.02)	0.61 (0.41)	0.61 (0.41)
	50%	0.20 (0.01)	0.77 (0.12)	0.49 (0.28)	0.29 (0.06)	0.57 (0.35)	0.57 (0.35)
	80%	0.14 (0.02)	0.57 (0.19)	0.39 (0.23)	0.22 (0.09)	0.44 (0.29)	0.44 (0.29)
$p^* = 250$	20%	0.17 (0.01)	0.75 (0.12)	0.52 (0.32)	0.19 (0.02)	0.62 (0.41)	0.62 (0.41)
	50%	0.20 (0.01)	0.79 (0.12)	0.52 (0.29)	0.30 (0.07)	0.61 (0.35)	0.61 (0.36)
	80%	0.14 (0.02)	0.57 (0.19)	0.41 (0.25)	0.24 (0.08)	0.48 (0.31)	0.48 (0.31)
$p^* = 500$	20%	0.17 (0.00)	0.74 (0.13)	0.48 (0.33)	0.19 (0.02)	0.54 (0.41)	0.53 (0.42)
	50%	0.20 (0.01)	0.78 (0.12)	0.47 (0.29)	0.30 (0.05)	0.54 (0.35)	0.53 (0.35)
	80%	0.14 (0.01)	0.55 (0.18)	0.38 (0.23)	0.24 (0.07)	0.42 (0.27)	0.41 (0.28)
$p^* = 1000$	20%	0.17 (0.00)	0.73 (0.12)	0.47 (0.31)	0.19 (0.02)	0.56 (0.41)	0.55 (0.41)
	50%	0.21 (0.02)	0.79 (0.12)	0.52 (0.31)	0.31 (0.08)	0.58 (0.36)	0.58 (0.36)
	80%	0.14 (0.02)	0.56 (0.19)	0.39 (0.23)	0.25 (0.09)	0.45 (0.29)	0.45 (0.29)
$p^* = 2500$	20%	0.17 (0.00)	0.74 (0.12)	0.51 (0.31)	0.19 (0.01)	0.62 (0.41)	0.62 (0.41)
	50%	0.20 (0.01)	0.78 (0.12)	0.50 (0.30)	0.31 (0.06)	0.57 (0.36)	0.56 (0.36)
	80%	0.15 (0.02)	0.55 (0.19)	0.39 (0.24)	0.26 (0.09)	0.42 (0.28)	0.42 (0.28)

Table A40 Setting 1: Mean (SD) of the Brier score and integrated Brier score for CopBoostDepCens, the Cox and the AFT models on the 100 replicates of the Clayton copula with log-normal distributed margins for different numbers of noise variables.

		Integrated absolute error				Integrated squared error			
		Survival time			Censoring time	Survival time			Censoring time
	Censoring	Copula	Cox	AFT	Copula	Copula	Cox	AFT	Copula
$p^* = 50$	20%	0.46 (0.24)	9.27 (6.29)	1.43 (0.65)	2.83 (0.57)	0.01 (0.01)	1.80 (1.68)	0.06 (0.04)	0.44 (0.15)
	50%	1.60 (0.87)	20.53 (12.01)	9.17 (3.19)	0.44 (0.13)	0.04 (0.03)	5.81 (4.35)	1.29 (0.46)	0.01 (0.01)
	80%	3.15 (1.51)	11.18 (6.24)	6.38 (2.41)	0.11 (0.02)	0.35 (0.21)	4.21 (2.94)	1.49 (0.58)	0.00 (0.00)
$p^* = 250$	20%	0.78 (0.33)	8.82 (4.82)	1.37 (0.47)	2.84 (0.50)	0.02 (0.01)	1.53 (1.04)	0.05 (0.02)	0.41 (0.12)
	50%	2.96 (1.44)	23.20 (16.47)	5.80 (2.73)	0.63 (0.17)	0.13 (0.08)	6.49 (5.57)	0.54 (0.29)	0.02 (0.01)
	80%	4.53 (2.08)	13.39 (10.81)	4.40 (1.91)	0.13 (0.02)	0.66 (0.32)	5.33 (5.51)	0.80 (0.43)	0.00 (0.00)
$p^* = 500$	20%	0.97 (0.41)	9.07 (6.72)	1.48 (0.53)	2.56 (0.45)	0.03 (0.02)	1.58 (1.53)	0.06 (0.02)	0.32 (0.10)
	50%	3.41 (1.63)	21.38 (14.89)	4.73 (2.06)	0.72 (0.19)	0.18 (0.10)	5.81 (4.71)	0.38 (0.17)	0.02 (0.01)
	80%	4.88 (2.46)	12.25 (9.00)	3.49 (1.48)	0.13 (0.03)	0.8 (0.45)	4.7 (4.37)	0.52 (0.26)	0.00 (0.00)
$p^* = 1000$	20%	1.21 (0.47)	9.25 (6.34)	1.64 (0.59)	2.30 (0.49)	0.04 (0.02)	1.59 (1.46)	0.08 (0.03)	0.25 (0.10)
	50%	3.78 (1.50)	20.07 (10.86)	4.38 (1.47)	0.81 (0.21)	0.22 (0.11)	5.32 (3.66)	0.33 (0.12)	0.03 (0.01)
	80%	5.16 (2.13)	11.71 (7.03)	3.21 (1.21)	0.13 (0.02)	0.91 (0.40)	4.38 (3.31)	0.43 (0.18)	0.00 (0.00)
$p^* = 2500$	20%	1.58 (0.49)	8.84 (4.59)	1.86 (0.58)	1.86 (0.37)	0.07 (0.03)	1.43 (0.91)	0.10 (0.03)	0.14 (0.05)
	50%	4.32 (1.94)	22.68 (24.08)	4.58 (2.20)	0.88 (0.21)	0.28 (0.12)	6.32 (9.15)	0.34 (0.15)	0.03 (0.01)
	80%	6.07 (3.84)	15.21 (21.49)	3.62 (2.54)	0.13 (0.03)	1.11 (0.65)	6.20 (11.16)	0.45 (0.29)	0.00 (0.00)

Table A41 Setting 1: Means (SDs) of the integrated absolute and integrated squared error for CopBoostDepCens, the Cox and the AFT models on the 100 replicates of the Clayton copula with log-normal distributed margins for different numbers of noise variables.

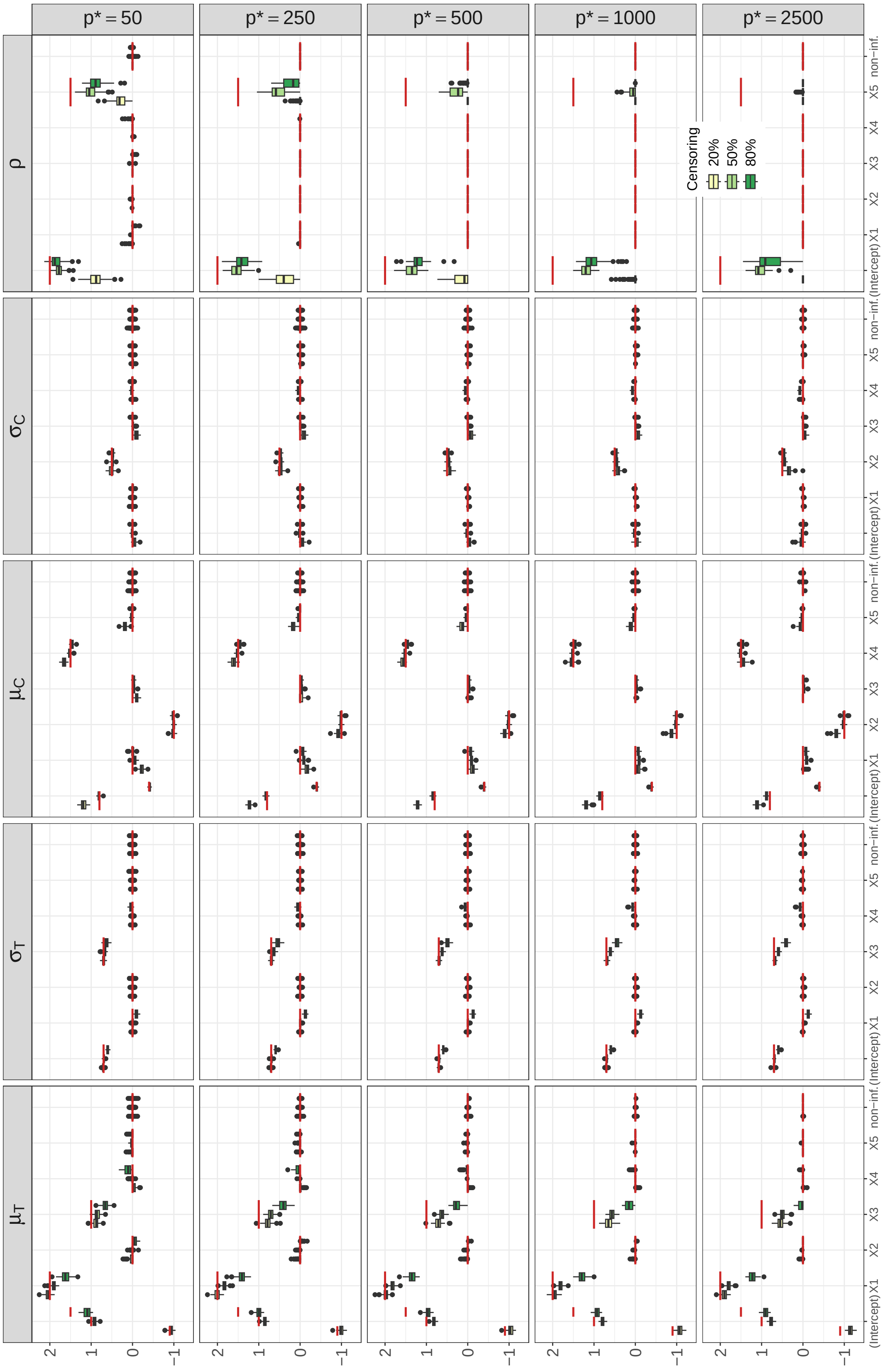

Fig. A19 Setting 1: Results of the estimated linear effects for the Clayton copula with log-normal margins for different numbers of covariates p from 100 replicates. The horizontal red lines correspond to the true values.

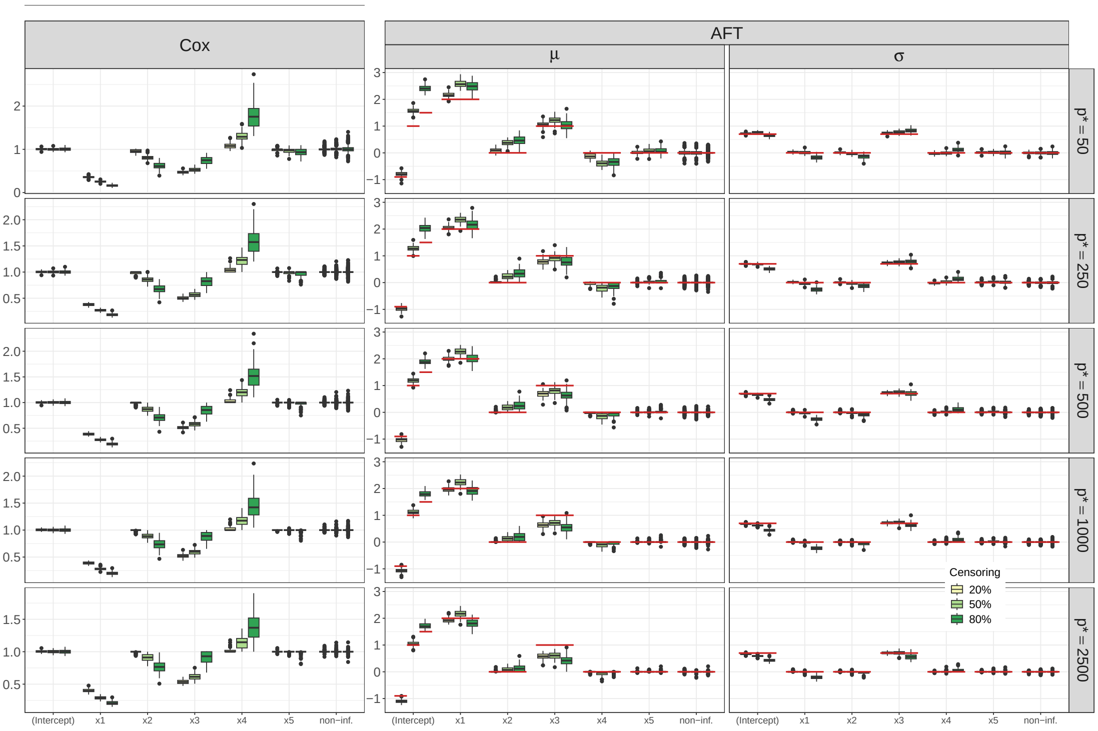

Fig. A20 Setting 1: Boxplot of estimated coefficients of the Cox and AFT models for different numbers of noise variables on the 100 replicates. The box colors represent the average proportions of censoring. The red horizontal lines show the true values for each.

	Censoring	Copula	Cox	AFT
p* = 50	20%	139.80 (16.46)	0.95 (0.64)	1.34 (0.45)
	50%	105.89 (17.60)	0.93 (0.58)	1.76 (0.38)
	80%	117.72 (23.74)	1.30 (0.86)	1.98 (0.64)
p* = 250	20%	97.11 (24.39)	0.75 (0.13)	0.62 (0.24)
	50%	61.61 (13.07)	0.69 (0.20)	0.96 (0.30)
	80%	47.76 (12.74)	0.71 (0.28)	1.16 (0.24)
p* = 500	20%	72.53 (19.36)	0.77 (0.33)	0.40 (0.15)
	50%	68.01 (12.31)	0.85 (0.37)	1.23 (0.58)
	80%	51.11 (10.51)	0.88 (0.46)	1.52 (0.62)
p* = 1000	20%	88.17 (24.53)	1.00 (0.46)	0.76 (0.49)
	50%	74.49 (12.10)	0.88 (0.34)	1.75 (0.64)
	80%	49.66 (10.22)	0.75 (0.27)	2.02 (0.53)
p* = 2500	20%	45.31 (8.46)	0.56 (0.07)	0.64 (0.21)
	50%	42.82 (6.59)	0.47 (0.02)	1.22 (0.30)
	80%	29.17 (7.68)	0.49 (0.10)	1.40 (0.38)

Table A42 Setting 1: Means (SDs) of the runtime for CopBoostDepCens, the Cox and AFT models of the Clayton copula with log-normal distributed margins on the 100 replicates.

	Parameter	Censoring 20% (Int)	x_1	x_2	x_3	x_4	x_5	non-inf.	Censoring 50% (Int)	x_1	x_2	x_3	x_4	x_5	non-inf.	Censoring 80% (Int)	x_1	x_2	x_3	x_4	x_5	non-inf.
p* = 50	μ_T	100	100	50	100	58	43	24.8	100	100	32	100	34	57	34.6	100	100	75	100	93	42	30.0
	σ_T	100	55	64	100	58	58	58.8	100	58	62	100	71	61	57.6	100	100	48	100	98	63	61.4
	μ_C	100	100	100	95	100	100	36.0	100	100	100	87	100	92	64.2	100	100	100	88	100	78	69.4
	σ_C	100	33	100	96	43	56	40.2	100	65	100	69	100	64	64.0	100	74	100	73	68	75	68.2
	ρ	100	19	1	0	3	96	0.0	100	1	5	2	0	100	4.0	100	5	0	5	16	100	2.2
p* = 250	μ_T	100	100	36	100	35	15	8.8	100	100	12	100	4	31	6.4	100	100	19	100	66	14	1.4
	σ_T	100	41	44	100	44	43	40.2	100	48	34	100	40	27	22.6	100	100	15	100	97	24	22.2
	μ_C	100	99	100	49	100	100	14.4	100	99	100	84	100	83	26.0	100	97	100	77	100	50	29.2
	σ_C	100	10	100	93	14	26	17.8	100	31	100	48	99	39	28.2	100	40	100	45	46	45	34.8
	ρ	88	1	0	0	0	21	0.0	100	0	0	0	0	99	0.0	100	0	0	0	1	81	0.0
p* = 500	μ_T	100	100	27	100	32	5	4.2	100	100	6	100	1	8	1.2	100	100	4	98	42	7	0.2
	σ_T	100	21	32	100	27	32	29.0	100	45	25	100	32	11	15.4	100	100	9	100	97	15	9.0
	μ_C	100	99	100	21	100	99	5.8	100	98	100	83	100	80	15.8	100	95	100	76	100	44	16.8
	σ_C	100	5	100	90	8	12	10.2	100	19	100	43	99	33	16.8	100	23	100	36	37	31	23.2
	ρ	60	0	0	0	0	0	0.0	100	0	0	0	0	92	0.0	100	0	0	0	0	26	0.0
p* = 1000	μ_T	100	100	12	100	17	1	1.4	100	100	3	100	0	2	0.6	100	100	2	91	20	0	0.2
	σ_T	100	18	30	100	21	26	22.0	100	39	19	100	28	8	10.4	100	100	8	100	96	6	6.0
	μ_C	100	90	100	3	100	95	2.8	100	97	100	83	100	79	9.4	100	95	100	71	100	28	10.4
	σ_C	100	4	100	83	10	2	6.4	100	10	100	41	99	25	10.8	100	18	100	33	33	21	14.4
	ρ	24	0	0	0	0	0	0.0	100	0	0	0	0	61	0.0	100	0	0	0	0	1	0.0
p* = 2500	μ_T	100	100	11	100	8	0	0.4	100	100	2	100	0	1	0.0	100	100	0	56	6	0	0.0
	σ_T	100	10	12	100	11	19	11.2	100	36	12	100	25	8	5.8	100	100	6	100	96	3	1.6
	μ_C	100	29	100	0	100	75	0.2	100	97	100	77	100	74	4.8	100	93	100	50	100	16	3.6
	σ_C	100	5	100	71	15	0	0.8	100	6	100	36	99	21	6.4	100	17	100	31	28	12	7.8
	ρ	0	0	0	0	0	0	0.0	100	0	0	0	0	7	0.0	98	0	0	0	0	0	0.0

Table A43 Setting 1: Selection rates for CopBoostDepCens of the Clayton copula with log-normal distributed margins on the 100 replicates.

B.2.2 Setting 3

	Censoring	Brier score Copula	Brier score Cox	Brier score AFT	Integrated Brier score Copula	Integrated Brier score Cox	Integrated Brier score AFT
p* = 50	20%	0.17 (0.01)	0.69 (0.11)	0.32 (0.25)	0.19 (0.02)	0.35 (0.33)	0.35 (0.33)
	50%	0.18 (0.01)	0.69 (0.13)	0.36 (0.23)	0.23 (0.04)	0.40 (0.29)	0.39 (0.29)
	80%	0.15 (0.02)	0.58 (0.17)	0.35 (0.20)	0.23 (0.07)	0.39 (0.24)	0.39 (0.24)
p* = 250	20%	0.18 (0.01)	0.69 (0.10)	0.33 (0.25)	0.20 (0.03)	0.38 (0.34)	0.37 (0.35)
	50%	0.19 (0.01)	0.70 (0.12)	0.37 (0.23)	0.25 (0.04)	0.42 (0.30)	0.41 (0.3)
	80%	0.16 (0.02)	0.56 (0.17)	0.33 (0.19)	0.26 (0.07)	0.38 (0.23)	0.37 (0.23)
p* = 500	20%	0.18 (0.01)	0.70 (0.12)	0.38 (0.28)	0.21 (0.03)	0.44 (0.37)	0.44 (0.38)
	50%	0.19 (0.02)	0.70 (0.13)	0.37 (0.23)	0.27 (0.06)	0.45 (0.31)	0.44 (0.31)
	80%	0.16 (0.02)	0.58 (0.17)	0.36 (0.22)	0.28 (0.07)	0.42 (0.26)	0.41 (0.26)
p* = 1000	20%	0.19 (0.01)	0.70 (0.11)	0.37 (0.28)	0.22 (0.02)	0.42 (0.37)	0.42 (0.37)
	50%	0.20 (0.02)	0.7 (0.12)	0.37 (0.24)	0.28 (0.07)	0.43 (0.31)	0.42 (0.31)
	80%	0.17 (0.03)	0.55 (0.14)	0.33 (0.18)	0.29 (0.08)	0.4 (0.24)	0.39 (0.25)

Table A44 Setting 3: Mean (SD) of the Brier score and integrated Brier score for CopBoostDepCens, the Cox and the AFT models on the 100 replicates of the independent setting for the Clayton copula with log-normal distributed margins for different numbers of noise variables.

		Integrated absolute error				Integrated squared error			
		Survival time			Censoring time	Survival time			Censoring time
	Censoring	Copula	Cox	AFT	Copula	Copula	Cox	AFT	Copula
p* =50	20%	0.96 (0.47)	3.11 (2.13)	0.57 (0.27)	1.90 (0.30)	0.05 (0.03)	0.43 (0.41)	0.02 (0.01)	0.33 (0.08)
	50%	3.99 (1.74)	5.55 (2.93)	1.58 (0.67)	1.49 (0.21)	0.43 (0.22)	0.83 (0.58)	0.08 (0.04)	0.19 (0.04)
	80%	3.96 (1.58)	3.10 (2.13)	1.21 (0.61)	0.32 (0.05)	0.85 (0.36)	0.61 (0.61)	0.1 (0.08)	0.03 (0.01)
p* = 250	20%	1.48 (0.62)	3.01 (1.77)	1.13 (0.52)	2.19 (0.28)	0.11 (0.05)	0.38 (0.3)	0.07 (0.04)	0.44 (0.09)
	50%	5.06 (1.59)	5.53 (3.66)	3.07 (1.13)	1.77 (0.26)	0.70 (0.23)	0.85 (0.89)	0.28 (0.13)	0.26 (0.06)
	80%	4.64 (1.83)	2.86 (1.94)	2.52 (1.26)	0.40 (0.06)	1.22 (0.49)	0.55 (0.53)	0.39 (0.25)	0.04 (0.01)
p* = 500	20%	1.60 (0.63)	3.06 (2.45)	1.28 (0.54)	2.28 (0.27)	0.14 (0.06)	0.40 (0.43)	0.09 (0.04)	0.48 (0.07)
	50%	5.91 (2.41)	6.02 (4.26)	3.94 (1.88)	1.85 (0.23)	0.90 (0.38)	0.92 (0.87)	0.42 (0.24)	0.28 (0.06)
	80%	5.30 (2.35)	3.44 (2.91)	3.09 (1.74)	0.44 (0.06)	1.45 (0.65)	0.72 (0.94)	0.53 (0.36)	0.05 (0.01)
p* = 1000	20%	1.83 (0.7)	3.07 (2.30)	1.48 (0.58)	2.37 (0.28)	0.17 (0.06)	0.38 (0.37)	0.11 (0.05)	0.51 (0.09)
	50%	6.43 (3.18)	6.50 (5.68)	4.56 (2.69)	1.91 (0.20)	1.03 (0.47)	1.01 (1.05)	0.53 (0.34)	0.30 (0.05)
	80%	5.70 (2.85)	3.76 (3.41)	3.48 (2.17)	0.46 (0.06)	1.60 (0.75)	0.80 (0.95)	0.62 (0.44)	0.05 (0.01)

Table A45 Setting 3: Means (SDs) of the integrated absolute and integrated squared error for CopBoostDepCens, the Cox and the AFT models on the 100 replicates of the independent setting for the Clayton copula with log-normal distributed margins for different numbers of noise variables.

	Censoring	Copula	Cox	AFT
p* = 50	20%	50.77 (18.81)	0.75 (0.44)	0.81 (0.19)
	50%	42.61 (18.79)	0.67 (0.39)	0.91 (0.14)
	80%	39.95 (14.70)	0.57 (0.31)	0.99 (0.11)
p* = 250	20%	20.03 (3.59)	0.30 (0.01)	0.24 (0.08)
	50%	15.25 (2.75)	0.29 (0.01)	0.32 (0.08)
	80%	12.98 (3.43)	0.29 (0.02)	0.37 (0.08)
p* = 500	20%	18.74 (3.25)	0.30 (0.01)	0.21 (0.05)
	50%	14.04 (2.77)	0.29 (0.01)	0.30 (0.07)
	80%	10.44 (2.44)	0.28 (0.01)	0.33 (0.09)
p* = 1000	20%	26.36 (5.50)	0.48 (0.02)	0.32 (0.10)
	50%	20.20 (4.55)	0.44 (0.02)	0.46 (0.11)
	80%	14.28 (2.82)	0.42 (0.02)	0.49 (0.14)

Table A46 Setting 3: Means (SDs) of the runtime for CopBoostDepCens, the Cox and AFT models of the Clayton copula with log-normal distributed margins on the 100 replicates.

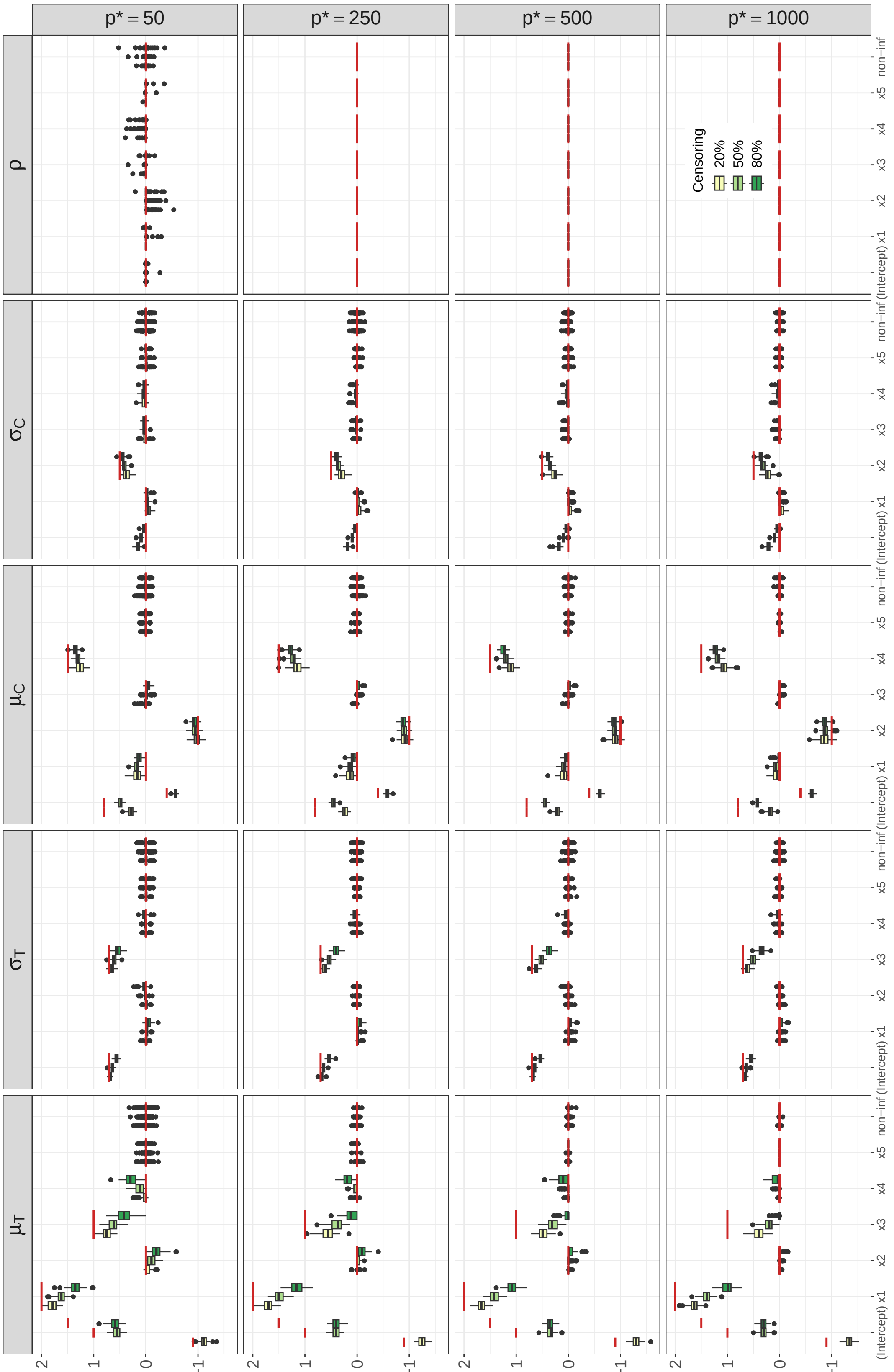

Fig. A21 Setting 3: Results of the estimated linear effects for the independent setting for the Clayton copula with log-normal margins for different numbers of covariates p from 100 replicates. The horizontal red lines correspond to the true values.

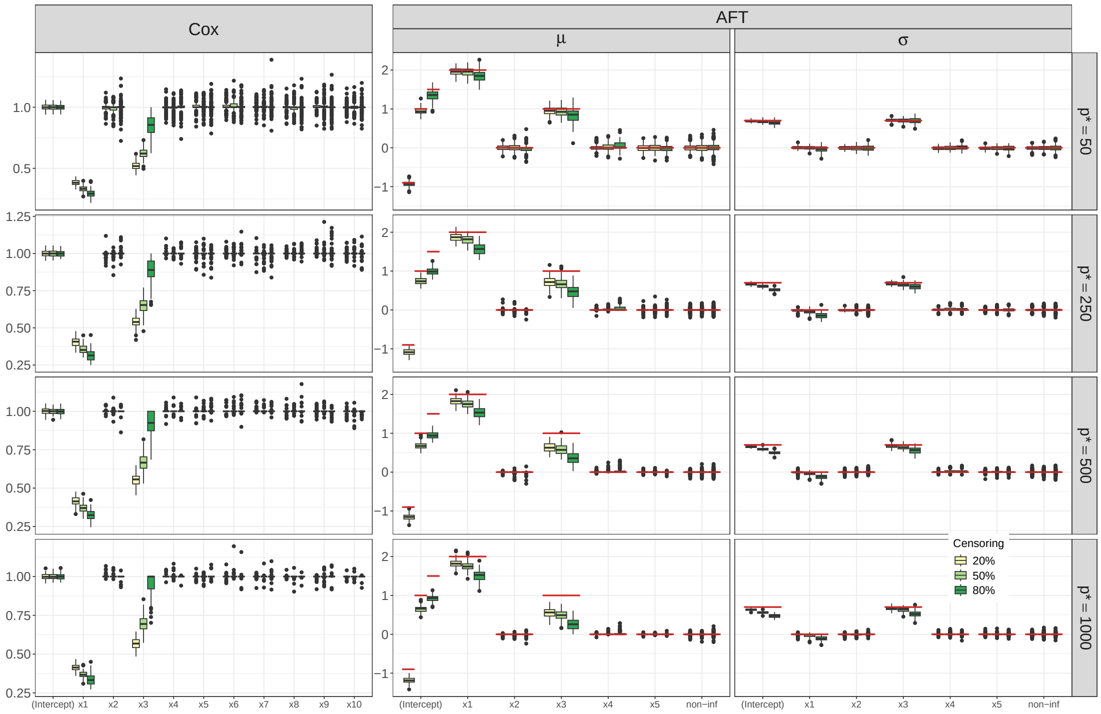

Fig. A22 Setting 3: Boxplot of estimated coefficients of the Cox and AFT models for different numbers of noise variables on the 100 replicates. The box colors represent the average proportions of censoring. The red horizontal lines show the true values for each.

	Parameter	Censoring 20% (Int)	x_1	x_2	x_3	x_4	x_5	non-inf.	Censoring 50% (Int)	x_1	x_2	x_3	x_4	x_5	non-inf.	Censoring 80% (Int)	x_1	x_2	x_3	x_4	x_5	non-inf.
p* = 50	μ_T	100	100	55	100	54	47	43.4	100	100	82	100	87	49	40.4	100	100	95	97	99	37	40.6
	σ_T	100	70	71	100	78	78	73.8	100	63	65	100	73	70	67.4	100	86	78	100	91	70	68.0
	μ_C	100	97	100	45	100	51	51.0	100	100	100	68	100	63	60.6	100	99	100	85	100	68	65.6
	σ_C	100	78	100	56	64	55	61.2	100	79	100	80	84	71	69.4	100	84	100	85	83	76	71.0
	ρ	29	0	20	7	6	1	2.2	38	4	17	3	18	2	4.4	46	5	10	7	13	3	6.2
p* = 250	μ_T	100	100	13	100	12	16	10.4	100	100	43	100	42	4	5.2	100	100	81	75	92	8	3.0
	σ_T	100	46	41	100	47	37	41.6	100	44	27	100	35	26	27.0	100	80	41	100	81	24	22.6
	μ_C	100	93	100	15	100	15	14.4	100	98	100	23	100	22	22.2	100	87	100	53	100	29	25.8
	σ_C	100	57	100	28	38	15	23.4	100	53	100	46	67	24	32.0	100	51	100	51	48	32	39.0
	ρ	0	0	0	0	0	0	0.0	0	0	0	0	0	0	0.0	0	0	0	0	0	0	0.0
p* = 500	μ_T	100	100	8	100	10	8	5.2	100	100	29	100	32	2	2.0	100	100	55	39	76	0	1.0
	σ_T	100	37	35	100	32	34	31.6	100	35	19	100	31	18	18.6	100	76	30	100	77	15	13.4
	μ_C	100	81	100	11	100	7	6.2	100	92	100	25	100	12	12.6	100	70	100	42	100	17	17.6
	σ_C	100	62	100	20	48	18	12.6	100	39	100	40	76	22	20.8	100	27	100	48	56	25	22.2
	ρ	0	0	0	0	0	0	0.0	0	0	0	0	0	0	0.0	0	0	0	0	0	0	0.0
p* = 1000	μ_T	100	100	6	100	5	0	2.4	100	100	15	94	13	0	0.4	100	100	31	14	57	0	0.0
	σ_T	100	23	38	100	26	21	20.6	100	30	26	100	20	12	11.2	100	61	24	100	80	7	7.4
	μ_C	100	71	100	1	100	2	3.2	100	82	100	15	100	5	7.8	100	49	100	30	100	4	7.6
	σ_C	100	63	99	10	43	11	7.0	100	39	100	22	70	13	12.4	100	22	100	31	50	17	19.4
	ρ	0	0	0	0	0	0	0.0	0	0	0	0	0	0	0.0	0	0	0	0	0	0	0.0

Table A47 Setting 3: Selection rates for CopBoostDepCens of the independent setting for the Clayton copula with log-normal distributed margins on the 100 replicates.

B.3 Gumbel copula

B.3.1 Setting 1

		Brier score			Integrated Brier score		
	Censoring	Copula	Cox	AFT	Copula	Cox	AFT
p* = 50	20%	0.23 (0.01)	0.74 (0.12)	0.51 (0.31)	0.39 (0.06)	0.62 (0.41)	0.61 (0.41)
	50%	0.26 (0.02)	0.78 (0.11)	0.49 (0.28)	0.45 (0.10)	0.55 (0.35)	0.55 (0.35)
	80%	0.18 (0.02)	0.53 (0.18)	0.36 (0.23)	0.30 (0.14)	0.42 (0.27)	0.41 (0.27)
p* = 250	20%	0.25 (0.01)	0.74 (0.12)	0.49 (0.31)	0.46 (0.06)	0.60 (0.41)	0.60 (0.41)
	50%	0.29 (0.03)	0.78 (0.11)	0.44 (0.27)	0.55 (0.08)	0.49 (0.33)	0.49 (0.33)
	80%	0.21 (0.03)	0.54 (0.19)	0.36 (0.22)	0.43 (0.07)	0.40 (0.26)	0.40 (0.26)
p* = 500	20%	0.26 (0.02)	0.74 (0.13)	0.48 (0.31)	0.46 (0.05)	0.59 (0.40)	0.58 (0.41)
	50%	0.31 (0.03)	0.77 (0.12)	0.45 (0.27)	0.59 (0.08)	0.51 (0.34)	0.50 (0.34)
	80%	0.22 (0.02)	0.57 (0.20)	0.42 (0.25)	0.46 (0.08)	0.49 (0.31)	0.49 (0.31)
p* = 1000	20%	0.26 (0.02)	0.73 (0.12)	0.45 (0.31)	0.47 (0.06)	0.55 (0.40)	0.54 (0.40)
	50%	0.35 (0.03)	0.78 (0.11)	0.44 (0.26)	0.66 (0.08)	0.50 (0.33)	0.49 (0.34)
	80%	0.23 (0.02)	0.53 (0.16)	0.34 (0.17)	0.50 (0.07)	0.37 (0.22)	0.37 (0.22)
p* = 2500	20%	0.26 (0.01)	0.73 (0.11)	0.44 (0.29)	0.47 (0.05)	0.57 (0.40)	0.56 (0.41)
	50%	0.38 (0.02)	0.78 (0.10)	0.43 (0.24)	0.71 (0.06)	0.49 (0.32)	0.48 (0.33)
	80%	0.25 (0.02)	0.55 (0.18)	0.38 (0.22)	0.54 (0.06)	0.43 (0.27)	0.44 (0.27)

Table A48 Setting 1: Mean (SD) of the Brier score and integrated Brier score for CopBoostDepCens, the Cox and the AFT models on the 100 replicates of the Gumbel copula with log-normal distributed margins for different numbers of noise variables.

	Censoring	Integrated absolute error: Survival time Copula	Survival time Cox	Survival time AFT	Censoring time Copula	Integrated squared error: Survival time Copula	Survival time Cox	Survival time AFT	Censoring time Copula
p* = 50	20%	1.58 (0.62)	17.16 (9.60)	2.92 (1.31)	1.02 (0.25)	0.05 (0.03)	3.71 (2.46)	0.16 (0.09)	0.10 (0.05)
	50%	5.49 (2.22)	35.53 (19.10)	15.08 (6.08)	0.66 (0.18)	0.36 (0.21)	11.65 (7.32)	2.35 (1.00)	0.03 (0.02)
	80%	6.01 (2.99)	17.64 (16.08)	8.54 (3.96)	0.17 (0.03)	0.96 (0.51)	7.69 (8.91)	1.97 (0.84)	0.01 (0.00)
p* = 250	20%	2.67 (1.01)	15.78 (11.10)	1.93 (0.90)	1.78 (0.31)	0.16 (0.07)	3.16 (2.73)	0.08 (0.05)	0.31 (0.11)
	50%	8.94 (3.50)	34.51 (22.96)	8.68 (4.46)	1.17 (0.24)	0.94 (0.42)	10.94 (8.36)	0.94 (0.54)	0.11 (0.05)
	80%	8.39 (3.20)	18.02 (13.10)	5.63 (2.73)	0.30 (0.05)	1.87 (0.68)	7.86 (6.90)	1.06 (0.60)	0.02 (0.01)
p* = 500	20%	3.03 (0.87)	15.93 (9.44)	1.92 (0.66)	2.06 (0.25)	0.20 (0.06)	3.15 (2.31)	0.08 (0.03)	0.42 (0.09)
	50%	11.59 (7.37)	42.37 (70.05)	7.34 (6.74)	1.54 (0.28)	1.47 (0.79)	13.69 (24.73)	0.63 (0.52)	0.19 (0.08)
	80%	10.13 (7.12)	26.34 (59.35)	5.13 (4.24)	0.36 (0.05)	2.38 (1.24)	12.72 (35.03)	0.76 (0.46)	0.03 (0.01)
p* = 1000	20%	3.38 (1.25)	16.83 (13.45)	2.06 (0.81)	2.22 (0.24)	0.24 (0.08)	3.26 (3.12)	0.09 (0.03)	0.48 (0.09)
	50%	13.64 (6.34)	38.56 (41.59)	6.19 (3.83)	1.97 (0.26)	2.12 (0.84)	12.09 (14.44)	0.47 (0.28)	0.32 (0.09)
	80%	9.90 (5.00)	19.79 (29.01)	4.28 (2.26)	0.41 (0.05)	2.54 (1.04)	8.76 (16.23)	0.59 (0.27)	0.04 (0.01)
p* = 2500	20%	3.68 (1.77)	18.86 (21.17)	2.34 (1.27)	2.32 (0.19)	0.27 (0.10)	3.63 (4.79)	0.11 (0.06)	0.53 (0.07)
	50%	14.49 (6.68)	36.45 (36.33)	5.61 (3.28)	2.27 (0.16)	2.52 (0.91)	11.3 (12.86)	0.40 (0.22)	0.44 (0.06)
	80%	10.23 (4.78)	18.24 (17.55)	4.29 (2.83)	0.46 (0.05)	2.78 (1.07)	7.73 (8.82)	0.58 (0.39)	0.05 (0.01)

Table A49 Setting 1: Means (SDs) of the integrated absolute and integrated squared error for CopBoostDepCens, the Cox and the AFT models on the 100 replicates of the Gumbel copula with log-normal distributed margins for different numbers of noise variables.

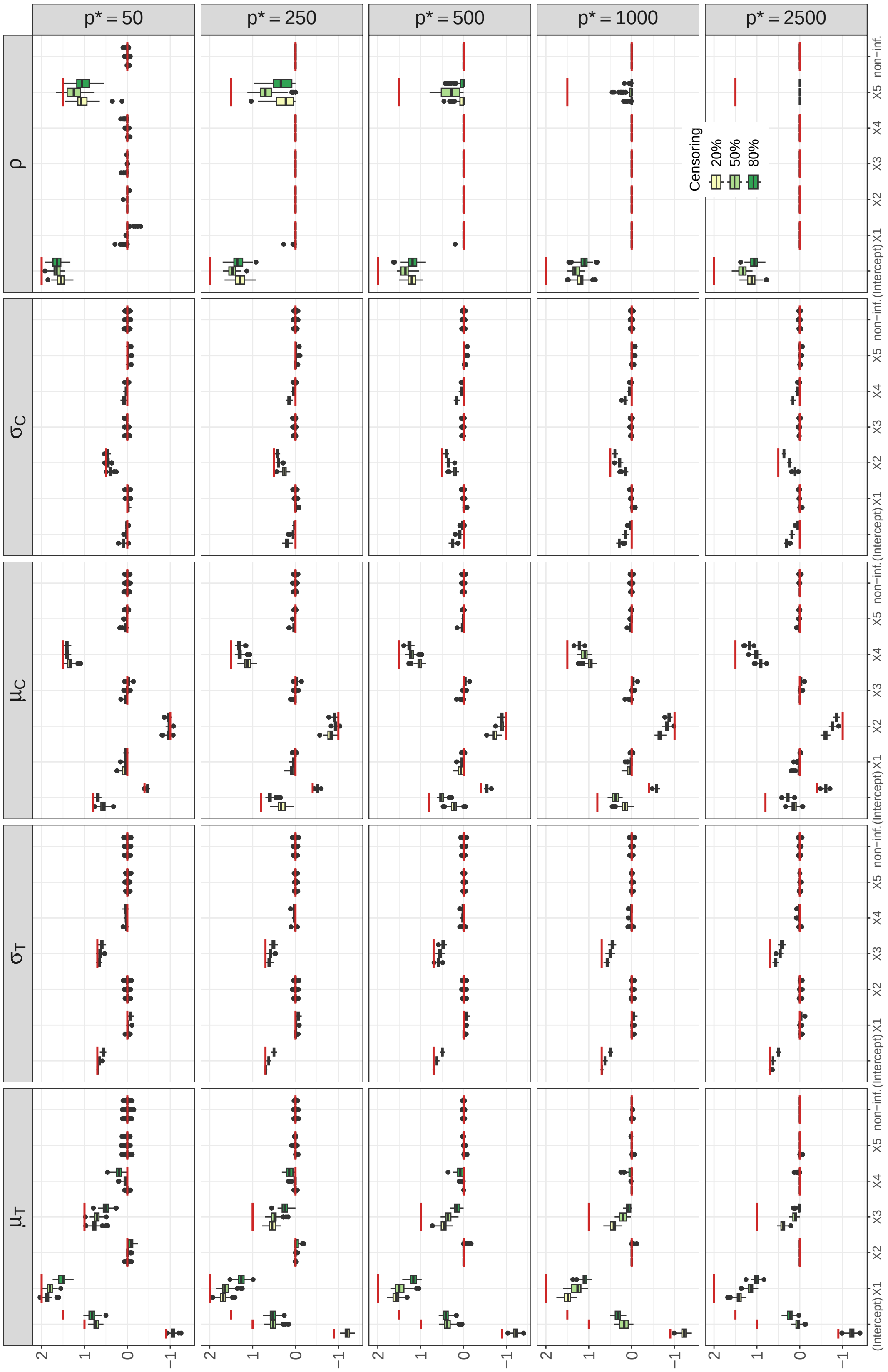

Fig. A23 Setting 1: Results of the estimated linear effects for the Gumbel copula with log-normal margins for different numbers of covariates p from 100 replicates. The horizontal red lines correspond to the true values.

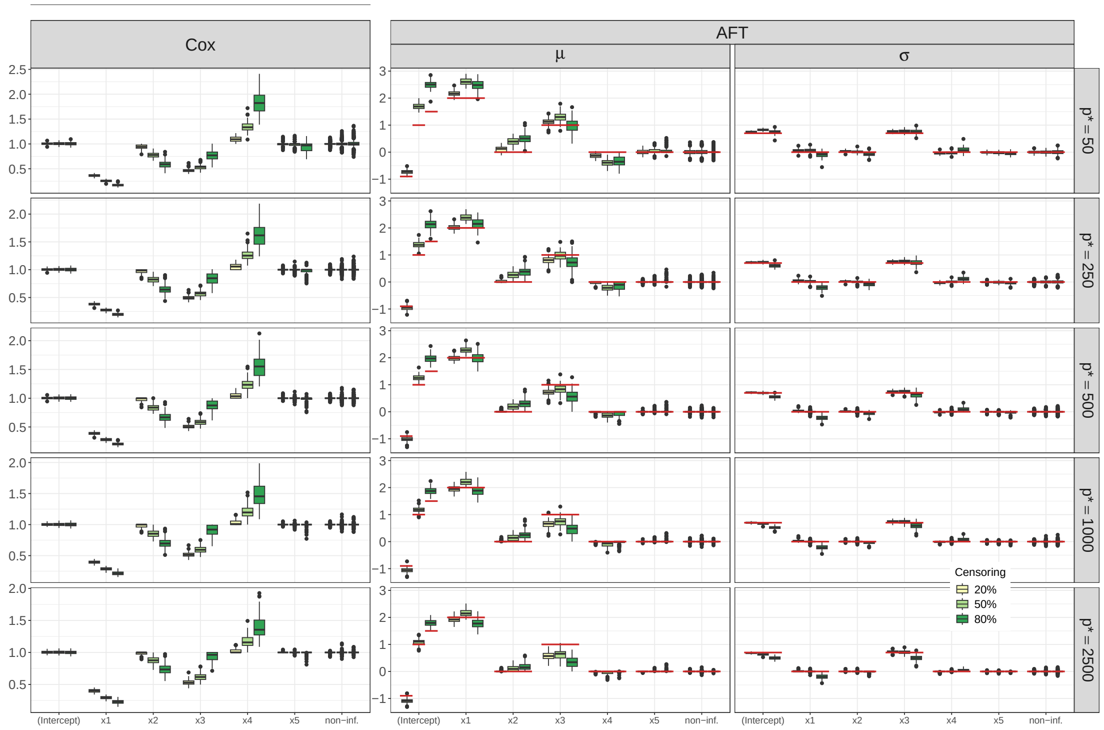

Fig. A24 Setting 1: Boxplot of estimated coefficients of the Cox and AFT models for different numbers of noise variables on the 100 replicates. The box colors represent the average proportions of censoring. The red horizontal lines show the true values for each.

	Censoring	Copula	Cox	AFT
p* = 50	20%	154.96 (30.21)	1.17 (0.90)	1.60 (0.73)
	50%	127.03 (24.75)	0.91 (0.57)	1.67 (0.34)
	80%	130.99 (25.34)	1.18 (0.70)	1.79 (0.37)
p* = 250	20%	54.72 (19.00)	0.77 (0.17)	0.47 (0.24)
	50%	79.52 (19.64)	0.85 (0.39)	1.41 (0.67)
	80%	42.11 (14.32)	0.69 (0.32)	1.06 (0.29)
p* = 500	20%	47.17 (11.55)	0.92 (0.34)	0.50 (0.20)
	50%	50.54 (18.32)	0.84 (0.31)	1.14 (0.38)
	80%	45.40 (13.04)	0.72 (0.25)	1.54 (0.39)
p* = 1000	20%	45.75 (10.02)	0.74 (0.12)	0.70 (0.22)
	50%	35.33 (12.27)	0.77 (0.12)	1.60 (0.35)
	80%	39.91 (9.64)	0.95 (0.47)	2.00 (0.71)
p* = 2500	20%	37.12 (6.69)	0.66 (0.04)	0.57 (0.14)
	50%	20.97 (3.02)	0.48 (0.02)	1.42 (0.29)
	80%	27.64 (4.53)	0.56 (0.04)	1.34 (0.35)

Table A50 Setting 1: Means (SDs) of the runtime for CopBoostDepCens, the Cox and AFT models of the Gumbel copula with log-normal distributed margins on the 100 replicates.

	Parameter	Censoring 20% (Int)	x_1	x_2	x_3	x_4	x_5	non-inf.	Censoring 50% (Int)	x_1	x_2	x_3	x_4	x_5	non-inf.	Censoring 80% (Int)	x_1	x_2	x_3	x_4	x_5	non-inf.
p* = 50	μ_T	100	100	29	100	32	38	40.0	100	100	45	100	76	43	38.0	100	100	84	100	98	40	32.6
	σ_T	100	75	73	100	89	69	74.0	100	85	69	100	98	71	68.4	100	98	66	100	85	61	63.6
	μ_C	100	85	100	72	100	90	49.4	100	85	100	66	100	75	60.8	100	84	100	98	100	70	68.2
	σ_C	100	76	100	46	100	74	57.8	100	81	100	65	99	82	65.8	100	77	100	72	73	86	67.6
	ρ	100	16	0	5	2	100	1.2	100	2	1	2	3	100	1.4	100	7	1	1	9	100	1.4
p* = 250	μ_T	100	100	5	100	4	7	7.8	100	100	9	100	45	10	10.6	100	100	49	99	92	11	5.4
	σ_T	100	52	47	100	68	42	39.0	100	72	39	100	88	40	38.4	100	99	27	100	69	31	27.4
	μ_C	100	83	100	36	100	80	11.2	100	79	100	25	100	69	29.6	100	49	100	86	100	38	35.0
	σ_C	100	46	100	12	100	49	24.8	100	48	100	34	98	63	35.4	100	42	100	33	45	66	36.0
	ρ	100	4	0	0	0	85	0.0	100	0	0	0	0	98	0.0	100	0	0	0	0	84	0.0
p* = 500	μ_T	100	100	0	100	1	9	1.6	100	100	0	100	17	4	2.2	100	100	25	93	69	5	1.0
	σ_T	100	45	38	100	55	26	27.0	100	60	34	100	81	21	20.0	100	94	15	100	60	18	14.6
	μ_C	100	77	100	16	100	79	2.8	100	74	100	14	100	57	13.6	100	25	100	86	100	20	18.6
	σ_C	100	37	100	9	100	42	13.6	100	28	100	27	98	56	22.6	100	29	100	18	37	63	25.2
	ρ	100	1	0	0	0	43	0.0	100	0	0	0	0	86	0.0	100	0	0	0	0	41	0.0
p* = 1000	μ_T	100	100	0	100	0	7	0.6	100	100	0	100	2	0	0.2	100	100	8	82	50	1	0.0
	σ_T	100	47	33	100	34	21	17.2	100	36	30	100	72	10	10.4	100	90	8	100	50	3	7.2
	μ_C	100	73	100	12	100	70	0.8	100	55	100	12	100	50	4.0	100	11	100	86	100	7	8.8
	σ_C	100	30	100	4	100	23	8.4	100	8	100	16	98	46	10.6	100	17	100	12	29	47	15.2
	ρ	100	0	0	0	0	9	0.0	100	0	0	0	0	36	0.0	100	0	0	0	0	5	0.0
p* = 2500	μ_T	100	100	0	100	0	6	0.0	100	100	0	99	0	0	0.0	100	100	0	49	21	0	0.0
	σ_T	100	42	17	100	22	9	9.8	100	20	25	100	64	6	5.4	100	81	6	100	43	1	3.6
	μ_C	100	47	100	0	100	41	0.0	100	40	100	10	100	30	1.0	100	7	100	80	100	4	3.4
	σ_C	100	16	100	2	100	11	4.6	100	3	100	17	99	34	6.4	100	13	100	8	28	36	10.6
	ρ	100	0	0	0	0	0	0.0	100	0	0	0	0	0	0.0	100	0	0	0	0	0	0.0

Table A51 Setting 1: Selection rates for CopBoostDepCens of the Gumbel copula with log-normal distributed margins on the 100 replicates.

B.3.2 Setting 3

	Censoring	Brier score Copula	Brier score Cox	Brier score AFT	Integrated Brier score Copula	Integrated Brier score Cox	Integrated Brier score AFT
p* = 50	20%	0.21 (0.02)	0.69 (0.11)	0.32 (0.25)	0.30 (0.09)	0.35 (0.33)	0.35 (0.33)
	50%	0.21 (0.03)	0.69 (0.13)	0.36 (0.23)	0.32 (0.09)	0.40 (0.29)	0.39 (0.29)
	80%	0.19 (0.03)	0.58 (0.17)	0.35 (0.20)	0.30 (0.12)	0.39 (0.24)	0.39 (0.24)
p* = 250	20%	0.23 (0.03)	0.69 (0.10)	0.33 (0.25)	0.38 (0.10)	0.38 (0.34)	0.37 (0.35)
	50%	0.24 (0.03)	0.70 (0.12)	0.37 (0.23)	0.40 (0.09)	0.42 (0.3)	0.41 (0.3)
	80%	0.21 (0.03)	0.56 (0.17)	0.33 (0.19)	0.38 (0.11)	0.38 (0.23)	0.37 (0.23)
p* = 500	20%	0.24 (0.02)	0.70 (0.12)	0.38 (0.28)	0.41 (0.09)	0.44 (0.37)	0.44 (0.38)
	50%	0.26 (0.03)	0.70 (0.13)	0.37 (0.23)	0.46 (0.10)	0.45 (0.31)	0.44 (0.31)
	80%	0.22 (0.04)	0.58 (0.17)	0.36 (0.22)	0.42 (0.11)	0.42 (0.26)	0.41 (0.26)
p* = 1000	20%	0.25 (0.02)	0.70 (0.11)	0.37 (0.28)	0.42 (0.08)	0.42 (0.37)	0.42 (0.37)
	50%	0.28 (0.03)	0.70 (0.12)	0.37 (0.24)	0.50 (0.10)	0.43 (0.31)	0.42 (0.31)
	80%	0.23 (0.04)	0.55 (0.14)	0.33 (0.18)	0.45 (0.11)	0.40 (0.24)	0.39 (0.25)

Table A52 Setting 3: Mean (SD) of the Brier score and integrated Brier score for CopBoostDepCens, the Cox and the AFT models on the 100 replicates of the independent setting for the Gumbel copula with log-normal distributed margins for different numbers of noise variables.

	Censoring	Integrated absolute error: Survival time Copula	Cox	AFT	Censoring time Copula	Integrated squared error: Survival time Copula	Cox	AFT	Censoring time Copula
p* =50	20%	1.61 (0.69)	3.11 (2.13)	0.57 (0.27)	3.27 (0.38)	0.14 (0.07)	0.43 (0.41)	0.02 (0.01)	0.95 (0.18)
	50%	6.24 (2.58)	5.55 (2.93)	1.58 (0.67)	2.23 (0.36)	1.07 (0.52)	0.83 (0.58)	0.08 (0.04)	0.39 (0.12)
	80%	5.95 (2.05)	3.1 (2.13)	1.21 (0.61)	0.46 (0.06)	1.92 (0.64)	0.61 (0.61)	0.1 (0.08)	0.05 (0.01)
p* = 250	20%	2.37 (0.87)	3.01 (1.77)	1.13 (0.52)	3.68 (0.28)	0.29 (0.10)	0.38 (0.30)	0.07 (0.04)	1.20 (0.11)
	50%	7.79 (2.21)	5.53 (3.66)	3.07 (1.13)	2.8 (0.23)	1.66 (0.42)	0.85 (0.89)	0.28 (0.13)	0.63 (0.09)
	80%	6.27 (2.16)	2.86 (1.94)	2.52 (1.26)	0.57 (0.06)	2.21 (0.68)	0.55 (0.53)	0.39 (0.25)	0.08 (0.02)
p* = 500	20%	2.47 (0.88)	3.06 (2.45)	1.28 (0.54)	3.78 (0.34)	0.33 (0.10)	0.40 (0.43)	0.09 (0.04)	1.28 (0.12)
	50%	8.67 (3.14)	6.02 (4.26)	3.94 (1.88)	2.96 (0.22)	1.93 (0.63)	0.92 (0.87)	0.42 (0.24)	0.70 (0.08)
	80%	6.82 (2.69)	3.44 (2.91)	3.09 (1.74)	0.63 (0.07)	2.39 (0.85)	0.72 (0.94)	0.53 (0.36)	0.10 (0.02)
p* = 1000	20%	2.69 (0.94)	3.07 (2.30)	1.48 (0.58)	3.86 (0.28)	0.37 (0.11)	0.38 (0.37)	0.11 (0.05)	1.32 (0.11)
	50%	9.24 (4.02)	6.50 (5.68)	4.56 (2.69)	3.07 (0.21)	2.13 (0.77)	1.01 (1.05)	0.53 (0.34)	0.76 (0.08)
	80%	7.19 (3.18)	3.76 (3.41)	3.48 (2.17)	0.67 (0.06)	2.54 (0.93)	0.80 (0.95)	0.62 (0.44)	0.11 (0.02)

Table A53 Setting 3: Means (SDs) of the integrated absolute and integrated squared error for CopBoostDepCens, the Cox and the AFT models on the 100 replicates of the independent setting for the Gumbel copula with log-normal distributed margins for different numbers of noise variables.

	Censoring	Copula	Cox	AFT
p* = 50	20%	48.83 (14.91)	0.59 (0.35)	0.82 (0.18)
	50%	45.39 (27.97)	0.53 (0.32)	0.89 (0.13)
	80%	46.60 (34.29)	0.48 (0.27)	0.96 (0.12)
p* = 250	20%	30.75 (9.04)	0.48 (0.02)	0.38 (0.12)
	50%	28.81 (6.88)	0.56 (0.03)	0.49 (0.12)
	80%	25.09 (6.40)	0.49 (0.03)	0.55 (0.13)
p* = 500	20%	23.54 (6.74)	0.48 (0.01)	0.32 (0.08)
	50%	22.60 (5.71)	0.45 (0.02)	0.47 (0.10)
	80%	21.19 (6.29)	0.46 (0.09)	0.55 (0.19)
p* = 1000	20%	20.44 (5.14)	0.49 (0.06)	0.34 (0.13)
	50%	22.04 (5.98)	0.56 (0.01)	0.47 (0.11)
	80%	18.65 (5.38)	0.49 (0.02)	0.50 (0.14)

Table A54 Setting 1: Means (SDs) of the runtime for CopBoostDepCens, the Cox and AFT models of the Gumbel copula with log-normal distributed margins on the 100 replicates.

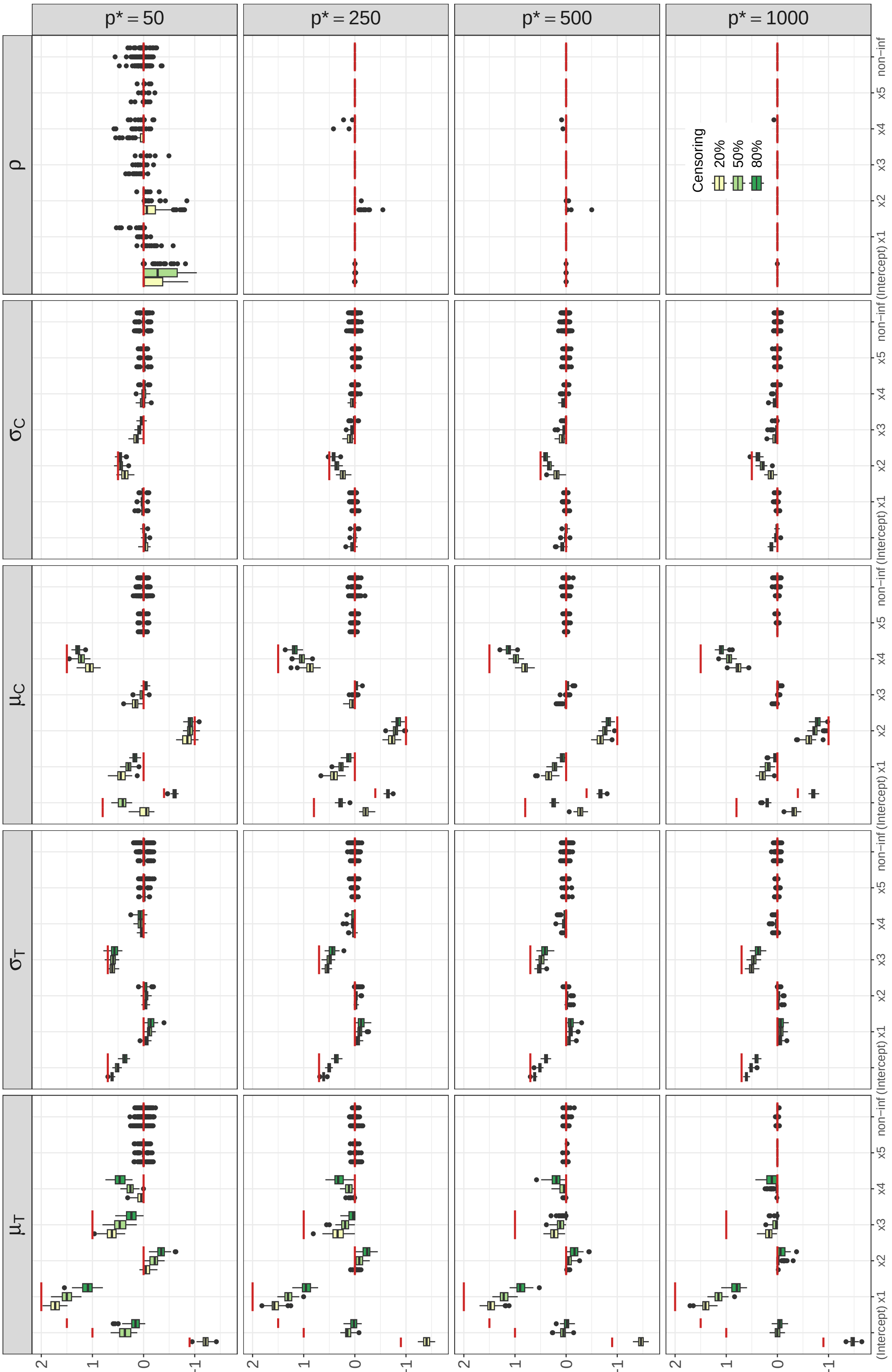

Fig. A25 Setting 3: Results of the estimated linear effects of the independent setting for the Gumbel copula with log-normal margins for different numbers of covariates p from 100 replicates. The horizontal red lines correspond to the true values.

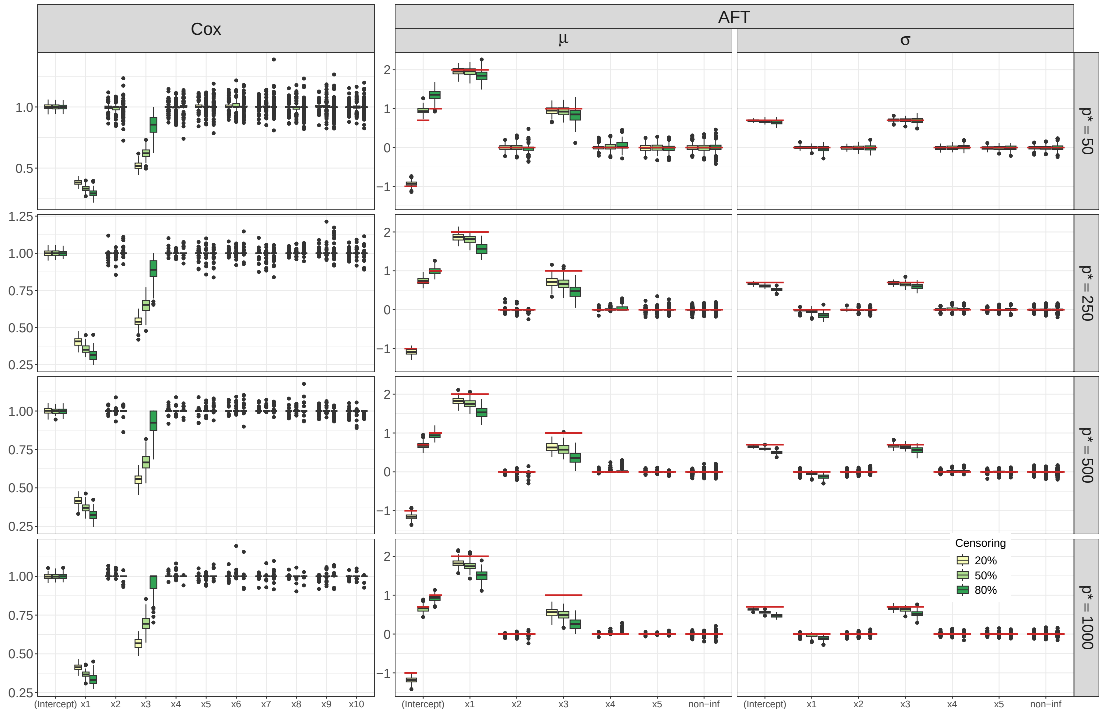

Fig. A26 Setting 3: Boxplot of estimated coefficients of the Cox and AFT models for different numbers of noise variables on the 100 replicates. The box colors represent the average proportions of censoring. The red horizontal lines show the true values for each.

		Censoring 20%							Censoring 50%							Censoring 80%						
	Parameter	(Int)	x_1	x_2	x_3	x_4	x_5	non-inf.	(Int)	x_1	x_2	x_3	x_4	x_5	non-inf.	(Int)	x_1	x_2	x_3	x_4	x_5	non-inf.
p* = 50	μ_T	100	100	72	100	72	57	52.2	100	100	96	100	100	58	50.6	100	100	100	93	100	43	40.8
	σ_T	100	96	86	100	80	86	80.6	100	98	89	100	92	75	73.8	100	98	76	100	81	66	65.0
	μ_C	100	100	100	95	100	53	61.2	100	100	100	80	100	71	69.2	100	100	100	90	100	69	65.4
	σ_C	100	70	100	98	97	75	69.4	100	78	100	97	89	77	75.4	100	71	100	90	75	76	70.6
	ρ	88	20	59	20	31	8	10.2	84	12	23	10	16	9	10.2	52	17	5	6	10	7	6.0
p* = 250	μ_T	100	100	20	99	17	18	13.8	100	100	79	98	92	14	11.2	100	100	99	63	99	9	4.8
	σ_T	100	91	55	100	65	37	44.0	100	96	61	100	74	36	32.0	100	96	23	100	51	21	22.0
	μ_C	100	100	100	65	100	19	20.6	100	100	100	20	100	31	28.2	100	96	100	66	100	34	30.2
	σ_C	100	31	100	93	79	28	31.4	100	37	100	86	53	35	34.8	100	39	100	53	40	35	39.2
	ρ	11	0	11	0	0	0	0.0	3	0	1	0	2	0	0.0	2	0	0	0	2	0	0.0
p* = 500	μ_T	100	100	6	100	7	9	3.2	100	100	60	86	58	5	3.6	100	100	86	25	93	1	1.8
	σ_T	100	85	51	100	43	31	33.2	100	95	53	100	68	17	21.8	100	95	16	100	41	14	16.2
	μ_C	100	100	100	29	100	8	7.6	100	100	100	12	100	15	17.4	100	85	100	59	100	26	22.2
	σ_C	100	10	99	87	89	23	20.6	100	30	100	76	50	31	25.2	100	27	100	44	30	31	28.6
	ρ	3	0	3	0	0	0	0.0	3	0	2	0	1	0	0.0	1	0	0	0	1	0	0.0
p* = 1000	μ_T	100	100	1	100	1	0	0.8	100	100	37	55	31	0	1.8	100	100	65	5	79	0	0.2
	σ_T	100	85	51	100	27	17	20.8	100	89	55	100	49	15	14.4	100	80	21	100	29	9	6.4
	μ_C	100	100	100	9	100	0	3.8	100	100	100	11	100	6	9.6	100	70	100	45	100	9	11.2
	σ_C	100	10	95	78	85	14	8.8	100	23	100	61	54	24	17.8	100	25	100	28	21	22	21.6
	ρ	0	0	0	0	0	0	0.0	0	0	0	0	0	0	0.0	1	0	0	0	1	0	0.0

Table A55 Setting 3: Selection rates for CopBoostDepCens of the independent setting for the Gumbel copula with log-normal distributed margins on the 100 replicates.

C Comparison of intercept models with Czado and Van Keilegom (2023)

Model	μ_T	σ_T	μ_C	σ_C	ρ
True values	1	0.7	0.8	0.5	0.5
Boosting approach	1.073 (0.186)	0.663 (0.064)	0.834 (0.092)	0.476 (0.051)	0.374 (0.254)
Czado and Van Keilegom (2023)	1.045 (0.220)	0.653 (0.071)	0.821 (0.107)	0.478 (0.053)	0.254 (0.360)

Table A56 Simulation results for intercept-only models comparing our boosting framework with Czado and Van Keilegom (2023). Data were generated with $n = 1000$, $\rho = 0.5$ (Kendall's $\tau = 0.306$), and 100 replications. Boosting was run without early stopping to ensure comparability.

D Model selection results for the application on survival of colon cancer patients

Copula	Margins	Empirical risk
Gumbel	Weibull	5.003
	Log-normal	5.128
Clayton	Weibull	4.972
	Log-normal	5.126
Gaussian	Weibull	5.013
	Log-normal	5.130

Table A57 Negative log-likelihoods for the different combinations of margins and copulas based on the left-out folds of 10-fold cross-validation.
